# Association between polymorphisms of heat-shock protein 70 genes and noise-induced hearing loss: A meta-analysis

**DOI:** 10.1371/journal.pone.0188539

**Published:** 2017-11-27

**Authors:** Song Lei, Liu Huang, Yaqian Liu, Liangwen Xu, Dahui Wang, Lei Yang

**Affiliations:** 1 Medical School, Shihezi University, Shihezi, Xinjiang, China; 2 Medical School, Hangzhou Normal University, Hangzhou, Zhejiang, China; Duke Cancer Institute, UNITED STATES

## Abstract

**Background:**

Recent studies have evaluated the associations between polymorphisms of the heat-shock protein 70 (HSP70) encoding genes and noise-induced hearing loss (NIHL). However, the conclusions of these studies are conflicting. The objective of this meta-analysis was to clarify the association between all known polymorphisms of HSP70 genetic loci and susceptibility to NIHL, based on existing reports.

**Methods:**

We conducted a meta-analysis of the association between Hsp70 polymorphisms (**rs1043618**, **rs1061581**, **rs2075800**, **rs2227956**, and **rs2763979**) and NIHL risk in both Chinese and Caucasian males. All statistical analysis was done with was conducted using the “meta” package (version 4.6–0) of R version 3.3.2 and RStudio version 1.0.44. Online databases were searched for eligible case-control studies on February 13, 2017. The odds ratio (OR), 95% confidence interval (CI), and P value were calculated using Mantel-Haenszel statistics under a random- or fixed-effect model.

**Results:**

A total of five studies, reported via four articles from online databases, were included in our meta-analysis. For **rs1061581** (from three studies), a significant association was detected in the allele model, homozygote model, and dominant model (G versus A: OR (95% CI) = 1.32(1.05–1.67), GG versus AA: OR (95% CI) = 1.93(1.1–3.36), GG + AG versus AA: OR (95% CI) = 1.45(1.05–2.02)), but not in the heterozygote model or the recessive model. For **rs1043618** (from five studies), **rs2075800** (from two studies), **rs2227956** (from four studies), **rs2763979** (from two studies), no significant association was found for any genetic model. After subgroup analyses by ethnicity, significant associations were observed for the allele model, heterozygote model, and dominant model for **rs1061581** and any genetic model for **rs2227956** in Caucasians.

**Conclusions:**

The **rs1043618**, **rs2075800**, and **rs2763979** polymorphisms were not found to be associated with susceptibility to NIHL; however, the **rs1061581** and **rs2227956** polymorphisms were significantly associated with NIHL in Caucasian males.

## Introduction

Noise-induced hearing loss (NIHL) is a preventable acquired hearing loss caused by industrial, military, and recreational noise exposure [1]. NIHL is considered a serious public health problem; WHO estimates show that 10% of individuals worldwide that are exposed to noise may develop NIHL [2]. Furthermore, the prevalence of NIHL was 24.4% among US adults aged 20–69 years, according to the 2011–2012 National Health and Nutrition Examination Survey [3]. In addition, before 2010, 20% or more of the population in Asian countries was reported to suffer from NIHL; this rate was as high as 89% among individuals in specific occupations [4]. NIHL susceptibility is influenced by numerous genetic and environmental factors; in particular, heat-shock protein 70 (HSP70) genes, which encode a group of proteins that protect cells from oxidative stress, are implicated. Other genes considered to influence NIHL susceptibility include inner ear potassium recycling pathway genes and monogenic deafness genes [5].

Heat shock proteins (HSPs) are a class of functionally related proteins whose expression is stimulated by physiological stress, ototoxic drugs, high temperature, and noise [6]. HSP70, which is highly conserved and is the most abundant chaperone, is present in various cellular compartments [7, 8]. The HSP70 gene family comprises HSP70-1, HSP70-2, and HSP70-hom [5]. Several studies have shown that the production of HSP70 in the inner ear protects hair cells by countering the ototoxic effects of cisplatin and aminoglycosides [9–11]. The influence of HSP70 genetic polymorphisms on NIHL susceptibility has been the focus of investigations for over ten years [12–14]. However, these studies have been unable to reach a consistent conclusion.

To the best of our knowledge, there has been no systematic review or meta-analysis of the association between HSP70 genetic variants and NIHL risk to date. The present report is the first meta-analysis, based on existing reports in the literature, of the relationship between all known HSP70 gene polymorphisms and susceptibility to NIHL in noise exposed workers.

## Materials and methods

A review protocol was developed *a priori* and registered in the PROSPERO International prospective register of systematic reviews (http://www.crd.york.ac.uk/PROSPERO; registration number CRD42017062595), to provide full details of the methods used. Furthermore, the present meta-analysis was conducted in accordance with the standards of the Systematic Reviews of Genetic Association Studies [15] and the Preferred Reporting Items for Systematic Reviews and Meta-analyses (PRISMA) [16].

### Literature search strategy

Relevant studies were identified by searching PubMed, EMBASE, Web of Science, China National Knowledge Infrastructure (CNKI), Wanfang Data, and SinoMed (Chinese biomedical literature service system). The search terms were: (“Hearing Loss, Noise-Induced” or “Acoustic Trauma”) and (“HSP70 Heat-Shock Proteins” or “HSP70”) in combination with (“Polymorphism, Single Nucleotide” or “gene” or “polymorphism” or “variants or alleles”). The full search strategy is available on PROSPERO (http://www.crd.york.ac.uk/PROSPERO/display_record.asp?ID=CRD42017062595). The last search was conducted on February 13, 2017.

### Eligibility criteria

The following eligibility criteria were applied to select studies for inclusion in the meta-analysis: (1) articles evaluating the association of the HSP70 gene polymorphism with NIHL; (2) a clear definition of NIHL; (3) a case—control study; (4) published in either English or Chinese; and (5) sufficient published data for calculating odds ratios (ORs) with their 95% confidence intervals (CIs).

### Exclusion criteria

The exclusion criteria were as follows: (1) duplicate publications (only the latest publication with the most complete or updated data was selected); (2) incomplete information; (3) insufficient data; (4) review articles or conference literatures.

### Data extraction

Data were extracted by two reviewers (Song Lei and Liu Huang) independently according to the pre-specified data extraction form. The following information was extracted from each study: first author, population (country, ethnicity), source of controls, case/control sample size, genotype counts for cases and controls, and evidence of Hardy-Weinberg equilibrium (HWE). If the essential data were not reported, we attempted to contact the author of the relevant studies to obtain these. Differences, if any, were resolved by consensus after discussion.

### Quality assessment for individual studies

The Newcastle-Ottawa Scale (NOS) was used to assess the methodological quality of the individual studies by the two reviewers (Song Lei and Yaqian Liu) [17]. Each study was evaluated and scored based on three criteria: selection (4 stars), comparability (2 stars), and exposure (3 stars). The NOS point ranged from 0 to 9 stars. Disagreement, if any, was resolved by discussion with a third reviewer (Lei Yang).

### Data analysis

We first assessed the HWE in the control group using the chi-square test [18]. The P value, OR and corresponding 95% CI were calculated using Mantel–Haenszel statistics under the allele, homozygote, heterozygote, dominant, or recessive models. This differs from the published protocol as there is no explicit additive model; therefore, this was replaced with homozygote and heterozygote models. *P* values of less than 0.05 were considered to represent statistically significant associations between HSP70 gene polymorphisms and NIHL. Heterogeneity across individual studies was analyzed by the Cochran’s-*Q* statistic and the *I*^2^ statistic (*P* ≤ 0.10 and *I*^2^ ≥50% indicated the significance of heterogeneity)[19, 20]. A fixed-effect model was selected with no significant heterogeneity among studies. Otherwise, a random-effect model was used [19–22]. Subsequently, subgroup analyses were performed based on ethnicity to explore the sources of heterogeneity. The potential publication bias was evaluated by Begg’s funnel plot and Egger’s test [23]. All statistical analyses were conducted using the “meta” package (version 4.6–0) of R version 3.3.2 and RStudio version 1.0.44.

## Results

### Selection and characteristics of studies

**Fig 1:**
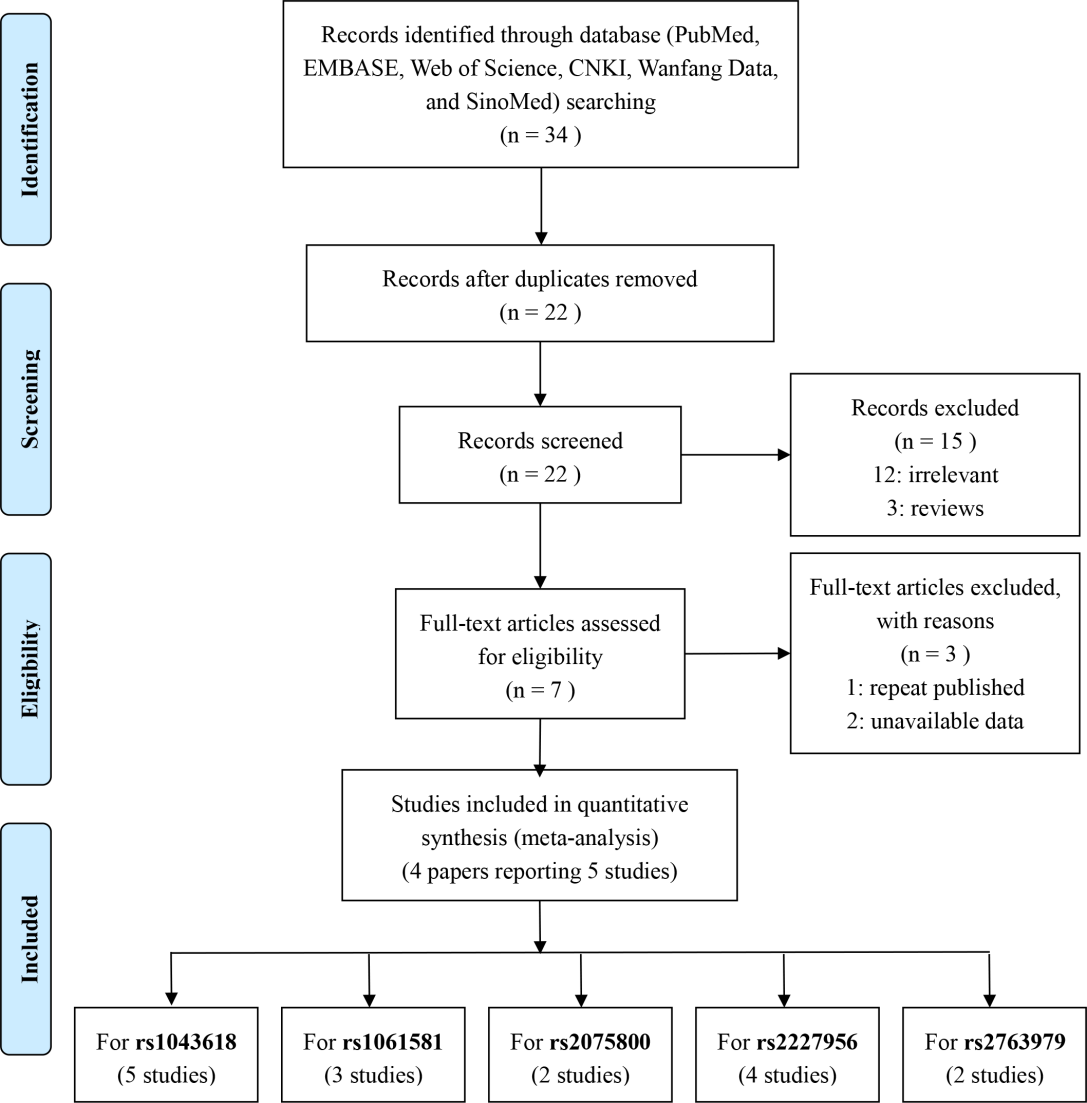
Flow chart depicting the selection of eligible studies during the meta-analysis.

A total of 34 potential articles were retrieved through electronic databases, including EMBASE (n = 5), PubMed (n = 7), Web of Science (n = 8), SinoMed (n = 1), Wanfang (n = 7), and CNKI (n = 6), during initial searching. The study selection process is detailed in Fig 1 and S1 Table. After 12 duplicated articles were removed and 15 articles were excluded by screening the title and abstract, 12 articles were found to be unrelated and three articles were reviews [5, 24, 25]. The remaining seven articles were full-text-reviewed, and three were excluded: one [6] of these was a repeat publication and two [26, 27] did not contain sufficient data. Finally, five studies reported in four articles [12–14, 28] fulfilled the inclusion criteria and were included in the present meta-analysis (the article of Konings et al. reports two studies for Sweden and Poland).

The selected study characteristics and data are listed in Table 1; there were two studies of Caucasian male populations, and one of these was hospital-based. The other studies were population-based and of Chinese populations including both males and females. In addition, among the five studies, five HSP70 gene loci polymorphisms associated with NIHL susceptibility were reported, including **rs1043618** (615 cases and 925 controls, from five studies) [12–14, 28], **rs1061581** (301 cases and 315 controls, from three studies) [13, 14], **rs2075800** (303 cases and 608 controls, from two studies) [12, 28], **rs2227956** (578 cases and 592 controls, from four studies) [12–14], and **rs2763979** (313 cases and 608 controls, from two studies) [12, 28]. Various genotyping methods were utilized, including polymerase chain (PCR)-restriction fragment length polymorphism (RFLP), TaqMan, and SNPscan. One study [14] showed that the distribution of **rs1061581** did not follow the HWE. Furthermore, the NOS scores of all studies ranged from 8 to 9 stars. This meta-analysis was carried out in accordance with the recommendations of the “Preferred Reporting Items for Systematic Reviews and Meta-Analyses” (PRISMA) statement, and Systematic Reviews of Genetic Association Studies (S1 File).

**Table 1:** Characteristics of studies included in the meta-analysis.

Author	Year	Country	Ethnicity studied	Case			Control			P-HWE	Quality
**rs1043618**				**GG**	**GC**	**CC**	**GG**	**GC**	**CC**		
Li	2017	China	Chinese	124	117	45	130	125	31	0.91	4/2/3
Chang	2011	Taiwan	Chinese	8	18	1	153	139	30	0.85	3/2/3
Konings	2009	Sweden	Caucasian	31	51	11	49	44	7	0.49	3/2/3
Konings	2009	Poland	Caucasian	44	58	14	46	58	12	0.31	3/2/3
Yang	2006	China	Chinese	37	43	13	35	48	18	0.83	4/1/3
**rs1061581**				**AA**	**AG**	**GG**	**AA**	**AG**	**GG**		
Konings	2009	Sweden	Caucasian	24	55	13	44	45	11	0.92	3/2/3
Konings	2009	Poland	Caucasian	37	61	18	43	56	15	0.63	3/2/3
Yang	2006	China	Chinese	43	41	9	50	48	3	**0.03**	4/1/3
**rs2075800**				**CC**	**TC**	**TT**	**CC**	**TC**	**TT**		
Li	2017	China	Chinese	128	128	30	112	132	42	0.76	4/2/3
Chang	2011	Taiwan	Chinese	10	15	2	113	166	43	0.14	3/2/3
**rs2227956**				**TT**	**TC**	**CC**	**TT**	**TC**	**CC**		
Li	2017	China	Chinese	204	64	8	201	73	2	0.09	4/2/3
Konings	2009	Sweden	Caucasian	64	27	0	54	39	5	0.54	3/2/3
Konings	2009	Poland	Caucasian	95	22	1	81	32	4	0.70	3/2/3
Yang	2006	China	Chinese	58	34	1	67	32	2	0.41	4/1/3
**rs2763979**				**CC**	**TC**	**TT**	**CC**	**TC**	**TT**		
Li	2017	China	Chinese	104	133	49	116	139	31	0.26	4/2/3
Chang	2011	China	Chinese	18	9	0	179	124	19	0.68	3/2/3

P-HWE: P-value for the Hardy-Weinberg equilibrium; Quality: Score of NOS scale.

### Association between polymorphism rs1043618 and NIHL susceptibility

**Fig 2:**
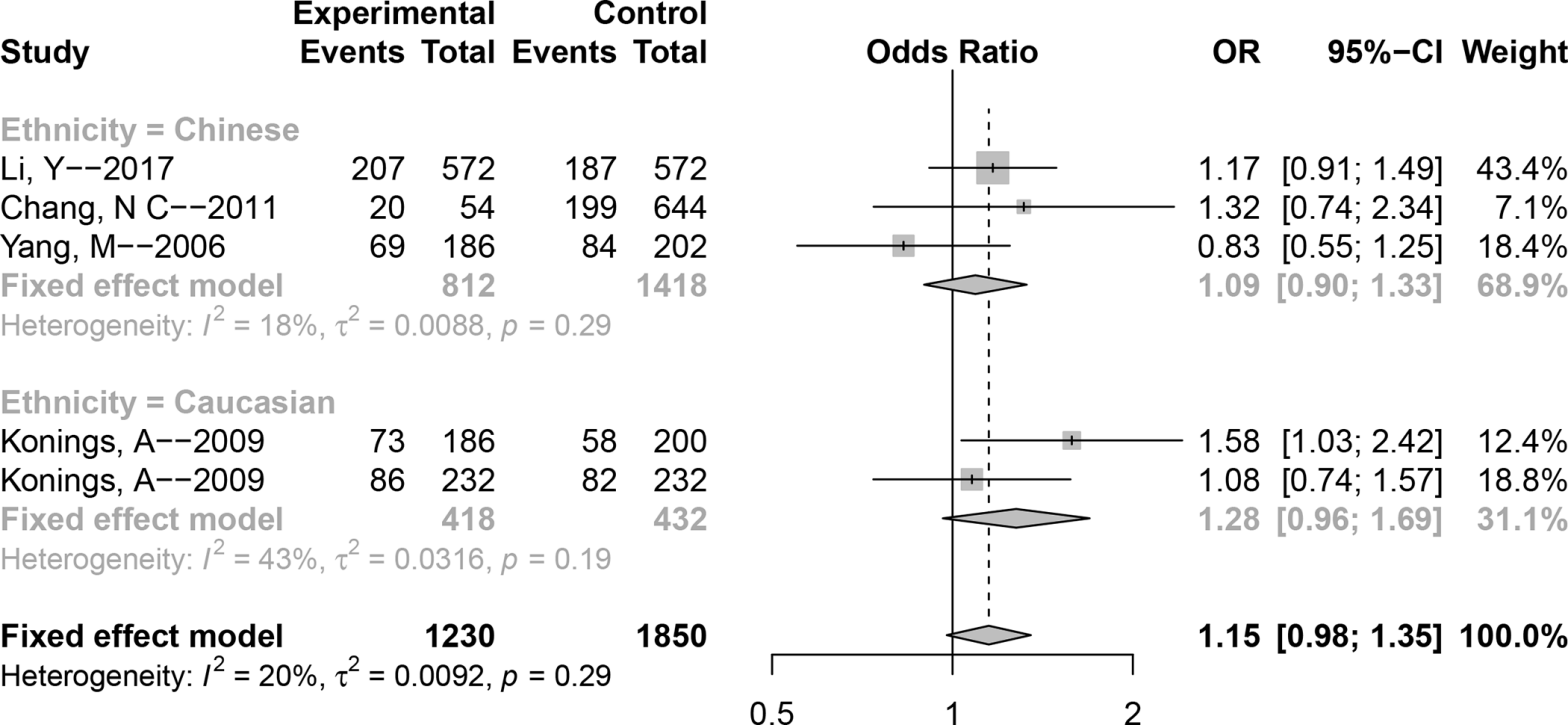
Forest plot of the association between polymorphism rs1043618 and noise-induced hearing loss (NIHL) (Allele model: C versus G).

Next, the genetic association between polymorphism **rs1043618** and susceptibility to NIHL was measured. The *P* value of Cochran’s-Q statistic for all genetic models was greater than 0.1, and *I*^2^ was less than 50%, suggesting that there was no obvious heterogeneity among the studies; therefore, the fixed-effect model was selected for pooling data. Overall, no significant association was found for any genetic model (C versus G: OR (95% CI) = 1.15 (0.98–1.35), GC versus GG: OR (95% CI) = 1.15 (0.91–1.45), CC versus GG: OR (95% CI) = 1.30 (0.91–1.86), CC + GC versus GG: OR (95% CI) = 1.19 (0.95–1.48), CC versus CG + GG: OR (95% CI) = 1.23 (0.88–1.72)). In addition, subgroup analyses by ethnicity showed no significant association in the Chinese population; this outcome was consistent with that observed in Caucasians. The main meta-analysis results are shown in detail in Fig 2 and Table 2.

**Table 2:** Summary of pooled ORs in the meta-analysis.

SNP	N	Allele model			Heterozygote model			Homozygote model			Dominant model			Recessive model		
		OR (95%CI)	*I*^2^ (%)	*P*-H	OR (95%CI)	*I*^2^ (%)	*P*-H	OR (95%CI)	*I*^2^ (%)	*P*-H	OR (95%CI)	*I*^2^ (%)	*P*-H	OR (95%CI)	*I*^2^ (%)	*P*-H
**rs1043618**		C/G			GC/GG			CC/GG			CC + GC/GG			CC/CG + GG		
Overall	5	1.15(0.98–1.35)	19.7	0.29	1.15(0.91–1.45)	44	0.13	1.30(0.91–1.86)	10.6	0.35	1.19(0.95–1.48)	39.5	0.16	1.23(0.88–1.72)	5.1	0.38
*Ethnicity*																
Chinese	3	1.09(0.90–1.33)	18.2	0.29	1.06(0.80–1.42)	54.5	0.11	1.18(0.77–1.82)	29.4	0.24	1.10(0.84–1.44)	42.6	0.17	1.16(0.78–1.73)	45.4	0.16
Caucasian	2	1.28(0.96–1.69)	42.9	0.19	1.35(0.90–1.45)	44.7	0.18	1.63(0.84–3.18)	4	0.31	1.40(0.95–2.06)	52.2	0.15	1.40(0.75–2.63)	0	0.54
**rs1061581**		G/A			AG/AA			GG/AA			GG+AG/AA			GG/AG + AA		
Overall	3	**1.32(1.05–1.67)**	0	0.62	1.38(0.98–1.94)	43.7	0.17	**1.93(1.1–3.36)**	0	0.50	**1.45(1.05–2.02)**	27.7	0.25	1.50(0.90–2.50)	0	0.38
*Ethnicity*																
Chinese	1	1.27(0.82–1.97)	-	-	0.99(0.55–1.78)	-	-	3.49(0.89–13.71)	-	-	1.14(0.65–2.00)	-	-	3.50(0.92–13.35)	-	-
Caucasian	2	**1.34(1.02–1.77)**	0	0.33	**1.64(1.07–2.49)**	41.9	0.19	1.68(0.91–3.11)	0	0.49	**1.65(1.10–2.47)**	41	0.19	1.26(0.72–2.21)	0	0.87
**rs2075800**		T/C			CT/CC			TT/CC			TT + CT/CC			TT/CT + CC		
	2	0.81(0.65–1.02)	0	0.89	0.87(0.63–1.21)	0	0.69	0.61(0.37–1.01)	0	0.84	0.81(0.60–1.10)	0	0.75	0.66(0.41–1.06)	0	0.73
**rs2227956**		C/T			TC/TT			CC/TT			CC + TC/TT			CC/TC + GG		
Overall	4	0.78(0.53–1.15)	**63.7**	**0.04**	0.80(0.62–1.04)	27.1	0.25	0.55(0.09–3.35)	**62.4**	**0.05**	0.80(0.62–1.03)	50.7	0.11	0.59(0.10–3.42)	**60.3**	**0.06**
*Ethnicity*																
Chinese	2	1.06(0.75–1.47)	0	0.86	0.96(0.69–1.33)	0	0.33	1.91(0.31–11.84)	41.3	0.19	1.01(0.74–1.39)	0	0.52	1.85(0.26–12.88)	47.7	0.17
Caucasian	2	**0.54(0.37–0.78)**	0	0.90	**0.59(0.38–0.90)**	0	0.99	**0.15(0.03–0.86)**	0	0.58	**0.53(0.35–0.81)**	0	0.91	**0.17(0.03–0.99)**	0	0.60
**rs2763979**		T/C			CT/CC			TT/CC			TT + CT/CC			TT/CT + CC		
	2	0.94(0.46–1.91)	**71.7**	**0.06**	1.00(0.72–1.39)	0	0.40	1.56(0.95–2.56)	44.6	0.18	1.08(0.80–1.48)	50	0.16	1.55(0.98–2.46)	34.4	0.22

ORs: odds ratio of the association between NIHL susceptibility and each genetic model for each single nucleotide polymorphisms (SNP); *P*-H: *P*-value for heterogeneity

### Association between polymorphism rs1061581 and NIHL susceptibility

**Fig 3:**
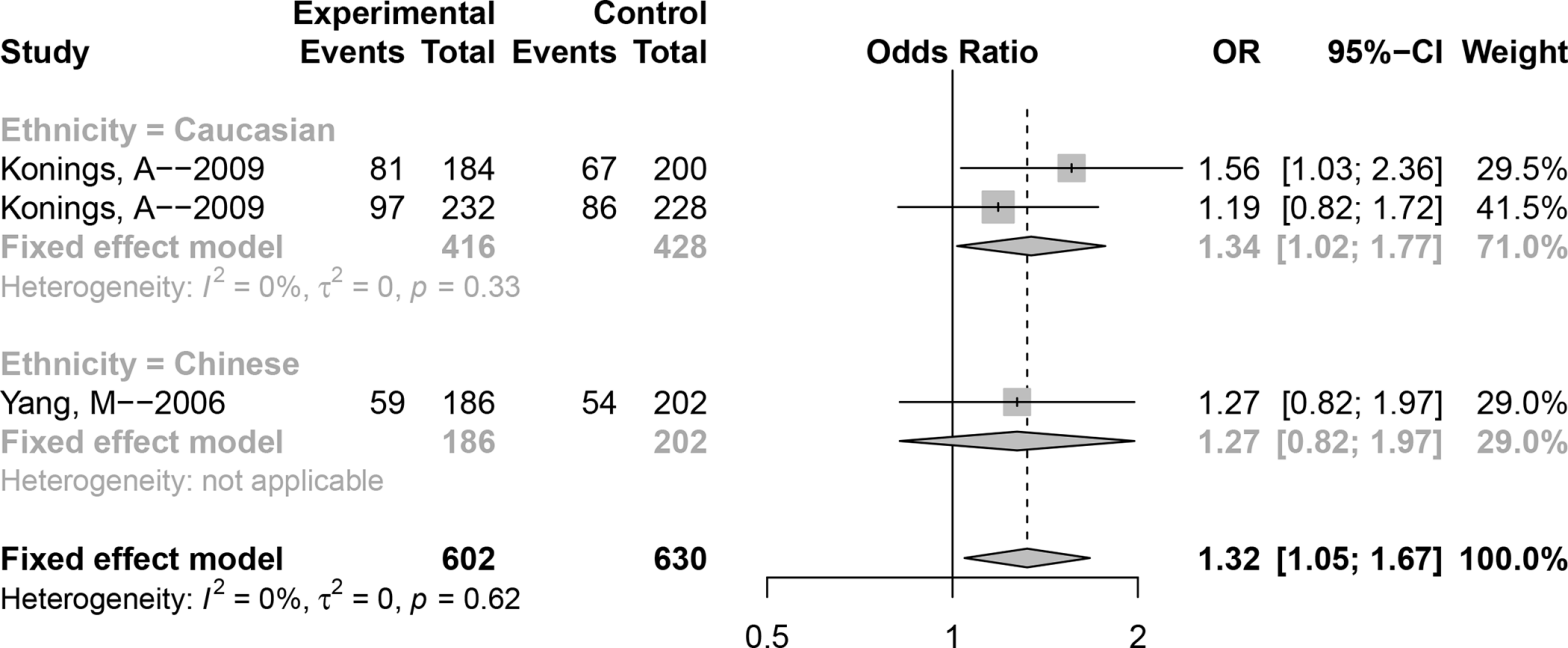
Forest plot of the association between polymorphism rs1061581 and noise-induced hearing loss (NIHL) (Allele model: G versus A).

We additionally pooled analyses of polymorphism **rs1061581** and NIHL; these are shown in Fig 3 and Table 2. The *P* value of Cochran’s-Q statistic of all genetic models were greater than 0.1 and *I*^2^ was less than 50%, indicating that there was no significant heterogeneity between the studies; therefore, the fixed-effect model was implemented. Overall, significant associations were detected in the allele model, homozygote model, and dominant model (G versus A: OR (95% CI) = 1.32 (1.05–1.67), GG versus AA: OR (95% CI) = 1.93 (1.1–3.36), GG + AG versus AA: OR (95% CI) = 1.45 (1.05–2.02)), but not in the heterozygote model or recessive model (AG versus AA: OR(95% CI) = 1.38 (0.98–1.94), GG versus AG + AA: OR (95% CI) = 1.50 (0.90–2.50)). Furthermore, subgroup analyses by ethnicity showed no significant association in the Chinese population; however, significant associations were observed for the allele model, heterozygote model, and dominant model in Caucasians.

### Association between polymorphism rs2075800 and NIHL susceptibility

**Fig 4:**
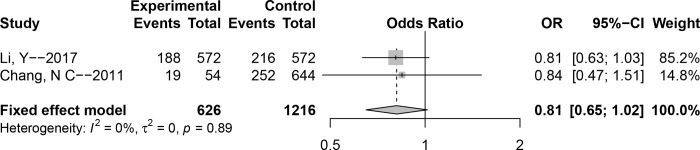
Forest plot of association between polymorphism rs2075800 and noise-induced hearing loss (NIHL) (Allele model: T versus C).

The association between polymorphism **rs2075800** and the risk of NIHL was analyzed in two Chinese studies. The *P* value of Cochran’s-*Q* statistic for all genetic models was greater than 0.1, and that of *I*^2^ less than 50%, indicating that there was no significant heterogeneity between the studies; therefore, the fixed-effect model was applied. There were no notable associations for any of the genetic models (T versus C: OR (95% CI) = 0.81 (0.65–1.02), CT versus CC: OR (95% CI) = 0.87 (0.63–1.21), TT versus CC: OR (95% CI) = 0.61 (0.37–1.01), TT + CT versus CC: OR (95% CI) = 0.81 (0.60–1.10), TT versus CT + CC: OR (95%CI) = 0.66 (0.41–1.06)) (Fig 4 and Table 2).

### Association between polymorphism rs2227956 and NIHL susceptibility

**Fig 5:**
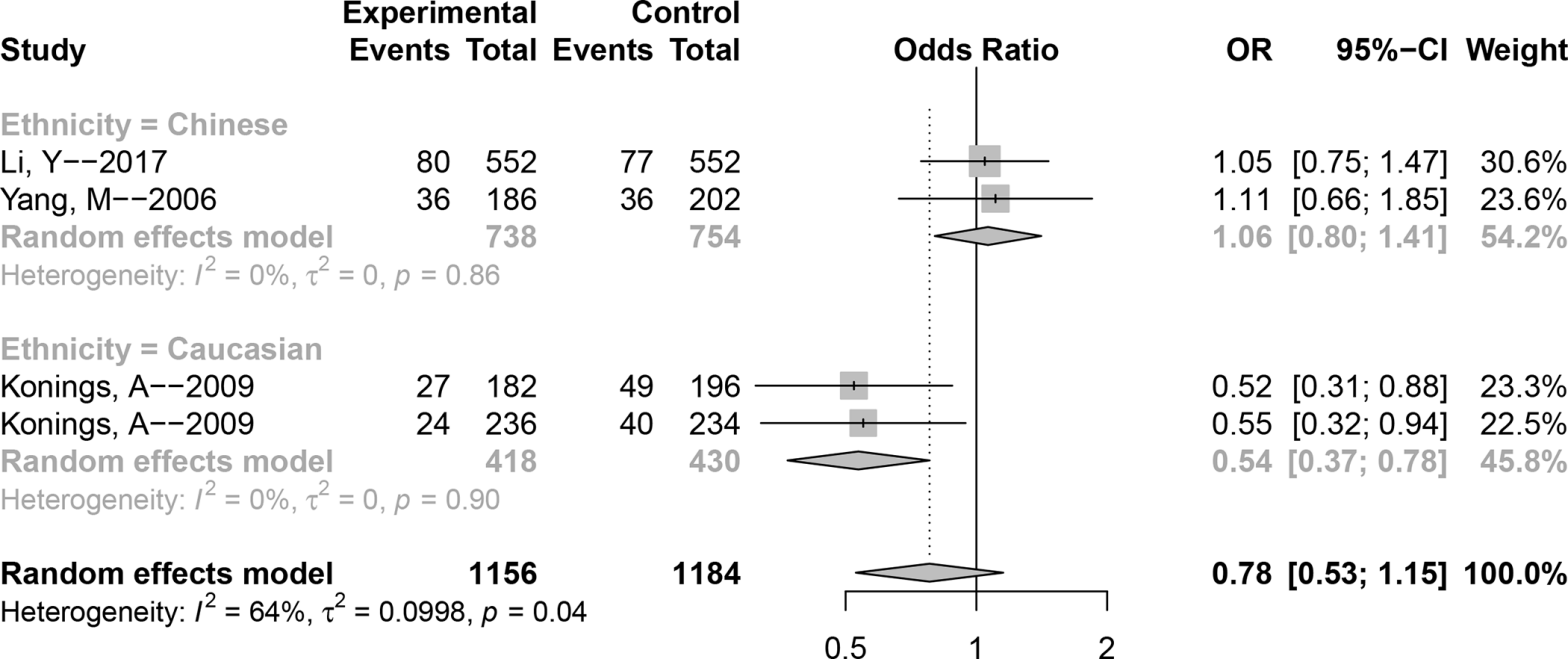
Forest plot of the association between polymorphism rs2227956 and noise-induced hearing loss (NIHL) (Allele model: C versus T).

Data describing the association between polymorphism **rs2227956** and the risk of NIHL are shown in Fig 5 and Table 2. The *P* value of Cochran’s-*Q* statistic of heterozygote model and dominant model was greater than 0.1, and that of *I*^2^ was less than 50%; therefore, the fixed-effect model was used. However, for the allele model (*P* = 0.04, *I*^2^ = 63.7%), homozygote model (*P* = 0.05, *I*^2^ = 62.4%), and recessive model (*P* = 0.06, *I*^2^ = 60.3%), the random-effect model was used. Overall, no significant association was found for any genetic model (C versus T: OR (95% CI) = 0.78 (0.53–1.15), TC versus TT: OR (95% CI) = 0.80 (0.62–1.04), CC versus TT: OR (95% CI) = 0.55 (0.09–3.35), CC + TC versus TT: OR (95% CI) = 0.80 (0.62–1.03), CC versus TC + GG: OR (95% CI) = 0.80 (0.35–1.80)). After subgroup analyses by ethnicity, the *P* value of Cochran’s-*Q* statistic of all models were greater than 0.1, and that of *I*^2^ was less than 50% in the Chinese and Caucasian populations. In addition, the association for all genetic models was significant in the Caucasian population but not in the Chinese.

### Association between polymorphism rs2763979 and NIHL susceptibility

**Fig 6:**
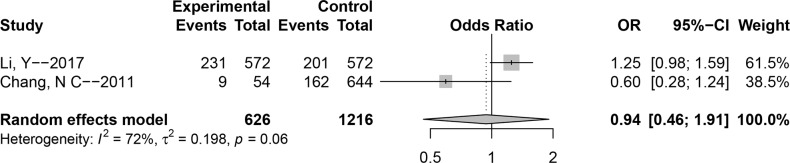
Forest plot of the association between polymorphism e rs2763979 and noise-induced hearing loss (NIHL) (allele model: T versus C).

Besides above four genetic loci, Fig 6 and Table 2 show the pooled data of the association between polymorphism **rs2763979** and NIHL susceptibility; the two studies of this polymorphism only focused on the Chinese population. The *P* value of Cochran’s-*Q* statistic was lower than 0.1, and that of *I*^2^ was more than 50% for the allele model (*P* = 0.06, *I*^2^ = 71.7%); therefore, we applied the random-effect model to this genetic model and the fixed-effect model to the other models. There were no notable associations for all genetic models (T versus C: OR (95% CI) = 0.94 (0.46–1.91), CT versus CC: OR (95% CI) = 1.00 (0.72–1.39), TT versus CC: OR (95% CI) = 1.56 (0.95–2.56), TT + CT versus CC: OR (95% CI) = 1.08 (0.80–1.48), TT versus CT + CC: OR (95% CI) = 1.55 (0.98–2.46)).

### Publication bias

**Fig 7:**
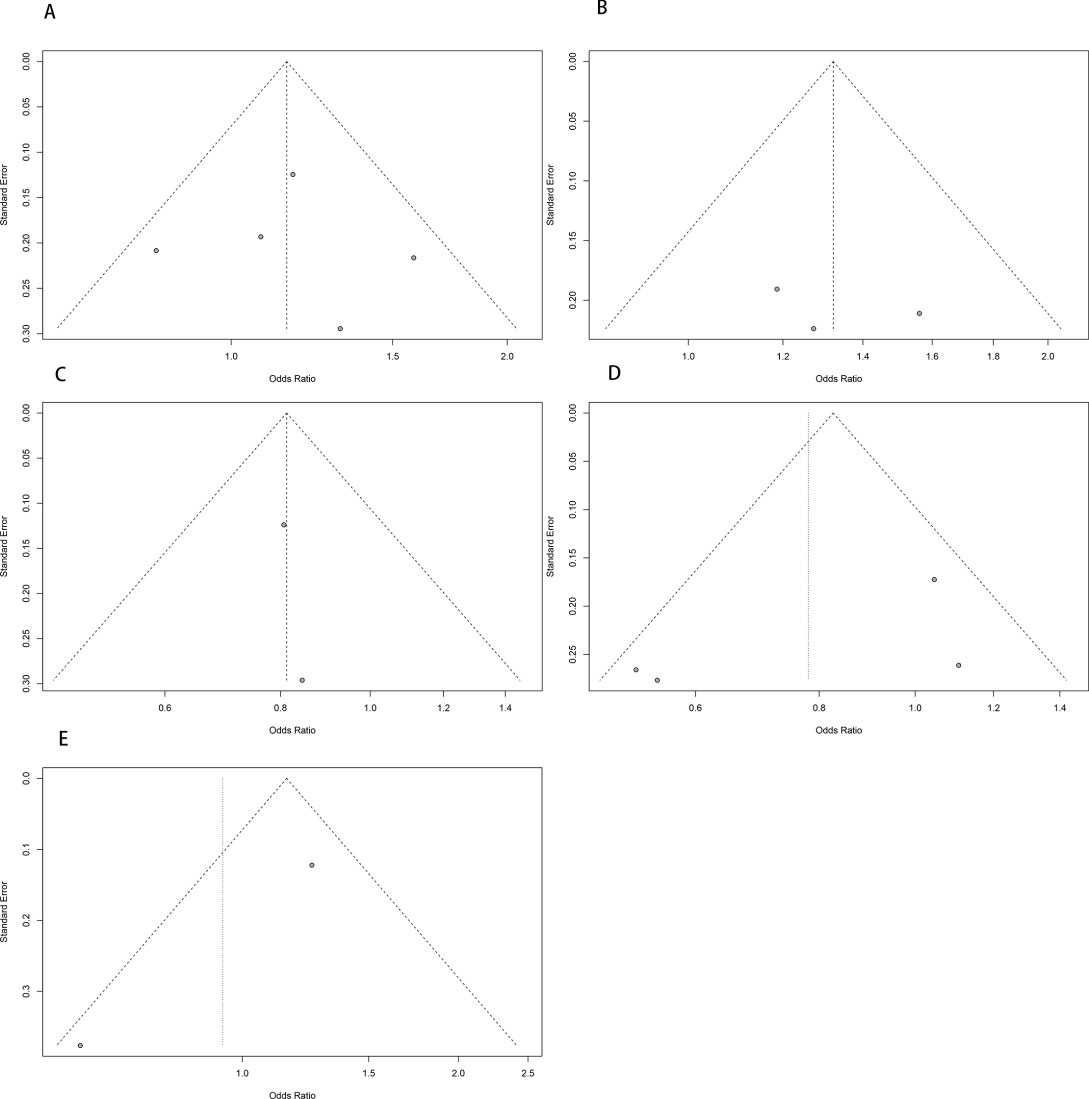
Begg's funnel plot of the association between five single nucleotide polymorphisms (SNPs) and noise-induced hearing loss (NIHL) in the allele model. A: for rs1043618; B: for rs1061581; C: for rs2075800; D: for rs2227956; E: for rs2763979.

There was no publication bias with regards to the association between rs1043618, rs1061581, and rs2227956 polymorphisms and NIHL susceptibility, as identified using the Begg’s funnel plot or Egger’s regression test (Table 3). Funnel plots of the above three genetic loci in all genetic models were symmetrical (Fig 7). Owing to the limited number of studies of rs2075800 and rs2763979 included, funnel plot analysis and the Egger’s test were not carried out.

**Table 3:** Begg’s funnel plot and Egger’s test of the meta-analysis.

SNP	Allele model		Heterozygote model		Homozygote model		Dominant model		Recessive model	
	Begg’s	Egger	Begg’s	Egger	Begg’s	Egger	Begg’s	Egger	Begg’s	Egger
rs1043618	0.62	0.88	0.14	0.23	1.00	0.62	0.62	0.41	0.62	0.28
rs1061581	0.60	0.71	0.60	0.33	0.12	0.21	0.60	0.32	0.12	0.10
rs2075800	-	-	-	-	-	-	-	-	-	-
rs2227956	0.17	0.32	0.50	0.70	0.17	0.07	0.50	0.50	0.17	0.06
rs2763979	-	-	-	-	-	-	-	-	-	-

SNP: single nucleotide polymorphism.

## Discussion

In the present meta-analysis, we evaluated the association between single nucleotide polymorphisms (SNPs) in the following HSP70 genes: HSP70-1 (**rs1043618**, 615 cases and 925 controls), HSP70-2 (**rs1061581,** 301 cases and 315 controls; **rs2763979,** 313 cases and 608 controls), HSP70-hom (**rs2075800**, 303 cases and 608 controls; **rs2227956,** 578 cases and 592 controls) and NIHL. Overall analysis indicated that there was no association between **rs1043618**, **rs2075800**, **rs2227956**, and **rs2763979** and NIHL susceptibility for any of the genetic models. However, we identified a positive relationship between **rs1061581** and NIHL in the allele model, homozygote model, and dominant model.

HSP70 is distributed in the inner ear, and has the potential to protect the hearing from noise, ototoxic drugs, or injury [29]. HSP70 overexpression can significantly protect the inner ear hair cells from cell death caused by aminoglycosides[11]. In addition, Geldanamycin, geranylgeranylacetone, and 17-Dimethylaminoethylamino-17-demethoxygeldanamycin can upregulate the expression of HSP70 to treat aminoglycoside-induced hearing loss[30–32]. Therefore, HSP70 gene polymorphisms might be associated with NIHL susceptibility. HSP70-1, HSP70-2, and HSP70-hom are located in the major histocompatibility complex (MHC) class III region of chromosome 6p21.3. Rs1043618 lies in the 5' untranslated region of HSP70-1, rs1061581 and rs2763979 are located in the 5' flanking region of HSP70-2; these loci are non-coding but perform a role in the control of gene expression. Rs2075800 and rs2227956 are located in exon 2 of HSP70-hom, the C-T of rs2075800 leads to a Glu-Lys substitution at position 602, and the A-G of rs2227956 leads to a Met-Thr substitution at position 493 (https://www.ncbi.nlm.nih.gov/snp/). All five SNPs of HSP70 genes have been explored in NIHL studies.

Two genome-wide association studies of NIHL-related SNPs in animals [33, 34] and one in humans [27] have been published; however, those studies did not examine the association between HSP70 genes and NIHL. This meta-analysis incorporates four articles that specifically investigate the association between HSP70 and NIHL; among these, only one [13] found positive results for a single SNP using crude data. However, NIHL is a complex disease that cannot be attributed to the influence of a single SNP, owing to the important role played by the interaction between genes, or between genes and the environment. Certain HSP70 haplotypes are associated with the development of NIHL [13, 14, 28]. These authors additionally found that age, smoking, drinking, cumulative noise exposure, noise exposure levels, and other factors influenced NIHL susceptibility. However, it is not possible to determine whether HSP70 is associated with NIHL based on small sample data.

To explore the heterogeneity among the studies, we conducted a subgroup analysis for **rs1043618**, **rs1061581**, and **rs2227956**. The results showed that the heterogeneity identified before subgroup analysis was reduced to an acceptable level after subgroup analysis by ethnicity, indicating that ethnicity is the main source of heterogeneity. For **rs1043618**, subgroup analysis did not reveal an association in either population. In addition, a significant association was observed between **rs1061581** and NIHL in the allele model, heterozygote model, and dominant model in the Caucasian population, but not in the other two models. Furthermore, Caucasian individuals with the G allele were more susceptible to NIHL (allele model, G versus A: OR (95% CI) = 1.32(1.05–1.67)). Similarly, a significant association was detected between **rs2227956** and NIHL in all genetic models in the Caucasian population; Caucasian individuals with the T allele were more susceptible to NIHL (allele model, C versus T: OR (95% CI) = 0.54(0.37–0.78)). However, no significant association was discovered in Chinese individuals for SNPs **rs1061581** and **rs2227956**, suggesting that neither of these polymorphisms increase the risk of NIHL. The different results for the association between **rs1061581** and **rs2227956** and NIHL in the two different populations may be attributed to differences in ethnicity.

To the best of our knowledge, the present work is the first to evaluate the association between HSP70 gene polymorphisms and NIHL susceptibility via a meta-analysis. Our meta-analysis has several strengths. We did not limit the specific loci; all previously reported SNPs were included for analysis. According to the results of NOS, the methodological quality of the study was high. Moreover, we identified the main source of heterogeneity via subgroup analysis. Finally, no publication bias was identified by either Begg’s funnel plot or Egger’s regression test, except in the case of **rs2075800** and **rs2763979**.

The present study also has several limitations. For instance, the number of included studies was very low for **rs2075800** and **rs2763979**, which limited further analysis. Secondly, Yang’s study [14] showed that the distribution of **rs1061581** did not follow the HWE; however, subgroup analysis was used separately. Thirdly, we were unable to extract sufficient adjustment data for certain factors, such as age and noise exposure level. Finally, as with three other meta-analysis studies [35–37] of the association between NIHL and other SNPs, our meta-analysis involved a small sample size, resulting in unstable pooled results.

## Conclusion

In summary, our meta-analysis comprehensively and systematically evaluated the association between HSP70 gene polymorphisms and NIHL susceptibility. The results suggested that **rs1043618**, **rs2075800**, and **rs2763979** polymorphisms are not associated with susceptibility to NIHL, while **rs1061581** and **rs2227956** polymorphisms are significantly associated with NIHL in Caucasians males. Given the limited sample size, the conclusions of this study should be treated with caution, and large sample studies are necessary in the future. These results will be useful for genetic testing of NIHL susceptibility in Caucasian males. In addition, NIHL susceptible populations can be identified by genetic testing and the corresponding measures can be taken to protect their hearing.

## Supporting information

S1 File Checklists for meta-analysis on genetic association studies. (ZIP)

S2 File Forest plot (Figure A—Figure T). (ZIP). Figure A in S2 File: Forest plot of association between the rs1043618 polymorphism and NIHL (Heterozygote model: GC versus GG). Figure B in S2 File: Forest plot of association between the rs1043618 polymorphism and NIHL (Homozygote model: CC versus GG). Figure C in S2 File: Forest plot of association between the rs1043618 polymorphism and NIHL (Dominant model: CC+GC versus GG). Figure D in S2 File: Forest plot of association between the rs1043618 polymorphism and NIHL (Recessive model: CC versus CG+GG). Figure E in S2 File: Forest plot of association between the rs1061581 polymorphism and NIHL (Heterozygote model: AG versus AA). Figure F in S2 File: Forest plot of association between the rs1061581 polymorphism and NIHL (Homozygote model: GG versus AA). Figure G in S2 File: Forest plot of association between the rs1061581 polymorphism and NIHL (Dominant model: GG+AG versus AA). Figure H in S2 File: Forest plot of association between the rs1061581 polymorphism and NIHL (Recessive model: GG versus AG+AA). Figure I in S2 File: Forest plot of association between the rs2075800 polymorphism and NIHL (Heterozygote model: CT versus CC). Figure J in S2 File: Forest plot of association between the rs2075800 polymorphism and NIHL (Homozygote model: TT versus CC). Figure K in S2 File: Forest plot of association between the rs2075800 polymorphism and NIHL (Dominant model: TT+CT versus CC). Figure L in S2 File: Forest plot of association between the rs2075800 polymorphism and NIHL (Recessive model: TT versus CT+CC). Figure M in S2 File: Forest plot of association between the rs2227956 polymorphism and NIHL (Heterozygote model: TC versus TT). Figure N in S2 File: Forest plot of association between the rs2227956 polymorphism and NIHL (Homozygote model: CC versus TT). Figure O in S2 File: Forest plot of association between the rs2227956 polymorphism and NIHL (Dominant model: CC+TC versus TT). Figure P in S2 File: Forest plot of association between the rs2227956 polymorphism and NIHL (Recessive model: CC versus TC+GG). Figure Q in S2 File: Forest plot of association between the rs2763979 polymorphism and NIHL (Heterozygote model: CT versus CC). Figure R in S2 File: Forest plot of association between the rs2763979 polymorphism and NIHL (Homozygote model: TT versus CC). Figure S in S2 File: Forest plot of association between the rs2763979 polymorphism and NIHL (Dominant model: TT+CT versus CC). Figure T in S2 File: Forest plot of association between the rs2763979 polymorphism and NIHL (Recessive model: TT versus CT+CC).
(ZIP)

S3 File Begg’s funnel plot (Figure A—Figure T). (ZIP). Figure A in S3 File: Begg’s funnel plot of association between the rs1043618 polymorphism and NIHL (Heterozygote model: GC versus GG). Figure B in S3 File: Begg’s funnel plot of association between the rs1043618 polymorphism and NIHL (Homozygote model: CC versus GG). Figure C in S3 File: Begg’s funnel plot of association between the rs1043618 polymorphism and NIHL (Dominant model: CC+GC versus GG). Figure D in S3 File: Begg’s funnel plot of association between the rs1043618 polymorphism and NIHL (Recessive model: CC versus CG+GG). Figure E in S3 File: Begg’s funnel plot of association between the rs1061581 polymorphism and NIHL (Heterozygote model: AG versus AA). Figure F in S3 File: Begg’s funnel plot of association between the rs1061581 polymorphism and NIHL (Homozygote model: GG versus AA). Figure G in S3 File: Begg’s funnel plot of association between the rs1061581 polymorphism and NIHL (Dominant model: GG+AG versus AA). Figure H in S3 File: Begg’s funnel plot of association between the rs1061581 polymorphism and NIHL (Recessive model: GG versus AG+AA). Figure I in S3 File: Begg’s funnel plot of association between the rs2075800 polymorphism and NIHL (Heterozygote model: CT versus CC). Figure J in S3 File: Begg’s funnel plot of association between the rs2075800 polymorphism and NIHL (Homozygote model: TT versus CC). Figure K in S3 File: Begg’s funnel plot of association between the rs2075800 polymorphism and NIHL (Dominant model: TT+CT versus CC). Figure L in S3 File: Begg’s funnel plot of association between the rs2075800 polymorphism and NIHL (Recessive model: TT versus CT+CC). Figure M in S3 File: Begg’s funnel plot of association between the rs2227956 polymorphism and NIHL (Heterozygote model: TC versus TT). Figure N in S3 File: Begg’s funnel plot of association between the rs2227956 polymorphism and NIHL (Homozygote model: CC versus TT). Figure O in S3 File: Begg’s funnel plot of association between the rs2227956 polymorphism and NIHL (Dominant model: CC+TC versus TT). Figure P in S3 File: Begg’s funnel plot of association between the rs2227956 polymorphism and NIHL (Recessive model: CC versus TC+GG). Figure Q in S3 File: Begg’s funnel plot of association between the rs2763979 polymorphism and NIHL (Heterozygote model: CT versus CC). Figure R in S3 File: Begg’s funnel plot of association between the rs2763979 polymorphism and NIHL (Homozygote model: TT versus CC). Figure S in S3 File: Begg’s funnel plot of association between the rs2763979 polymorphism and NIHL (Dominant model: TT+CT versus CC). Figure T in S3 File: Begg’s funnel plot of association between the rs2763979 polymorphism and NIHL (Recessive model: TT versus CT+CC).
(ZIP)

S1 Table Excluded studies and reasons for exclusion. (XLSX)
