# Correction: Association between polymorphisms of heat-shock protein 70 genes and noise-induced hearing loss: A meta-analysis

**DOI:** 10.1371/journal.pone.0242648

**Published:** 2020-11-17

**Authors:** Song Lei, Liu Huang, Yaqian Liu, Liangwen Xu, Dahui Wang, Lei Yang

Questions have been raised about similarities between this article [1] and a closely related meta-analysis [2]. The authors confirm that the work reported in [1] was completed as outlined in the article, and that they were unaware of the related work [2] prior to its publication. The results discussed in these studies align, due to the similarity in search methodologies and statistical analyses applied in both studies.

In following up on the above issue, *PLOS ONE* reassessed the article together with a member of the journal’s Editorial Board and a statistical reviewer. Several concerns were raised in this post-publication assessment which are addressed below.

1. The statistical reviewer raised concerns about the statistical model used in the published meta-analysis [1] and the rationale provided for the choice of model. The data were reanalysed using a fixed-effects model. This method was chosen following concerns that the number of studies included in the meta-analysis is too small for a precise estimate of the between studies variance, which might lead to an incorrect application of the random-effects model. The results of reanalyses using the fixed-effect model are provided here in S4 and S5 Files, and the results presented in Table 2 have been updated accordingly. The results of the reanalysis using the fixed-effect model support the results published in this meta-analysis.
2. The statistical reviewer raised concerns about the interpretation of the results and noted that a non-significant odds ratio (OR) is not the same as evidence for no association between specific polymorphisms and noise-induced hearing loss. In addition, the reviewer suggested that the direction of the effects should be discussed. The authors agree with the reviewer and clarify that the meta-analysis did not provide evidence to support an association between rs1043618, rs2075800, and rs2763979 polymorphisms and susceptibility to Noise-induced hearing loss (NIHL), but this should not be interpreted as evidence of absence of an association between these polymorphisms and NIHL. The authors also clarify that the OR>1 for rs1061581 and the OR<1 for rs2227956 suggest that Caucasians carrying allele G in rs1061581 or allele T in rs2227956 may be susceptible to NIHL.
3. Concerns were also raised that funnel plots and Egger’s tests were used to evaluate publication bias, but these methods are not recommended for meta-analyses involving <10 studies [3]. The authors agree with the reviewer that they did not consider the applicability of the funnel plot, Egger’s test, and the Begg’s method to this meta-analysis, and suggest that the results presented in these plots (Figure 7 and Table 3 in [1]) should be disregarded or interpreted with caution.
4. The statistical reviewer recommended that the authors carry out sensitivity analyses to evaluate the robustness of the presented results. Sensitivity analysis was carried out to examine the influence of each individual study on the pooled OR by deleting each study, one at the time, from the pooled analysis of the SNPs (rs1043618, rs1061581, and rs2227956). The results are presented in S5 File. The sensitivity analysis showed that the exclusion of Li, Y 2017 [12] greatly affected the pooled ORs, from 0.74(0.33–1.82) to 0.20(0.05–0.80) in the Homozygote model and from 0.90(0.35–1.80) to 0.22(0.06–0.88) in the Recessive model of rs2227956. The instability of the above results may be due to the small sample size of the individual studies in these two models. The sensitivity analysis did not demonstrate an obvious change in any of the other genetic models tested. These findings suggest that the results presented in this article are relatively robust for the genetic models of rs1043618, rs1061581, as well as for genetic models of rs2227956 except the homozygote and recessive models. Sensitivity analysis was not performed for rs2075800 and 2763979 due to the limited number of included studies for these polymorphisms.

A member of *PLOS ONE*’s Editorial Board and statistical reviewer confirmed that the revised analyses support the overall conclusions of the published article.

The full list of included articles, the full list of excluded articles with reasons for exclusions, and a spreadsheet of all data extracted from the primary literature as part of the meta-analysis have been uploaded as supplementary files S1, S6 and S7 Files. In addition, Table 1 has been updated to clarify the source of the control group for each study included in this meta-analysis.

Please see the correct Tables 1 and 2 here.

**Table 1:** Characteristics of studies included in the meta-analysis.

Author	Year	Country	Ethnicity studied	Case			Control			source of control	P-HWE	Quality
**rs1043618**				**GG**	**GC**	**CC**	**GG**	**GC**	**CC**			
Li, Y	2017	China	Chinese	124	117	45	130	125	31	PB	0.91	4/2/3
Chang, N C	2011	Taiwan	Chinese	8	18	1	153	139	30	PB	0.85	3/2/3
Konings, A	2009	Sweden	Caucasian	31	51	11	49	44	7	PB	0.49	3/2/3
Konings, A	2009	Poland	Caucasian	44	58	14	46	58	12	PB	0.31	3/2/3
Yang, M	2006	China	Chinese	37	43	13	35	48	18	PB	0.83	4/1/3
**rs1061581**				**AA**	**AG**	**GG**	**AA**	**AG**	**GG**			
Konings, A	2009	Sweden	Caucasian	24	55	13	44	45	11	PB	0.92	3/2/3
Konings, A	2009	Poland	Caucasian	37	61	18	43	56	15	PB	0.63	3/2/3
Yang, M	2006	China	Chinese	43	41	9	50	48	3	PB	**0.03**	4/1/3
**rs2075800**				**CC**	**TC**	**TT**	**CC**	**TC**	**TT**			
Li, Y	2017	China	Chinese	128	128	30	112	132	42	PB	0.76	4/2/3
Chang, N C	2011	Taiwan	Chinese	10	15	2	113	166	43	PB	0.14	3/2/3
**rs2227956**				**TT**	**TC**	**CC**	**TT**	**TC**	**CC**			
Li, Y	2017	China	Chinese	204	64	8	201	73	2	PB	0.09	4/2/3
Konings, A	2009	Sweden	Caucasian	64	27	0	54	39	5	PB	0.54	3/2/3
Konings, A	2009	Poland	Caucasian	95	22	1	81	32	4	PB	0.70	3/2/3
Yang, M	2006	China	Chinese	58	34	1	67	32	2	PB	0.41	4/1/3
**rs2763979**				**CC**	**TC**	**TT**	**CC**	**TC**	**TT**			
Li, Y	2017	China	Chinese	104	133	49	116	139	31	PB	0.26	4/2/3
Chang, N C	2011	China	Chinese	18	9	0	179	124	19	PB	0.68	3/2/3

P-HWE: P-value for the Hardy-Weinberg equilibrium; PB: population-based; Quality: Score of NOS scale

**Table 2:** Summary of pooled ORs in the meta-analysis.

	N	Allele model			Heterozygote model			Homozygote model			Dominant model			Recessive model		
		OR(95%CI)	I^2	P-H	OR(95%CI)	I^2	P-H	OR(95%CI)	I^2	P-H	OR(95%CI)	I^2	P-H	OR(95%CI)	I^2	P-H
**rs1043618**		C/G			GC/GG			CC/GG			CC+GC/GG			CC/CG+GG		
Overall	5	1.15(0.98–1.35)	19.7	0.29	1.15(0.91–1.45)	44	0.13	1.30(0.91–1.86)	10.6	0.35	1.19(0.95–1.48)	39.5	0.16	1.23(0.88–1.72)	5.1	0.38
*Ethnicity*																
Chinese	3	1.09(0.90–1.33)	18.2	0.29	1.06(0.80–1.42)	54.5	0.11	1.18(0.77–1.82)	29.4	0.24	1.10(0.84–1.44)	42.6	0.17	1.16(0.78–1.73)	45.4	0.16
Caucasian	2	1.28(0.96–1.69)	42.9	0.19	1.35(0.90–1.45)	44.7	0.18	1.63(0.84–3.18)	4	0.31	1.40(0.95–2.06)	52.2	0.15	1.40(0.75–2.63)	0	0.54
**rs1061581**		G/A			AG/AA			GG/AA			GG+AG/AA			GG/AG+AA		
Overall	3	**1.32(1.05–1.67)**	0	0.62	1.38(0.98–1.94)	43.7	0.17	**1.93(1.1–3.36)**	0	0.50	**1.45(1.05–2.02)**	27.7	0.25	1.50(0.90–2.50)	0	0.38
*Ethnicity*																
Chinese	1	1.27(0.82–1.97)	-	-	0.99(0.55–1.78)	-	-	3.49(0.89–13.71)	-	-	1.14(0.65–2.00)	-	-	3.50(0.92–13.35)	-	-
Caucasian	2	**1.34(1.02–1.77)**	0	0.33	**1.64(1.07–2.49)**	41.9	0.19	1.68(0.91–3.11)	0	0.49	**1.65(1.10–2.47)**	41	0.19	1.26(0.72–2.21)	0	0.87
**rs2075800**		T/C			CT/CC			TT/CC			TT+CT/CC			TT/CT+CC		
	2	0.81(0.65–1.02)	0	0.89	0.87(0.63–1.21)	0	0.69	0.61(0.37–1.01)	0	0.84	0.81(0.60–1.10)	0	0.75	0.66(0.41–1.06)	0	0.73
**rs2227956**		C/T			TC/TT			CC/TT			CC+TC/TT			CC/TC+TT		
Overall	4	0.83(0.66–1.03)	**63.7**	**0.04**	0.80(0.62–1.04)	27.1	0.25	0.74(0.33–1.67)	**62.4**	**0.05**	0.80(0.62–1.03)	50.7	0.11	0.80(0.35–1.80)	**60.3**	**0.06**
*Ethnicity*																
Chinese	2	1.06(0.80–1.41)	0	0.86	0.96(0.69–1.33)	0	0.33	2.33(0.70–7.73)	41.3	0.19	1.01(0.74–1.39)	0	0.52	2.33(0.71–7.67)	47.7	0.17
Caucasian	2	**0.54(0.37–0.78)**	0	0.90	**0.59(0.38–0.90)**	0	0.99	**0.13(0.02–0.76)**	0	0.58	**0.53(0.35–0.81)**	0	0.91	**0.16(0.03–0.89)**	0	0.60
**rs2763979**		T/C			CT/CC			TT/CC			TT+CT/CC			TT/CT+CC		
	2	1.15(0.92–1.44)	**71.7**	**0.06**	1.00(0.72–1.39)	0	0.40	1.56(0.95–2.56)	44.6	0.18	1.08(0.80–1.48)	50	0.16	1.55(0.98–2.46)	34.4	0.22

ORs: odds ratio of the association between NIHL susceptibility and each genetic model for each single nucleotide polymorphisms (SNP); P-H: P-value for heterogeneity. The following results have been updated following reanalysis using the fixed effects model only: rs2227956 C/T, rs2227956 CC/TT, rs2227956 CC/TC+II, and rs2763979 T/C.

## Supporting information

S1 File Overview of a total of 34 search records. (XLSX)

S2 File The Hardy-Weinberg equilibrium. (XLS)

S3 File Forest for rs2227956 and rs2763979 by fixed effect model. (PDF)

S4 File Sensitivity analysis. (DOCX)

S5 File Plot of sensitive analysis for rs1043618, rs1061581, and rs2227956. (PDF)

S6 File The full list of included articles. (XLSX)

S7 File The full list of excluded articles with reasons for each exclusion. (XLSX)
