# Supplementary figures and images for: Association between polymorphisms of heat-shock protein 70 genes and noise-induced hearing loss: A meta-analysis
Source: PLoS One. 2017 Nov 27;12(11):e0188539. doi: 10.1371/journal.pone.0188539 (PMC5703472; doi:10.1371/journal.pone.0188539)

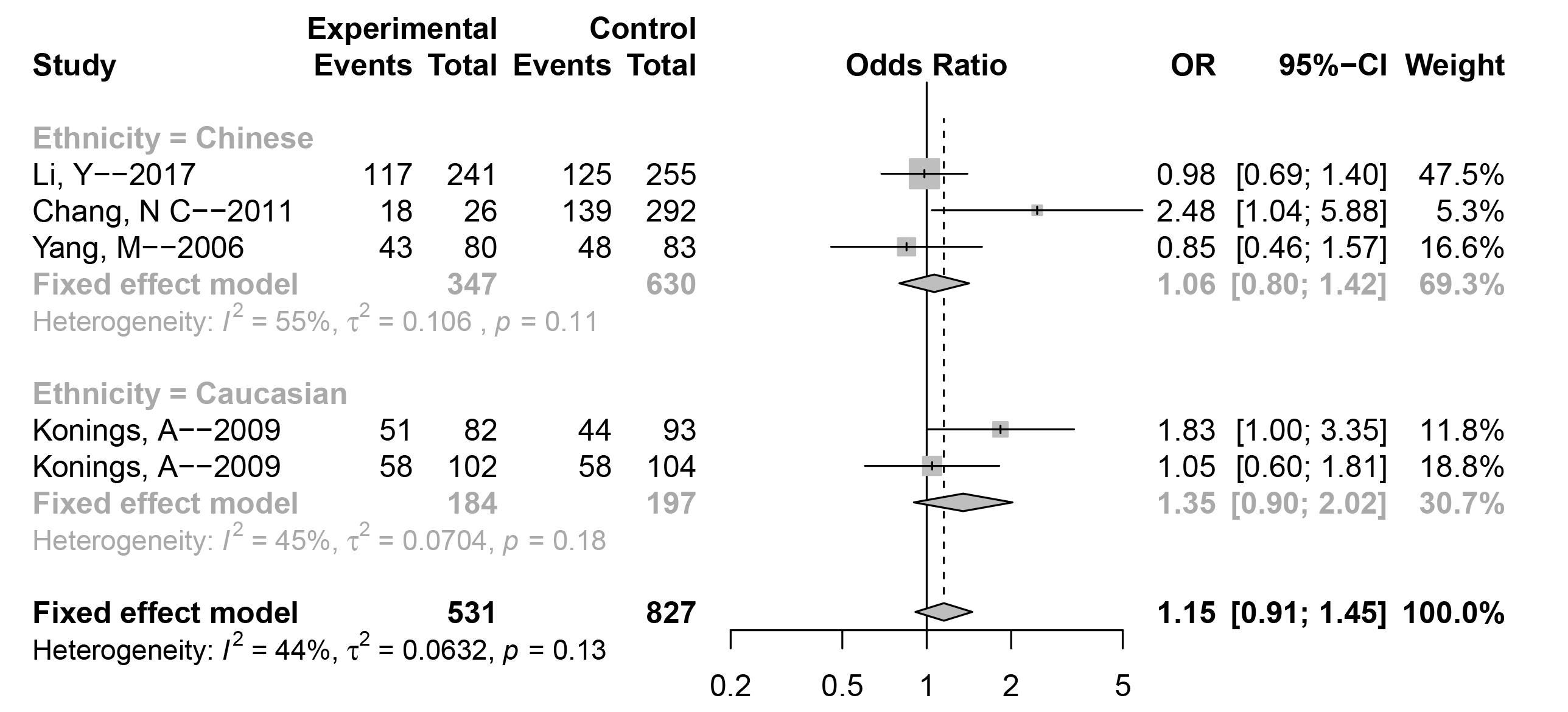

Supplement: S2 File — Forest plot (Figure A—Figure T). (ZIP). Figure A in S2 File: Forest plot of association between the rs1043618 polymorphism and NIHL (Heterozygote model: GC versus GG). Figure B in S2 File: Forest plot of association between the rs1043618 polymorphism and NIHL (Homozygote model: CC versus GG). Figure C in S2 File: Forest plot of association between the rs1043618 polymorphism and NIHL (Dominant model: CC+GC versus GG). Figure D in S2 File: Forest plot of association between the rs1043618 polymorphism and NIHL (Recessive model: CC versus CG+GG). Figure E in S2 File: Forest plot of association between the rs1061581 polymorphism and NIHL (Heterozygote model: AG versus AA). Figure F in S2 File: Forest plot of association between the rs1061581 polymorphism and NIHL (Homozygote model: GG versus AA). Figure G in S2 File: Forest plot of association between the rs1061581 polymorphism and NIHL (Dominant model: GG+AG versus AA). Figure H in S2 File: Forest plot of association between the rs1061581 polymorphism and NIHL (Recessive model: GG versus AG+AA). Figure I in S2 File: Forest plot of association between the rs2075800 polymorphism and NIHL (Heterozygote model: CT versus CC). Figure J in S2 File: Forest plot of association between the rs2075800 polymorphism and NIHL (Homozygote model: TT versus CC). Figure K in S2 File: Forest plot of association between the rs2075800 polymorphism and NIHL (Dominant model: TT+CT versus CC). Figure L in S2 File: Forest plot of association between the rs2075800 polymorphism and NIHL (Recessive model: TT versus CT+CC). Figure M in S2 File: Forest plot of association between the rs2227956 polymorphism and NIHL (Heterozygote model: TC versus TT). Figure N in S2 File: Forest plot of association between the rs2227956 polymorphism and NIHL (Homozygote model: CC versus TT). Figure O in S2 File: Forest plot of association between the rs2227956 polymorphism and NIHL (Dominant model: CC+TC versus TT). Figure P in S2 File: Forest plot of association betw [file pone.0188539.s002.zip › S2 File/Figure A.tif]

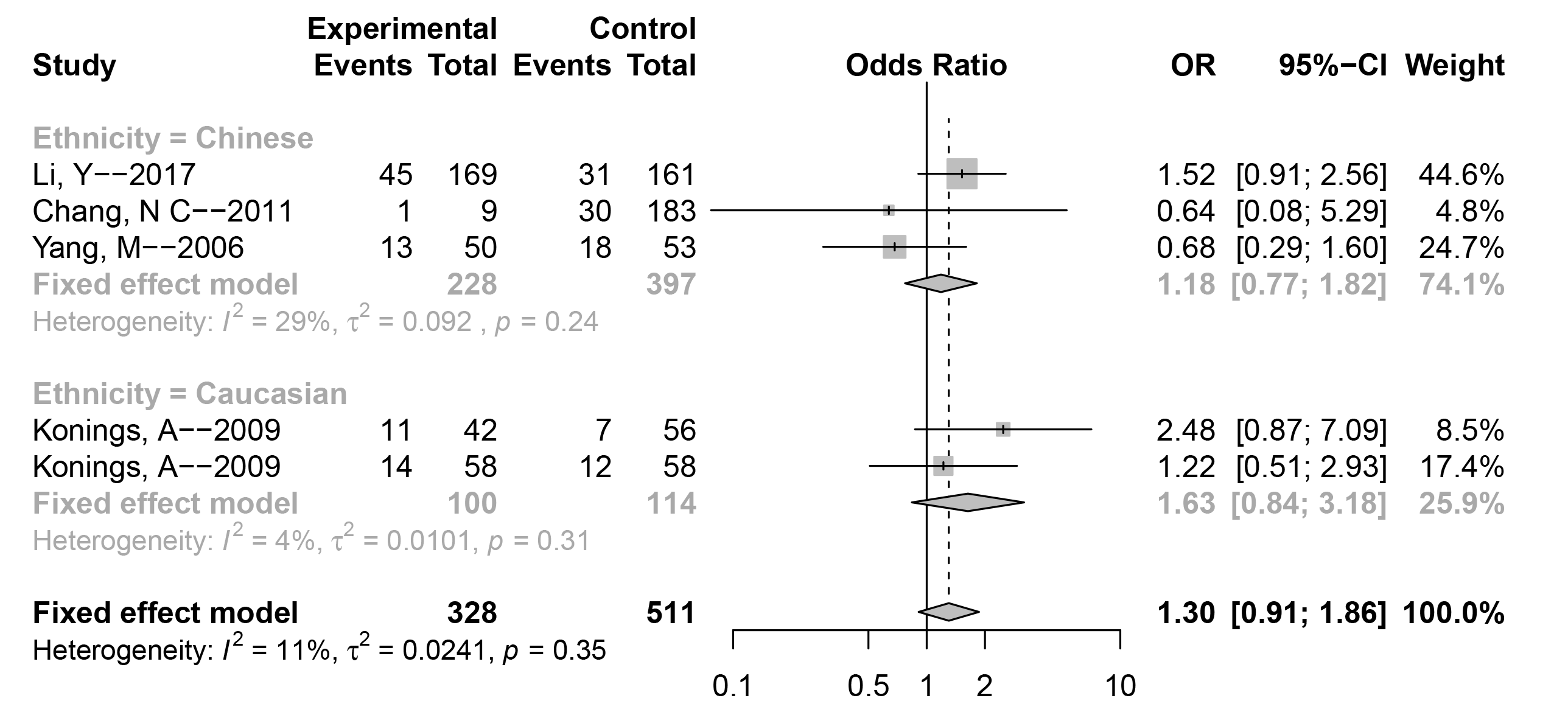

Supplement: S2 File — Forest plot (Figure A—Figure T). (ZIP). Figure A in S2 File: Forest plot of association between the rs1043618 polymorphism and NIHL (Heterozygote model: GC versus GG). Figure B in S2 File: Forest plot of association between the rs1043618 polymorphism and NIHL (Homozygote model: CC versus GG). Figure C in S2 File: Forest plot of association between the rs1043618 polymorphism and NIHL (Dominant model: CC+GC versus GG). Figure D in S2 File: Forest plot of association between the rs1043618 polymorphism and NIHL (Recessive model: CC versus CG+GG). Figure E in S2 File: Forest plot of association between the rs1061581 polymorphism and NIHL (Heterozygote model: AG versus AA). Figure F in S2 File: Forest plot of association between the rs1061581 polymorphism and NIHL (Homozygote model: GG versus AA). Figure G in S2 File: Forest plot of association between the rs1061581 polymorphism and NIHL (Dominant model: GG+AG versus AA). Figure H in S2 File: Forest plot of association between the rs1061581 polymorphism and NIHL (Recessive model: GG versus AG+AA). Figure I in S2 File: Forest plot of association between the rs2075800 polymorphism and NIHL (Heterozygote model: CT versus CC). Figure J in S2 File: Forest plot of association between the rs2075800 polymorphism and NIHL (Homozygote model: TT versus CC). Figure K in S2 File: Forest plot of association between the rs2075800 polymorphism and NIHL (Dominant model: TT+CT versus CC). Figure L in S2 File: Forest plot of association between the rs2075800 polymorphism and NIHL (Recessive model: TT versus CT+CC). Figure M in S2 File: Forest plot of association between the rs2227956 polymorphism and NIHL (Heterozygote model: TC versus TT). Figure N in S2 File: Forest plot of association between the rs2227956 polymorphism and NIHL (Homozygote model: CC versus TT). Figure O in S2 File: Forest plot of association between the rs2227956 polymorphism and NIHL (Dominant model: CC+TC versus TT). Figure P in S2 File: Forest plot of association betw [file pone.0188539.s002.zip › S2 File/Figure B.tif]

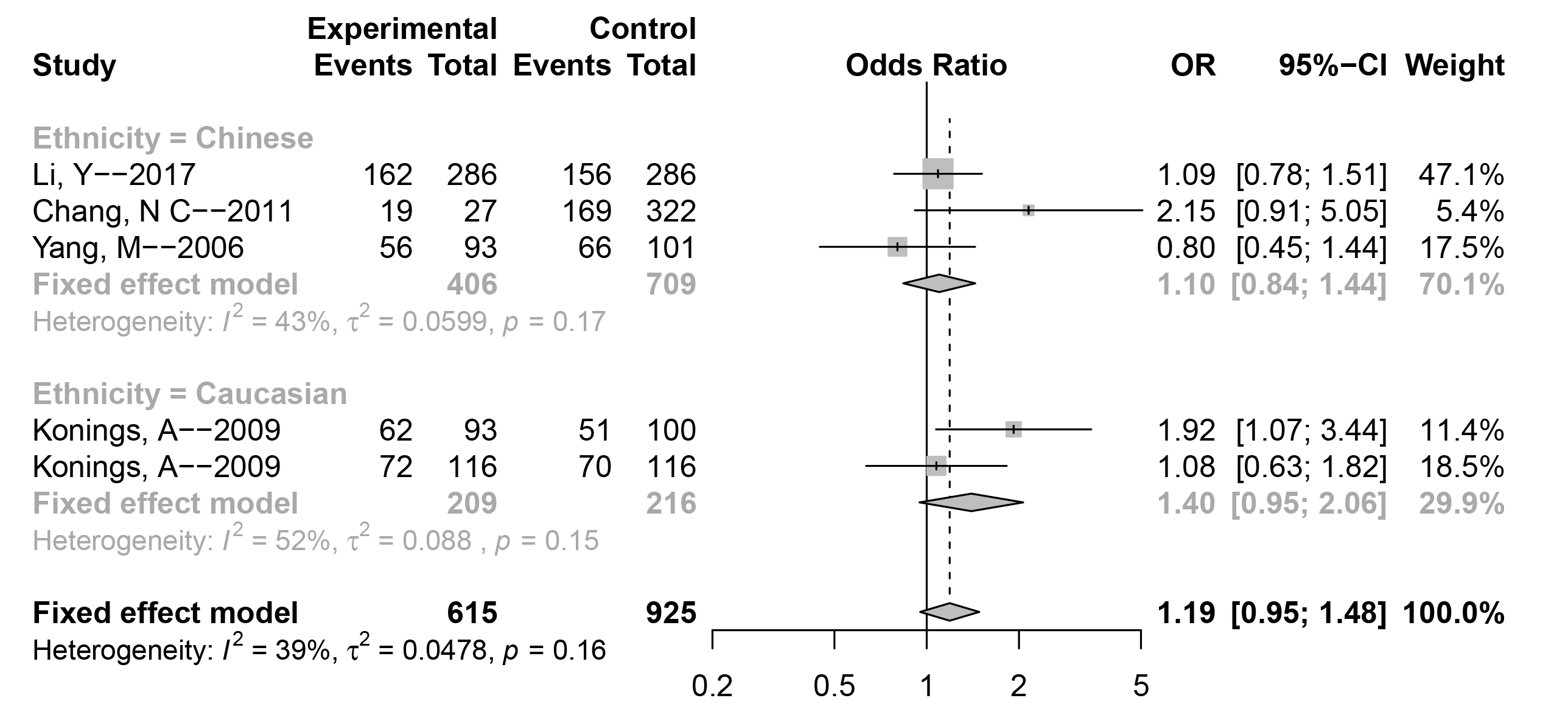

Supplement: S2 File — Forest plot (Figure A—Figure T). (ZIP). Figure A in S2 File: Forest plot of association between the rs1043618 polymorphism and NIHL (Heterozygote model: GC versus GG). Figure B in S2 File: Forest plot of association between the rs1043618 polymorphism and NIHL (Homozygote model: CC versus GG). Figure C in S2 File: Forest plot of association between the rs1043618 polymorphism and NIHL (Dominant model: CC+GC versus GG). Figure D in S2 File: Forest plot of association between the rs1043618 polymorphism and NIHL (Recessive model: CC versus CG+GG). Figure E in S2 File: Forest plot of association between the rs1061581 polymorphism and NIHL (Heterozygote model: AG versus AA). Figure F in S2 File: Forest plot of association between the rs1061581 polymorphism and NIHL (Homozygote model: GG versus AA). Figure G in S2 File: Forest plot of association between the rs1061581 polymorphism and NIHL (Dominant model: GG+AG versus AA). Figure H in S2 File: Forest plot of association between the rs1061581 polymorphism and NIHL (Recessive model: GG versus AG+AA). Figure I in S2 File: Forest plot of association between the rs2075800 polymorphism and NIHL (Heterozygote model: CT versus CC). Figure J in S2 File: Forest plot of association between the rs2075800 polymorphism and NIHL (Homozygote model: TT versus CC). Figure K in S2 File: Forest plot of association between the rs2075800 polymorphism and NIHL (Dominant model: TT+CT versus CC). Figure L in S2 File: Forest plot of association between the rs2075800 polymorphism and NIHL (Recessive model: TT versus CT+CC). Figure M in S2 File: Forest plot of association between the rs2227956 polymorphism and NIHL (Heterozygote model: TC versus TT). Figure N in S2 File: Forest plot of association between the rs2227956 polymorphism and NIHL (Homozygote model: CC versus TT). Figure O in S2 File: Forest plot of association between the rs2227956 polymorphism and NIHL (Dominant model: CC+TC versus TT). Figure P in S2 File: Forest plot of association betw [file pone.0188539.s002.zip › S2 File/Figure C.tif]

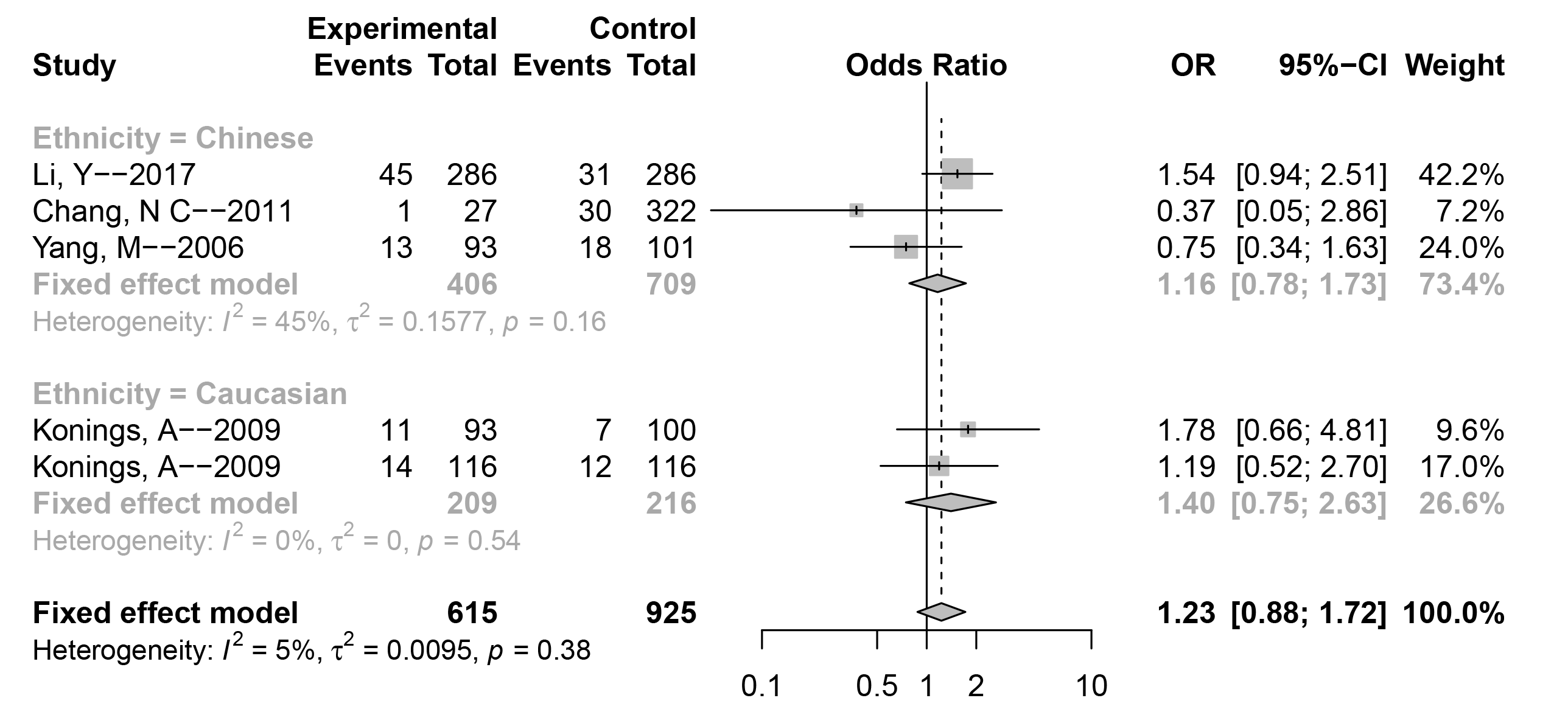

Supplement: S2 File — Forest plot (Figure A—Figure T). (ZIP). Figure A in S2 File: Forest plot of association between the rs1043618 polymorphism and NIHL (Heterozygote model: GC versus GG). Figure B in S2 File: Forest plot of association between the rs1043618 polymorphism and NIHL (Homozygote model: CC versus GG). Figure C in S2 File: Forest plot of association between the rs1043618 polymorphism and NIHL (Dominant model: CC+GC versus GG). Figure D in S2 File: Forest plot of association between the rs1043618 polymorphism and NIHL (Recessive model: CC versus CG+GG). Figure E in S2 File: Forest plot of association between the rs1061581 polymorphism and NIHL (Heterozygote model: AG versus AA). Figure F in S2 File: Forest plot of association between the rs1061581 polymorphism and NIHL (Homozygote model: GG versus AA). Figure G in S2 File: Forest plot of association between the rs1061581 polymorphism and NIHL (Dominant model: GG+AG versus AA). Figure H in S2 File: Forest plot of association between the rs1061581 polymorphism and NIHL (Recessive model: GG versus AG+AA). Figure I in S2 File: Forest plot of association between the rs2075800 polymorphism and NIHL (Heterozygote model: CT versus CC). Figure J in S2 File: Forest plot of association between the rs2075800 polymorphism and NIHL (Homozygote model: TT versus CC). Figure K in S2 File: Forest plot of association between the rs2075800 polymorphism and NIHL (Dominant model: TT+CT versus CC). Figure L in S2 File: Forest plot of association between the rs2075800 polymorphism and NIHL (Recessive model: TT versus CT+CC). Figure M in S2 File: Forest plot of association between the rs2227956 polymorphism and NIHL (Heterozygote model: TC versus TT). Figure N in S2 File: Forest plot of association between the rs2227956 polymorphism and NIHL (Homozygote model: CC versus TT). Figure O in S2 File: Forest plot of association between the rs2227956 polymorphism and NIHL (Dominant model: CC+TC versus TT). Figure P in S2 File: Forest plot of association betw [file pone.0188539.s002.zip › S2 File/Figure D.tif]

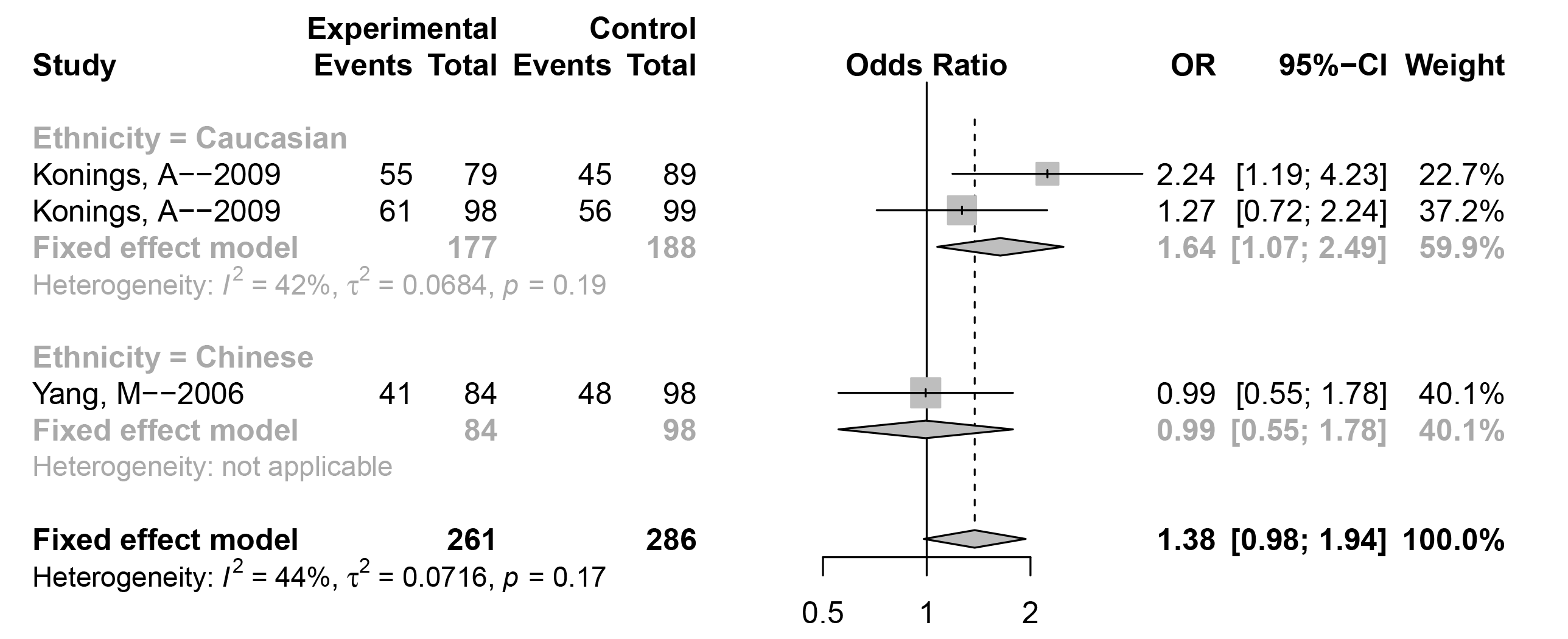

Supplement: S2 File — Forest plot (Figure A—Figure T). (ZIP). Figure A in S2 File: Forest plot of association between the rs1043618 polymorphism and NIHL (Heterozygote model: GC versus GG). Figure B in S2 File: Forest plot of association between the rs1043618 polymorphism and NIHL (Homozygote model: CC versus GG). Figure C in S2 File: Forest plot of association between the rs1043618 polymorphism and NIHL (Dominant model: CC+GC versus GG). Figure D in S2 File: Forest plot of association between the rs1043618 polymorphism and NIHL (Recessive model: CC versus CG+GG). Figure E in S2 File: Forest plot of association between the rs1061581 polymorphism and NIHL (Heterozygote model: AG versus AA). Figure F in S2 File: Forest plot of association between the rs1061581 polymorphism and NIHL (Homozygote model: GG versus AA). Figure G in S2 File: Forest plot of association between the rs1061581 polymorphism and NIHL (Dominant model: GG+AG versus AA). Figure H in S2 File: Forest plot of association between the rs1061581 polymorphism and NIHL (Recessive model: GG versus AG+AA). Figure I in S2 File: Forest plot of association between the rs2075800 polymorphism and NIHL (Heterozygote model: CT versus CC). Figure J in S2 File: Forest plot of association between the rs2075800 polymorphism and NIHL (Homozygote model: TT versus CC). Figure K in S2 File: Forest plot of association between the rs2075800 polymorphism and NIHL (Dominant model: TT+CT versus CC). Figure L in S2 File: Forest plot of association between the rs2075800 polymorphism and NIHL (Recessive model: TT versus CT+CC). Figure M in S2 File: Forest plot of association between the rs2227956 polymorphism and NIHL (Heterozygote model: TC versus TT). Figure N in S2 File: Forest plot of association between the rs2227956 polymorphism and NIHL (Homozygote model: CC versus TT). Figure O in S2 File: Forest plot of association between the rs2227956 polymorphism and NIHL (Dominant model: CC+TC versus TT). Figure P in S2 File: Forest plot of association betw [file pone.0188539.s002.zip › S2 File/Figure E.tif]

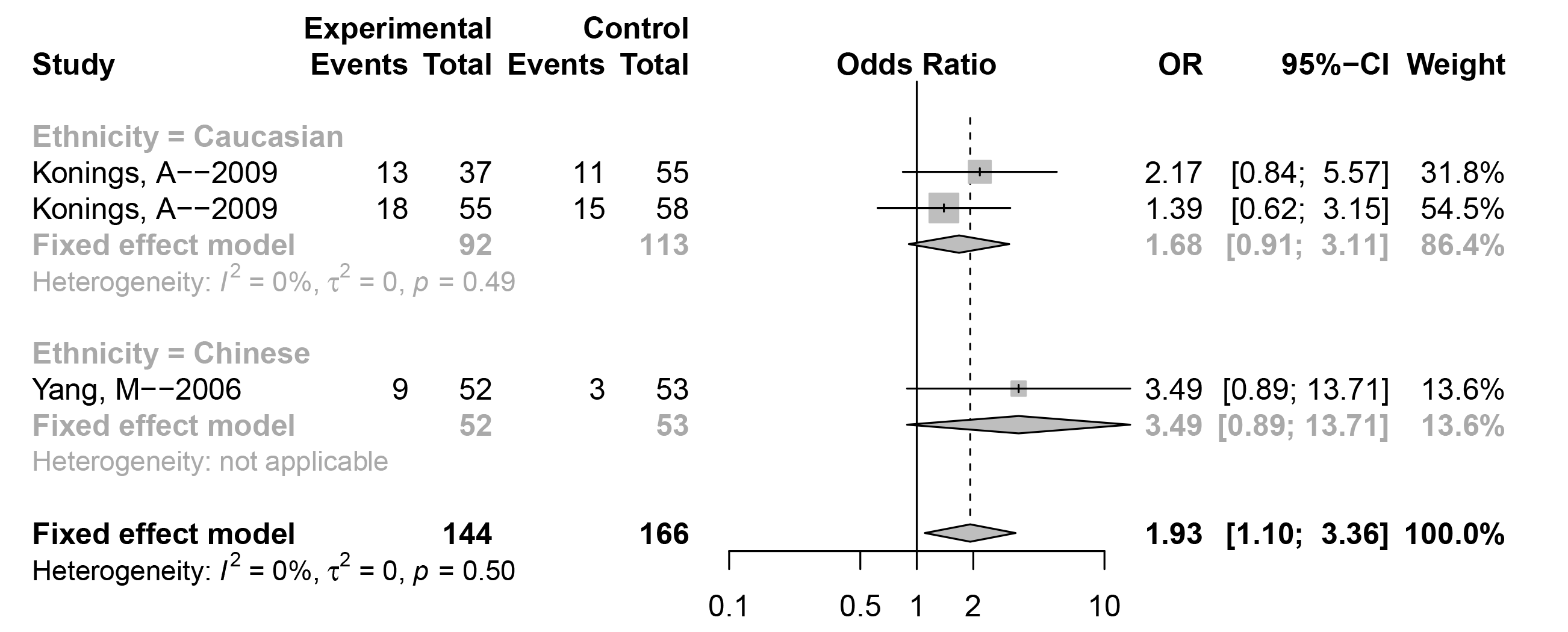

Supplement: S2 File — Forest plot (Figure A—Figure T). (ZIP). Figure A in S2 File: Forest plot of association between the rs1043618 polymorphism and NIHL (Heterozygote model: GC versus GG). Figure B in S2 File: Forest plot of association between the rs1043618 polymorphism and NIHL (Homozygote model: CC versus GG). Figure C in S2 File: Forest plot of association between the rs1043618 polymorphism and NIHL (Dominant model: CC+GC versus GG). Figure D in S2 File: Forest plot of association between the rs1043618 polymorphism and NIHL (Recessive model: CC versus CG+GG). Figure E in S2 File: Forest plot of association between the rs1061581 polymorphism and NIHL (Heterozygote model: AG versus AA). Figure F in S2 File: Forest plot of association between the rs1061581 polymorphism and NIHL (Homozygote model: GG versus AA). Figure G in S2 File: Forest plot of association between the rs1061581 polymorphism and NIHL (Dominant model: GG+AG versus AA). Figure H in S2 File: Forest plot of association between the rs1061581 polymorphism and NIHL (Recessive model: GG versus AG+AA). Figure I in S2 File: Forest plot of association between the rs2075800 polymorphism and NIHL (Heterozygote model: CT versus CC). Figure J in S2 File: Forest plot of association between the rs2075800 polymorphism and NIHL (Homozygote model: TT versus CC). Figure K in S2 File: Forest plot of association between the rs2075800 polymorphism and NIHL (Dominant model: TT+CT versus CC). Figure L in S2 File: Forest plot of association between the rs2075800 polymorphism and NIHL (Recessive model: TT versus CT+CC). Figure M in S2 File: Forest plot of association between the rs2227956 polymorphism and NIHL (Heterozygote model: TC versus TT). Figure N in S2 File: Forest plot of association between the rs2227956 polymorphism and NIHL (Homozygote model: CC versus TT). Figure O in S2 File: Forest plot of association between the rs2227956 polymorphism and NIHL (Dominant model: CC+TC versus TT). Figure P in S2 File: Forest plot of association betw [file pone.0188539.s002.zip › S2 File/Figure F.tif]

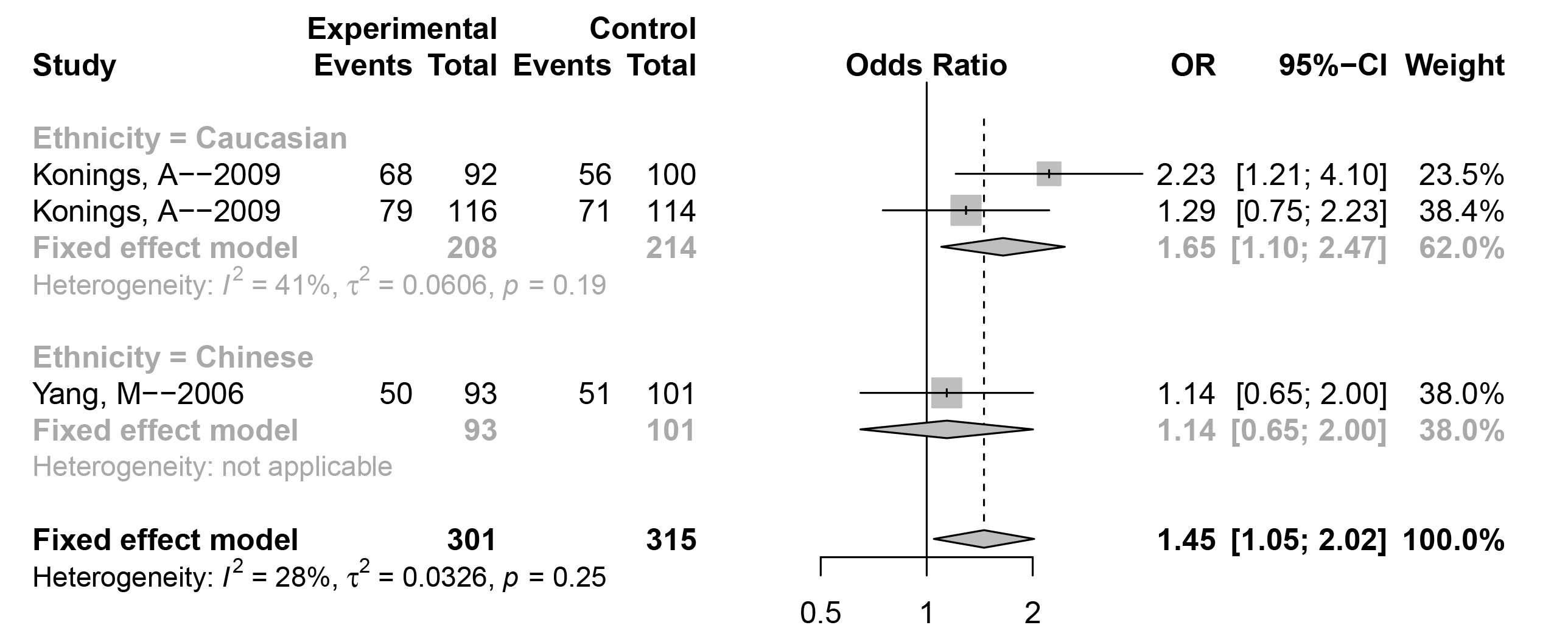

Supplement: S2 File — Forest plot (Figure A—Figure T). (ZIP). Figure A in S2 File: Forest plot of association between the rs1043618 polymorphism and NIHL (Heterozygote model: GC versus GG). Figure B in S2 File: Forest plot of association between the rs1043618 polymorphism and NIHL (Homozygote model: CC versus GG). Figure C in S2 File: Forest plot of association between the rs1043618 polymorphism and NIHL (Dominant model: CC+GC versus GG). Figure D in S2 File: Forest plot of association between the rs1043618 polymorphism and NIHL (Recessive model: CC versus CG+GG). Figure E in S2 File: Forest plot of association between the rs1061581 polymorphism and NIHL (Heterozygote model: AG versus AA). Figure F in S2 File: Forest plot of association between the rs1061581 polymorphism and NIHL (Homozygote model: GG versus AA). Figure G in S2 File: Forest plot of association between the rs1061581 polymorphism and NIHL (Dominant model: GG+AG versus AA). Figure H in S2 File: Forest plot of association between the rs1061581 polymorphism and NIHL (Recessive model: GG versus AG+AA). Figure I in S2 File: Forest plot of association between the rs2075800 polymorphism and NIHL (Heterozygote model: CT versus CC). Figure J in S2 File: Forest plot of association between the rs2075800 polymorphism and NIHL (Homozygote model: TT versus CC). Figure K in S2 File: Forest plot of association between the rs2075800 polymorphism and NIHL (Dominant model: TT+CT versus CC). Figure L in S2 File: Forest plot of association between the rs2075800 polymorphism and NIHL (Recessive model: TT versus CT+CC). Figure M in S2 File: Forest plot of association between the rs2227956 polymorphism and NIHL (Heterozygote model: TC versus TT). Figure N in S2 File: Forest plot of association between the rs2227956 polymorphism and NIHL (Homozygote model: CC versus TT). Figure O in S2 File: Forest plot of association between the rs2227956 polymorphism and NIHL (Dominant model: CC+TC versus TT). Figure P in S2 File: Forest plot of association betw [file pone.0188539.s002.zip › S2 File/Figure G.tif]

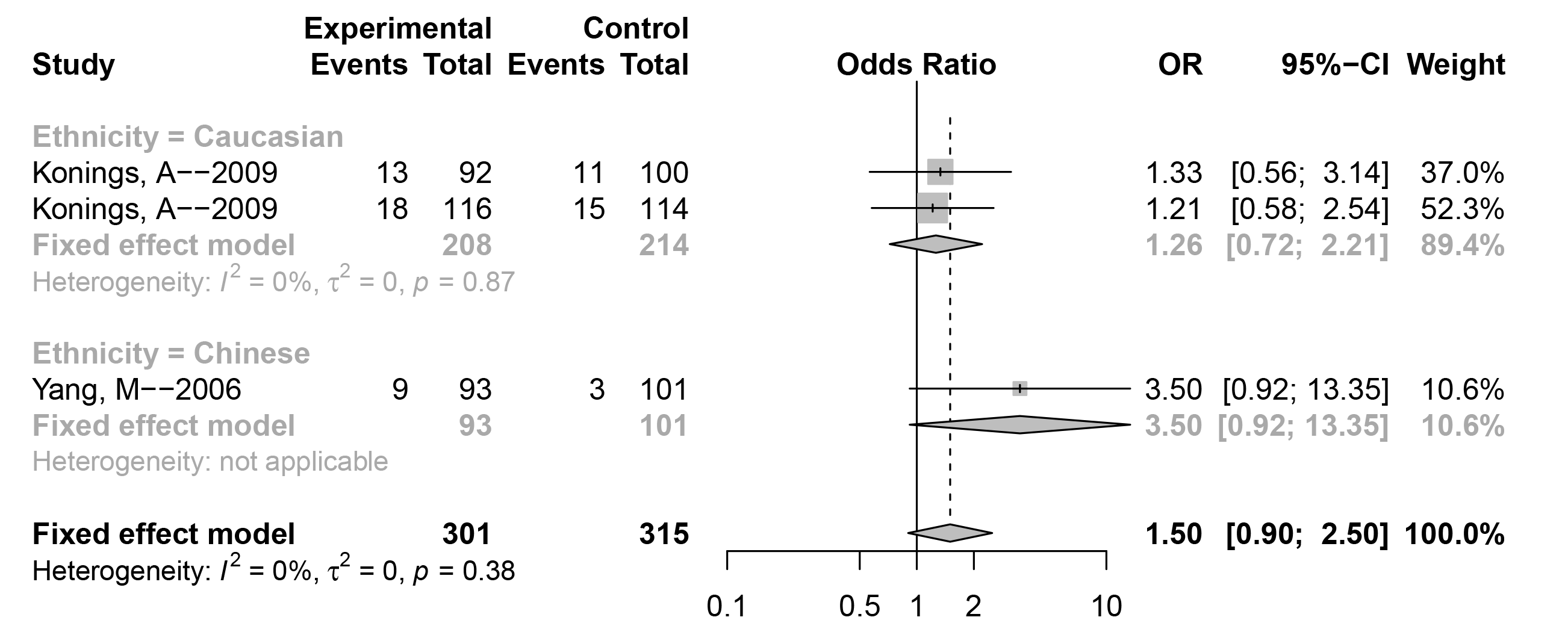

Supplement: S2 File — Forest plot (Figure A—Figure T). (ZIP). Figure A in S2 File: Forest plot of association between the rs1043618 polymorphism and NIHL (Heterozygote model: GC versus GG). Figure B in S2 File: Forest plot of association between the rs1043618 polymorphism and NIHL (Homozygote model: CC versus GG). Figure C in S2 File: Forest plot of association between the rs1043618 polymorphism and NIHL (Dominant model: CC+GC versus GG). Figure D in S2 File: Forest plot of association between the rs1043618 polymorphism and NIHL (Recessive model: CC versus CG+GG). Figure E in S2 File: Forest plot of association between the rs1061581 polymorphism and NIHL (Heterozygote model: AG versus AA). Figure F in S2 File: Forest plot of association between the rs1061581 polymorphism and NIHL (Homozygote model: GG versus AA). Figure G in S2 File: Forest plot of association between the rs1061581 polymorphism and NIHL (Dominant model: GG+AG versus AA). Figure H in S2 File: Forest plot of association between the rs1061581 polymorphism and NIHL (Recessive model: GG versus AG+AA). Figure I in S2 File: Forest plot of association between the rs2075800 polymorphism and NIHL (Heterozygote model: CT versus CC). Figure J in S2 File: Forest plot of association between the rs2075800 polymorphism and NIHL (Homozygote model: TT versus CC). Figure K in S2 File: Forest plot of association between the rs2075800 polymorphism and NIHL (Dominant model: TT+CT versus CC). Figure L in S2 File: Forest plot of association between the rs2075800 polymorphism and NIHL (Recessive model: TT versus CT+CC). Figure M in S2 File: Forest plot of association between the rs2227956 polymorphism and NIHL (Heterozygote model: TC versus TT). Figure N in S2 File: Forest plot of association between the rs2227956 polymorphism and NIHL (Homozygote model: CC versus TT). Figure O in S2 File: Forest plot of association between the rs2227956 polymorphism and NIHL (Dominant model: CC+TC versus TT). Figure P in S2 File: Forest plot of association betw [file pone.0188539.s002.zip › S2 File/Figure H.tif]

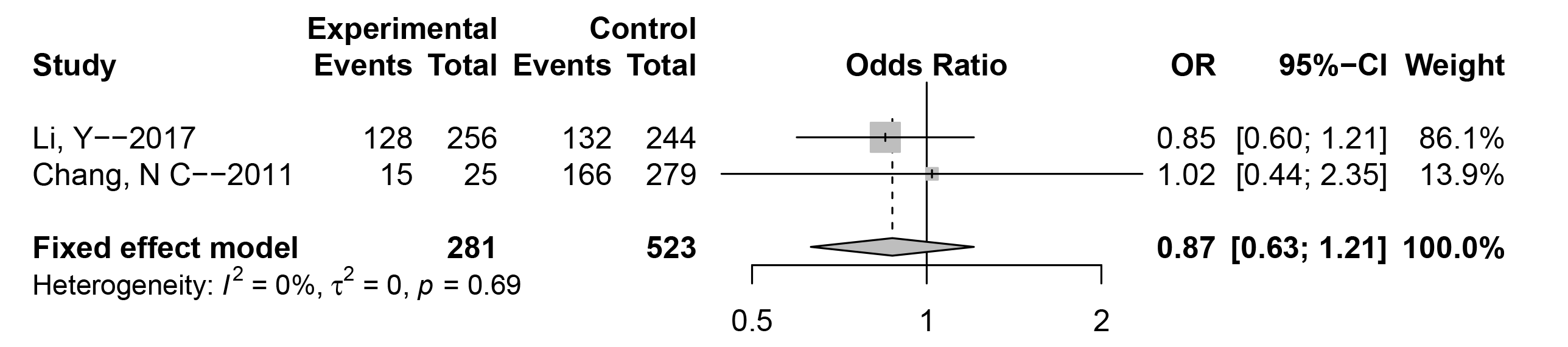

Supplement: S2 File — Forest plot (Figure A—Figure T). (ZIP). Figure A in S2 File: Forest plot of association between the rs1043618 polymorphism and NIHL (Heterozygote model: GC versus GG). Figure B in S2 File: Forest plot of association between the rs1043618 polymorphism and NIHL (Homozygote model: CC versus GG). Figure C in S2 File: Forest plot of association between the rs1043618 polymorphism and NIHL (Dominant model: CC+GC versus GG). Figure D in S2 File: Forest plot of association between the rs1043618 polymorphism and NIHL (Recessive model: CC versus CG+GG). Figure E in S2 File: Forest plot of association between the rs1061581 polymorphism and NIHL (Heterozygote model: AG versus AA). Figure F in S2 File: Forest plot of association between the rs1061581 polymorphism and NIHL (Homozygote model: GG versus AA). Figure G in S2 File: Forest plot of association between the rs1061581 polymorphism and NIHL (Dominant model: GG+AG versus AA). Figure H in S2 File: Forest plot of association between the rs1061581 polymorphism and NIHL (Recessive model: GG versus AG+AA). Figure I in S2 File: Forest plot of association between the rs2075800 polymorphism and NIHL (Heterozygote model: CT versus CC). Figure J in S2 File: Forest plot of association between the rs2075800 polymorphism and NIHL (Homozygote model: TT versus CC). Figure K in S2 File: Forest plot of association between the rs2075800 polymorphism and NIHL (Dominant model: TT+CT versus CC). Figure L in S2 File: Forest plot of association between the rs2075800 polymorphism and NIHL (Recessive model: TT versus CT+CC). Figure M in S2 File: Forest plot of association between the rs2227956 polymorphism and NIHL (Heterozygote model: TC versus TT). Figure N in S2 File: Forest plot of association between the rs2227956 polymorphism and NIHL (Homozygote model: CC versus TT). Figure O in S2 File: Forest plot of association between the rs2227956 polymorphism and NIHL (Dominant model: CC+TC versus TT). Figure P in S2 File: Forest plot of association betw [file pone.0188539.s002.zip › S2 File/Figure I.tif]

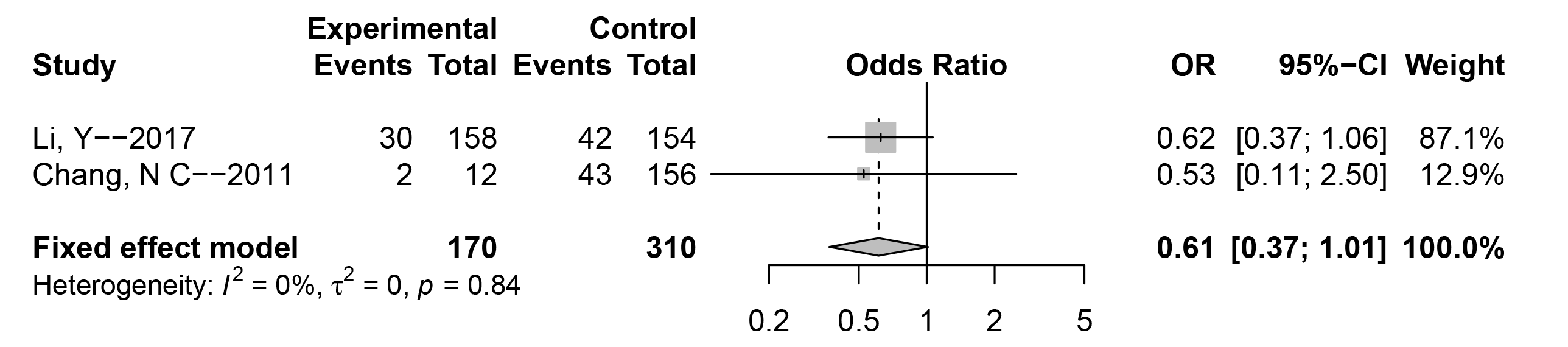

Supplement: S2 File — Forest plot (Figure A—Figure T). (ZIP). Figure A in S2 File: Forest plot of association between the rs1043618 polymorphism and NIHL (Heterozygote model: GC versus GG). Figure B in S2 File: Forest plot of association between the rs1043618 polymorphism and NIHL (Homozygote model: CC versus GG). Figure C in S2 File: Forest plot of association between the rs1043618 polymorphism and NIHL (Dominant model: CC+GC versus GG). Figure D in S2 File: Forest plot of association between the rs1043618 polymorphism and NIHL (Recessive model: CC versus CG+GG). Figure E in S2 File: Forest plot of association between the rs1061581 polymorphism and NIHL (Heterozygote model: AG versus AA). Figure F in S2 File: Forest plot of association between the rs1061581 polymorphism and NIHL (Homozygote model: GG versus AA). Figure G in S2 File: Forest plot of association between the rs1061581 polymorphism and NIHL (Dominant model: GG+AG versus AA). Figure H in S2 File: Forest plot of association between the rs1061581 polymorphism and NIHL (Recessive model: GG versus AG+AA). Figure I in S2 File: Forest plot of association between the rs2075800 polymorphism and NIHL (Heterozygote model: CT versus CC). Figure J in S2 File: Forest plot of association between the rs2075800 polymorphism and NIHL (Homozygote model: TT versus CC). Figure K in S2 File: Forest plot of association between the rs2075800 polymorphism and NIHL (Dominant model: TT+CT versus CC). Figure L in S2 File: Forest plot of association between the rs2075800 polymorphism and NIHL (Recessive model: TT versus CT+CC). Figure M in S2 File: Forest plot of association between the rs2227956 polymorphism and NIHL (Heterozygote model: TC versus TT). Figure N in S2 File: Forest plot of association between the rs2227956 polymorphism and NIHL (Homozygote model: CC versus TT). Figure O in S2 File: Forest plot of association between the rs2227956 polymorphism and NIHL (Dominant model: CC+TC versus TT). Figure P in S2 File: Forest plot of association betw [file pone.0188539.s002.zip › S2 File/Figure J.tif]

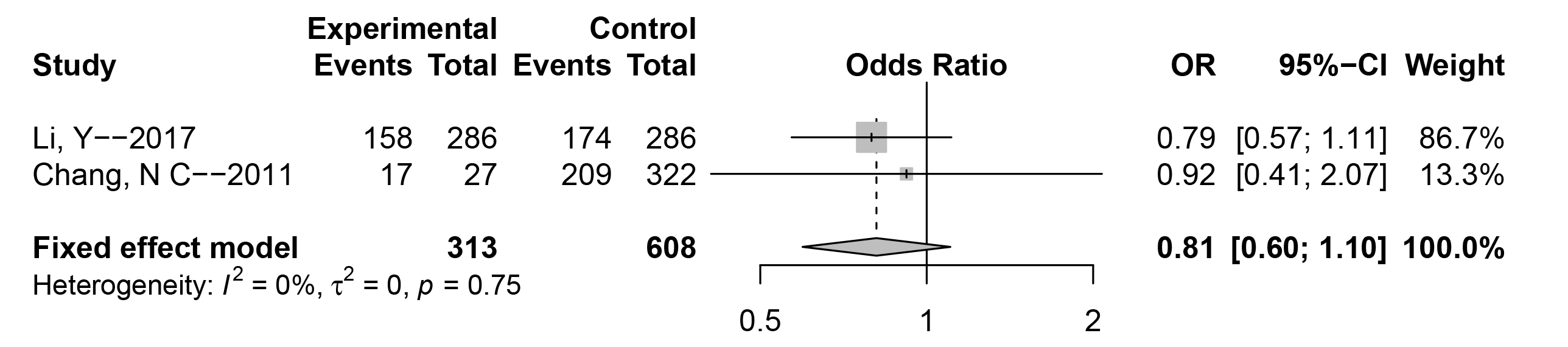

Supplement: S2 File — Forest plot (Figure A—Figure T). (ZIP). Figure A in S2 File: Forest plot of association between the rs1043618 polymorphism and NIHL (Heterozygote model: GC versus GG). Figure B in S2 File: Forest plot of association between the rs1043618 polymorphism and NIHL (Homozygote model: CC versus GG). Figure C in S2 File: Forest plot of association between the rs1043618 polymorphism and NIHL (Dominant model: CC+GC versus GG). Figure D in S2 File: Forest plot of association between the rs1043618 polymorphism and NIHL (Recessive model: CC versus CG+GG). Figure E in S2 File: Forest plot of association between the rs1061581 polymorphism and NIHL (Heterozygote model: AG versus AA). Figure F in S2 File: Forest plot of association between the rs1061581 polymorphism and NIHL (Homozygote model: GG versus AA). Figure G in S2 File: Forest plot of association between the rs1061581 polymorphism and NIHL (Dominant model: GG+AG versus AA). Figure H in S2 File: Forest plot of association between the rs1061581 polymorphism and NIHL (Recessive model: GG versus AG+AA). Figure I in S2 File: Forest plot of association between the rs2075800 polymorphism and NIHL (Heterozygote model: CT versus CC). Figure J in S2 File: Forest plot of association between the rs2075800 polymorphism and NIHL (Homozygote model: TT versus CC). Figure K in S2 File: Forest plot of association between the rs2075800 polymorphism and NIHL (Dominant model: TT+CT versus CC). Figure L in S2 File: Forest plot of association between the rs2075800 polymorphism and NIHL (Recessive model: TT versus CT+CC). Figure M in S2 File: Forest plot of association between the rs2227956 polymorphism and NIHL (Heterozygote model: TC versus TT). Figure N in S2 File: Forest plot of association between the rs2227956 polymorphism and NIHL (Homozygote model: CC versus TT). Figure O in S2 File: Forest plot of association between the rs2227956 polymorphism and NIHL (Dominant model: CC+TC versus TT). Figure P in S2 File: Forest plot of association betw [file pone.0188539.s002.zip › S2 File/Figure K.tif]

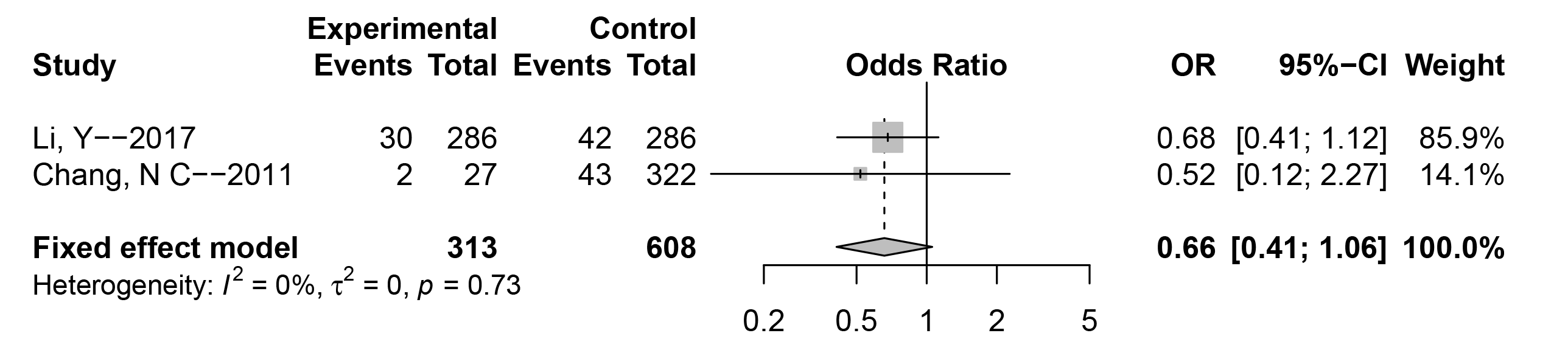

Supplement: S2 File — Forest plot (Figure A—Figure T). (ZIP). Figure A in S2 File: Forest plot of association between the rs1043618 polymorphism and NIHL (Heterozygote model: GC versus GG). Figure B in S2 File: Forest plot of association between the rs1043618 polymorphism and NIHL (Homozygote model: CC versus GG). Figure C in S2 File: Forest plot of association between the rs1043618 polymorphism and NIHL (Dominant model: CC+GC versus GG). Figure D in S2 File: Forest plot of association between the rs1043618 polymorphism and NIHL (Recessive model: CC versus CG+GG). Figure E in S2 File: Forest plot of association between the rs1061581 polymorphism and NIHL (Heterozygote model: AG versus AA). Figure F in S2 File: Forest plot of association between the rs1061581 polymorphism and NIHL (Homozygote model: GG versus AA). Figure G in S2 File: Forest plot of association between the rs1061581 polymorphism and NIHL (Dominant model: GG+AG versus AA). Figure H in S2 File: Forest plot of association between the rs1061581 polymorphism and NIHL (Recessive model: GG versus AG+AA). Figure I in S2 File: Forest plot of association between the rs2075800 polymorphism and NIHL (Heterozygote model: CT versus CC). Figure J in S2 File: Forest plot of association between the rs2075800 polymorphism and NIHL (Homozygote model: TT versus CC). Figure K in S2 File: Forest plot of association between the rs2075800 polymorphism and NIHL (Dominant model: TT+CT versus CC). Figure L in S2 File: Forest plot of association between the rs2075800 polymorphism and NIHL (Recessive model: TT versus CT+CC). Figure M in S2 File: Forest plot of association between the rs2227956 polymorphism and NIHL (Heterozygote model: TC versus TT). Figure N in S2 File: Forest plot of association between the rs2227956 polymorphism and NIHL (Homozygote model: CC versus TT). Figure O in S2 File: Forest plot of association between the rs2227956 polymorphism and NIHL (Dominant model: CC+TC versus TT). Figure P in S2 File: Forest plot of association betw [file pone.0188539.s002.zip › S2 File/Figure L.tif]

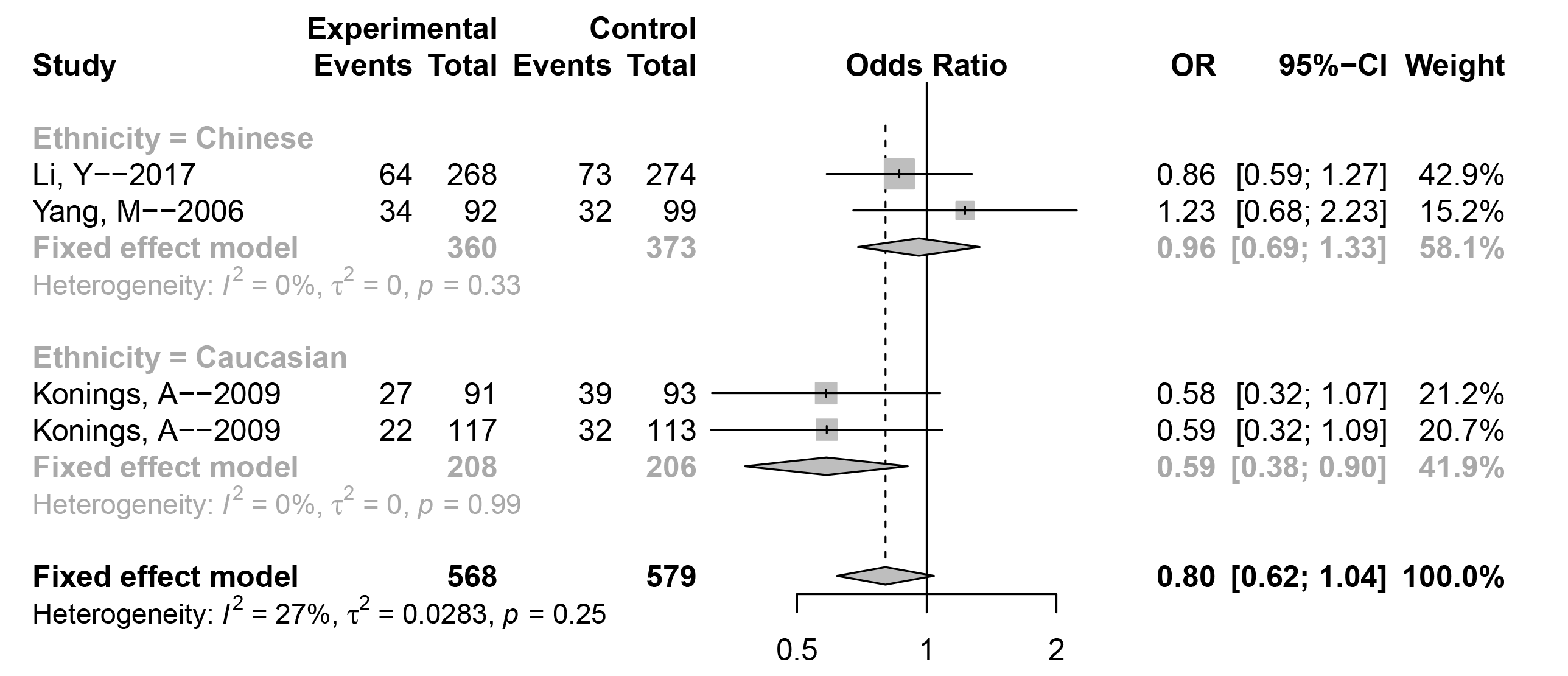

Supplement: S2 File — Forest plot (Figure A—Figure T). (ZIP). Figure A in S2 File: Forest plot of association between the rs1043618 polymorphism and NIHL (Heterozygote model: GC versus GG). Figure B in S2 File: Forest plot of association between the rs1043618 polymorphism and NIHL (Homozygote model: CC versus GG). Figure C in S2 File: Forest plot of association between the rs1043618 polymorphism and NIHL (Dominant model: CC+GC versus GG). Figure D in S2 File: Forest plot of association between the rs1043618 polymorphism and NIHL (Recessive model: CC versus CG+GG). Figure E in S2 File: Forest plot of association between the rs1061581 polymorphism and NIHL (Heterozygote model: AG versus AA). Figure F in S2 File: Forest plot of association between the rs1061581 polymorphism and NIHL (Homozygote model: GG versus AA). Figure G in S2 File: Forest plot of association between the rs1061581 polymorphism and NIHL (Dominant model: GG+AG versus AA). Figure H in S2 File: Forest plot of association between the rs1061581 polymorphism and NIHL (Recessive model: GG versus AG+AA). Figure I in S2 File: Forest plot of association between the rs2075800 polymorphism and NIHL (Heterozygote model: CT versus CC). Figure J in S2 File: Forest plot of association between the rs2075800 polymorphism and NIHL (Homozygote model: TT versus CC). Figure K in S2 File: Forest plot of association between the rs2075800 polymorphism and NIHL (Dominant model: TT+CT versus CC). Figure L in S2 File: Forest plot of association between the rs2075800 polymorphism and NIHL (Recessive model: TT versus CT+CC). Figure M in S2 File: Forest plot of association between the rs2227956 polymorphism and NIHL (Heterozygote model: TC versus TT). Figure N in S2 File: Forest plot of association between the rs2227956 polymorphism and NIHL (Homozygote model: CC versus TT). Figure O in S2 File: Forest plot of association between the rs2227956 polymorphism and NIHL (Dominant model: CC+TC versus TT). Figure P in S2 File: Forest plot of association betw [file pone.0188539.s002.zip › S2 File/Figure M.tif]

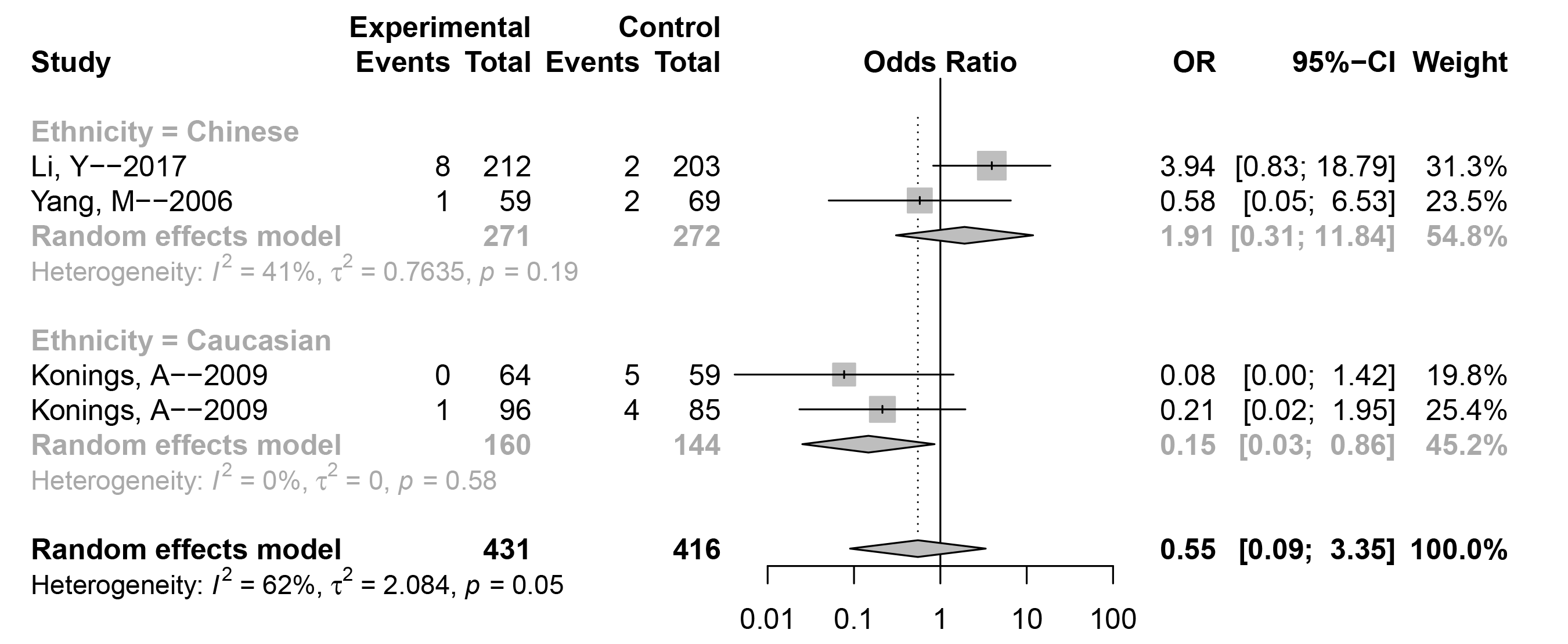

Supplement: S2 File — Forest plot (Figure A—Figure T). (ZIP). Figure A in S2 File: Forest plot of association between the rs1043618 polymorphism and NIHL (Heterozygote model: GC versus GG). Figure B in S2 File: Forest plot of association between the rs1043618 polymorphism and NIHL (Homozygote model: CC versus GG). Figure C in S2 File: Forest plot of association between the rs1043618 polymorphism and NIHL (Dominant model: CC+GC versus GG). Figure D in S2 File: Forest plot of association between the rs1043618 polymorphism and NIHL (Recessive model: CC versus CG+GG). Figure E in S2 File: Forest plot of association between the rs1061581 polymorphism and NIHL (Heterozygote model: AG versus AA). Figure F in S2 File: Forest plot of association between the rs1061581 polymorphism and NIHL (Homozygote model: GG versus AA). Figure G in S2 File: Forest plot of association between the rs1061581 polymorphism and NIHL (Dominant model: GG+AG versus AA). Figure H in S2 File: Forest plot of association between the rs1061581 polymorphism and NIHL (Recessive model: GG versus AG+AA). Figure I in S2 File: Forest plot of association between the rs2075800 polymorphism and NIHL (Heterozygote model: CT versus CC). Figure J in S2 File: Forest plot of association between the rs2075800 polymorphism and NIHL (Homozygote model: TT versus CC). Figure K in S2 File: Forest plot of association between the rs2075800 polymorphism and NIHL (Dominant model: TT+CT versus CC). Figure L in S2 File: Forest plot of association between the rs2075800 polymorphism and NIHL (Recessive model: TT versus CT+CC). Figure M in S2 File: Forest plot of association between the rs2227956 polymorphism and NIHL (Heterozygote model: TC versus TT). Figure N in S2 File: Forest plot of association between the rs2227956 polymorphism and NIHL (Homozygote model: CC versus TT). Figure O in S2 File: Forest plot of association between the rs2227956 polymorphism and NIHL (Dominant model: CC+TC versus TT). Figure P in S2 File: Forest plot of association betw [file pone.0188539.s002.zip › S2 File/Figure N.tif]

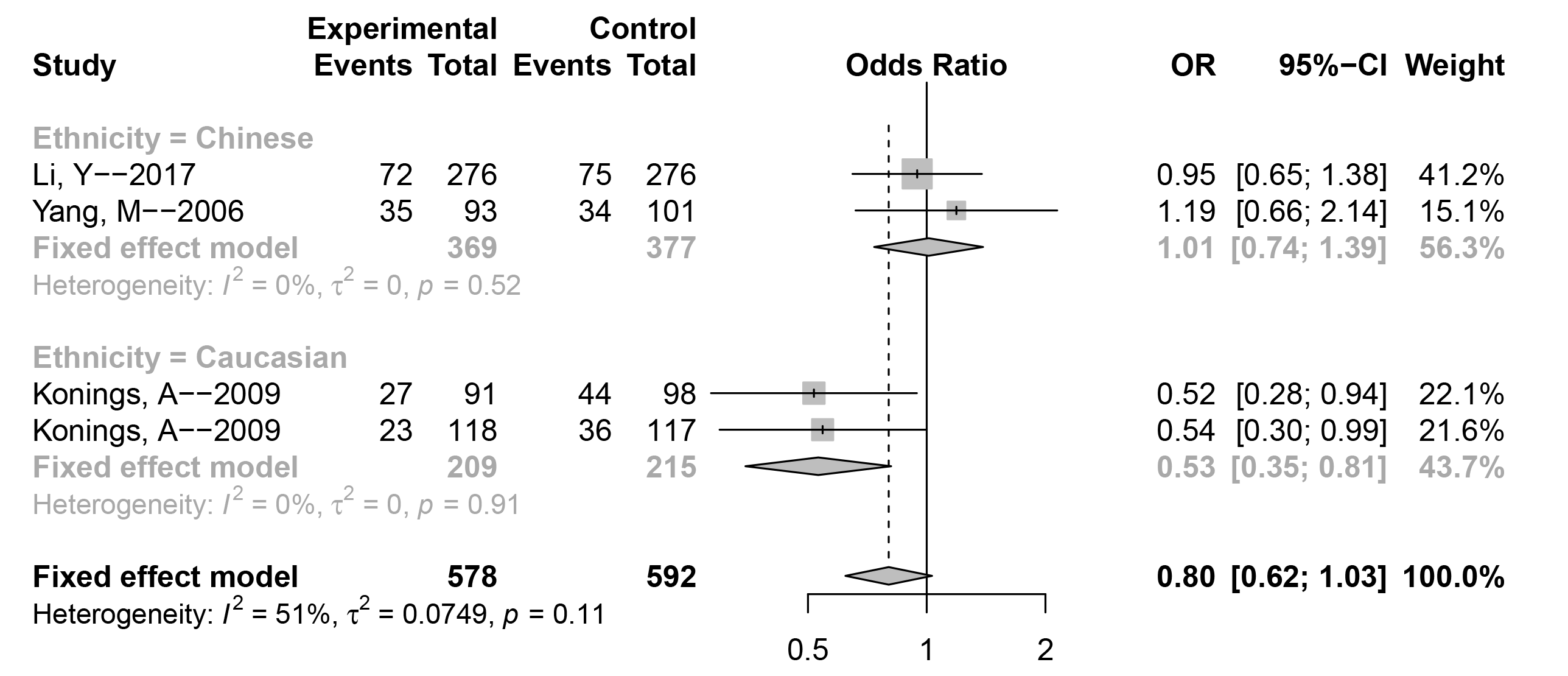

Supplement: S2 File — Forest plot (Figure A—Figure T). (ZIP). Figure A in S2 File: Forest plot of association between the rs1043618 polymorphism and NIHL (Heterozygote model: GC versus GG). Figure B in S2 File: Forest plot of association between the rs1043618 polymorphism and NIHL (Homozygote model: CC versus GG). Figure C in S2 File: Forest plot of association between the rs1043618 polymorphism and NIHL (Dominant model: CC+GC versus GG). Figure D in S2 File: Forest plot of association between the rs1043618 polymorphism and NIHL (Recessive model: CC versus CG+GG). Figure E in S2 File: Forest plot of association between the rs1061581 polymorphism and NIHL (Heterozygote model: AG versus AA). Figure F in S2 File: Forest plot of association between the rs1061581 polymorphism and NIHL (Homozygote model: GG versus AA). Figure G in S2 File: Forest plot of association between the rs1061581 polymorphism and NIHL (Dominant model: GG+AG versus AA). Figure H in S2 File: Forest plot of association between the rs1061581 polymorphism and NIHL (Recessive model: GG versus AG+AA). Figure I in S2 File: Forest plot of association between the rs2075800 polymorphism and NIHL (Heterozygote model: CT versus CC). Figure J in S2 File: Forest plot of association between the rs2075800 polymorphism and NIHL (Homozygote model: TT versus CC). Figure K in S2 File: Forest plot of association between the rs2075800 polymorphism and NIHL (Dominant model: TT+CT versus CC). Figure L in S2 File: Forest plot of association between the rs2075800 polymorphism and NIHL (Recessive model: TT versus CT+CC). Figure M in S2 File: Forest plot of association between the rs2227956 polymorphism and NIHL (Heterozygote model: TC versus TT). Figure N in S2 File: Forest plot of association between the rs2227956 polymorphism and NIHL (Homozygote model: CC versus TT). Figure O in S2 File: Forest plot of association between the rs2227956 polymorphism and NIHL (Dominant model: CC+TC versus TT). Figure P in S2 File: Forest plot of association betw [file pone.0188539.s002.zip › S2 File/Figure O.tif]

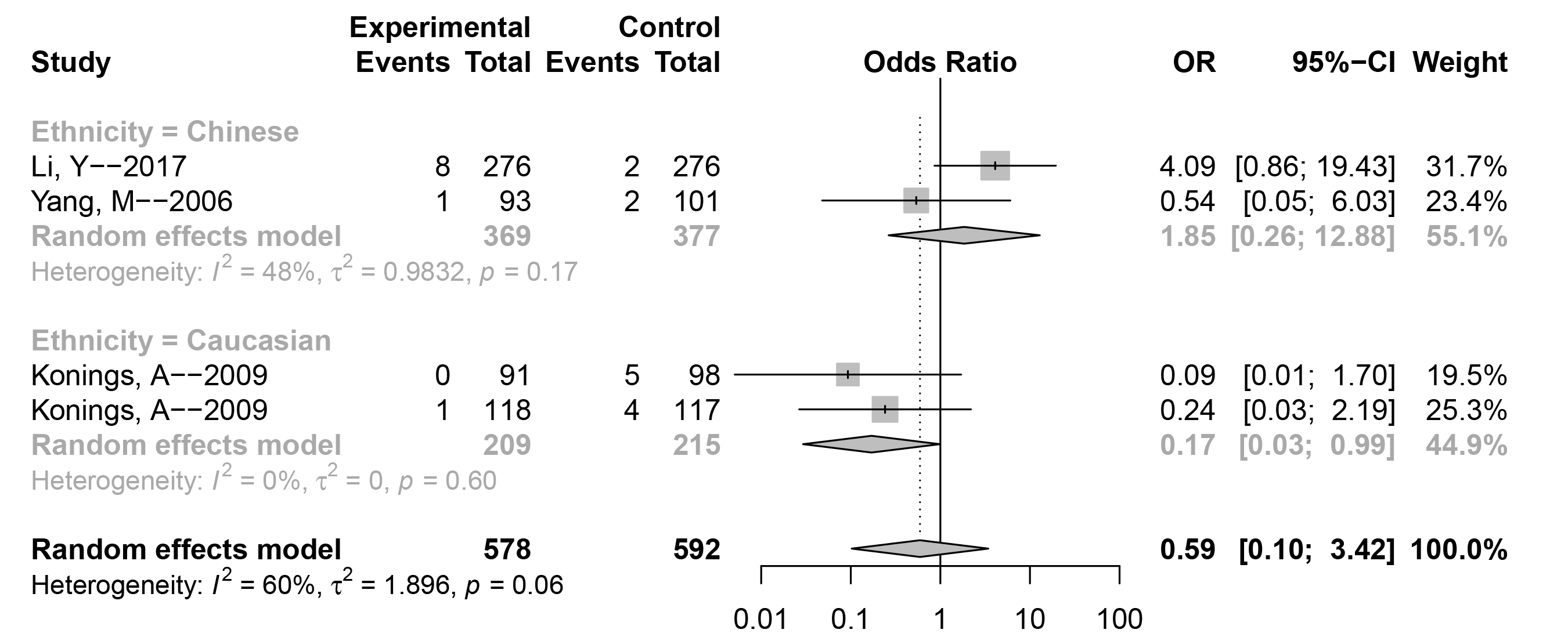

Supplement: S2 File — Forest plot (Figure A—Figure T). (ZIP). Figure A in S2 File: Forest plot of association between the rs1043618 polymorphism and NIHL (Heterozygote model: GC versus GG). Figure B in S2 File: Forest plot of association between the rs1043618 polymorphism and NIHL (Homozygote model: CC versus GG). Figure C in S2 File: Forest plot of association between the rs1043618 polymorphism and NIHL (Dominant model: CC+GC versus GG). Figure D in S2 File: Forest plot of association between the rs1043618 polymorphism and NIHL (Recessive model: CC versus CG+GG). Figure E in S2 File: Forest plot of association between the rs1061581 polymorphism and NIHL (Heterozygote model: AG versus AA). Figure F in S2 File: Forest plot of association between the rs1061581 polymorphism and NIHL (Homozygote model: GG versus AA). Figure G in S2 File: Forest plot of association between the rs1061581 polymorphism and NIHL (Dominant model: GG+AG versus AA). Figure H in S2 File: Forest plot of association between the rs1061581 polymorphism and NIHL (Recessive model: GG versus AG+AA). Figure I in S2 File: Forest plot of association between the rs2075800 polymorphism and NIHL (Heterozygote model: CT versus CC). Figure J in S2 File: Forest plot of association between the rs2075800 polymorphism and NIHL (Homozygote model: TT versus CC). Figure K in S2 File: Forest plot of association between the rs2075800 polymorphism and NIHL (Dominant model: TT+CT versus CC). Figure L in S2 File: Forest plot of association between the rs2075800 polymorphism and NIHL (Recessive model: TT versus CT+CC). Figure M in S2 File: Forest plot of association between the rs2227956 polymorphism and NIHL (Heterozygote model: TC versus TT). Figure N in S2 File: Forest plot of association between the rs2227956 polymorphism and NIHL (Homozygote model: CC versus TT). Figure O in S2 File: Forest plot of association between the rs2227956 polymorphism and NIHL (Dominant model: CC+TC versus TT). Figure P in S2 File: Forest plot of association betw [file pone.0188539.s002.zip › S2 File/Figure P.tif]

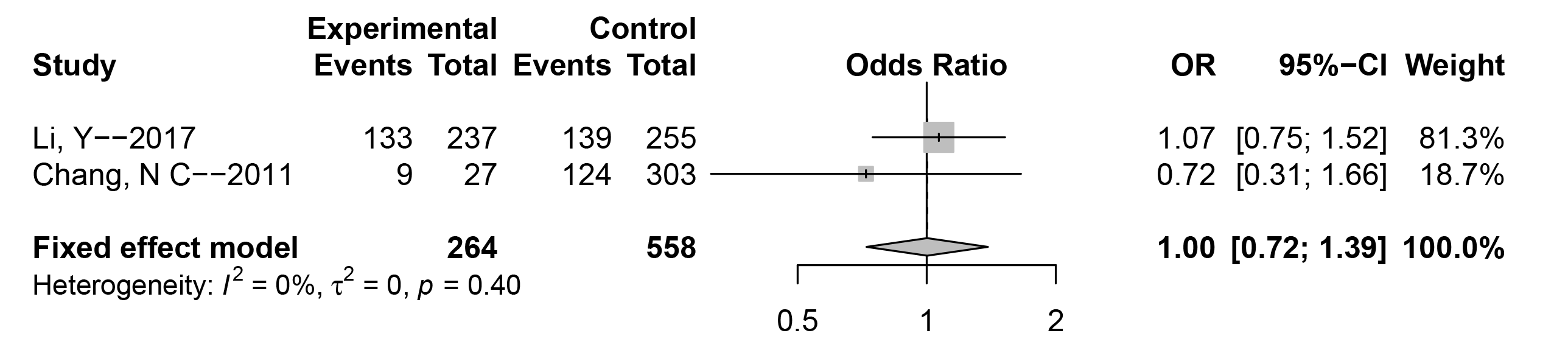

Supplement: S2 File — Forest plot (Figure A—Figure T). (ZIP). Figure A in S2 File: Forest plot of association between the rs1043618 polymorphism and NIHL (Heterozygote model: GC versus GG). Figure B in S2 File: Forest plot of association between the rs1043618 polymorphism and NIHL (Homozygote model: CC versus GG). Figure C in S2 File: Forest plot of association between the rs1043618 polymorphism and NIHL (Dominant model: CC+GC versus GG). Figure D in S2 File: Forest plot of association between the rs1043618 polymorphism and NIHL (Recessive model: CC versus CG+GG). Figure E in S2 File: Forest plot of association between the rs1061581 polymorphism and NIHL (Heterozygote model: AG versus AA). Figure F in S2 File: Forest plot of association between the rs1061581 polymorphism and NIHL (Homozygote model: GG versus AA). Figure G in S2 File: Forest plot of association between the rs1061581 polymorphism and NIHL (Dominant model: GG+AG versus AA). Figure H in S2 File: Forest plot of association between the rs1061581 polymorphism and NIHL (Recessive model: GG versus AG+AA). Figure I in S2 File: Forest plot of association between the rs2075800 polymorphism and NIHL (Heterozygote model: CT versus CC). Figure J in S2 File: Forest plot of association between the rs2075800 polymorphism and NIHL (Homozygote model: TT versus CC). Figure K in S2 File: Forest plot of association between the rs2075800 polymorphism and NIHL (Dominant model: TT+CT versus CC). Figure L in S2 File: Forest plot of association between the rs2075800 polymorphism and NIHL (Recessive model: TT versus CT+CC). Figure M in S2 File: Forest plot of association between the rs2227956 polymorphism and NIHL (Heterozygote model: TC versus TT). Figure N in S2 File: Forest plot of association between the rs2227956 polymorphism and NIHL (Homozygote model: CC versus TT). Figure O in S2 File: Forest plot of association between the rs2227956 polymorphism and NIHL (Dominant model: CC+TC versus TT). Figure P in S2 File: Forest plot of association betw [file pone.0188539.s002.zip › S2 File/Figure Q.tif]

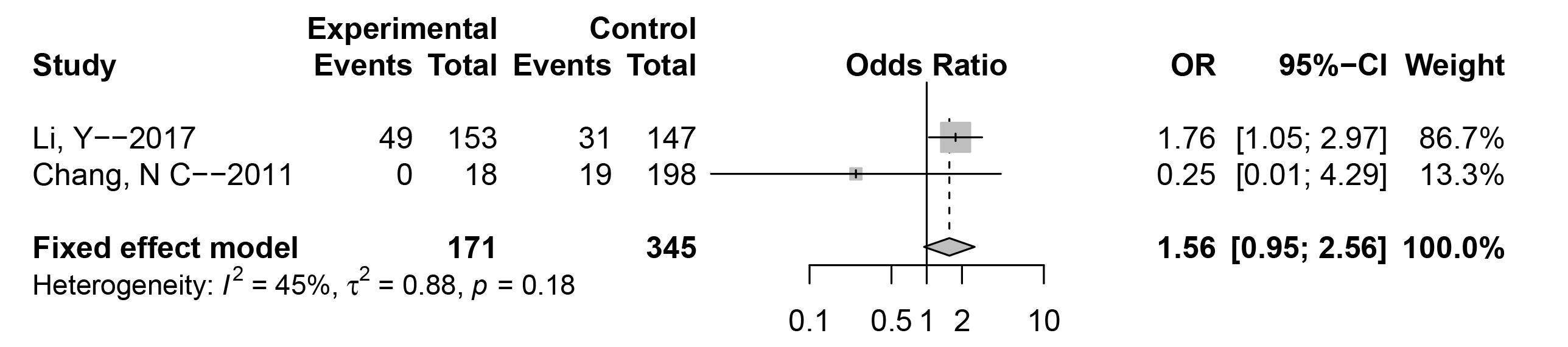

Supplement: S2 File — Forest plot (Figure A—Figure T). (ZIP). Figure A in S2 File: Forest plot of association between the rs1043618 polymorphism and NIHL (Heterozygote model: GC versus GG). Figure B in S2 File: Forest plot of association between the rs1043618 polymorphism and NIHL (Homozygote model: CC versus GG). Figure C in S2 File: Forest plot of association between the rs1043618 polymorphism and NIHL (Dominant model: CC+GC versus GG). Figure D in S2 File: Forest plot of association between the rs1043618 polymorphism and NIHL (Recessive model: CC versus CG+GG). Figure E in S2 File: Forest plot of association between the rs1061581 polymorphism and NIHL (Heterozygote model: AG versus AA). Figure F in S2 File: Forest plot of association between the rs1061581 polymorphism and NIHL (Homozygote model: GG versus AA). Figure G in S2 File: Forest plot of association between the rs1061581 polymorphism and NIHL (Dominant model: GG+AG versus AA). Figure H in S2 File: Forest plot of association between the rs1061581 polymorphism and NIHL (Recessive model: GG versus AG+AA). Figure I in S2 File: Forest plot of association between the rs2075800 polymorphism and NIHL (Heterozygote model: CT versus CC). Figure J in S2 File: Forest plot of association between the rs2075800 polymorphism and NIHL (Homozygote model: TT versus CC). Figure K in S2 File: Forest plot of association between the rs2075800 polymorphism and NIHL (Dominant model: TT+CT versus CC). Figure L in S2 File: Forest plot of association between the rs2075800 polymorphism and NIHL (Recessive model: TT versus CT+CC). Figure M in S2 File: Forest plot of association between the rs2227956 polymorphism and NIHL (Heterozygote model: TC versus TT). Figure N in S2 File: Forest plot of association between the rs2227956 polymorphism and NIHL (Homozygote model: CC versus TT). Figure O in S2 File: Forest plot of association between the rs2227956 polymorphism and NIHL (Dominant model: CC+TC versus TT). Figure P in S2 File: Forest plot of association betw [file pone.0188539.s002.zip › S2 File/Figure R.tif]

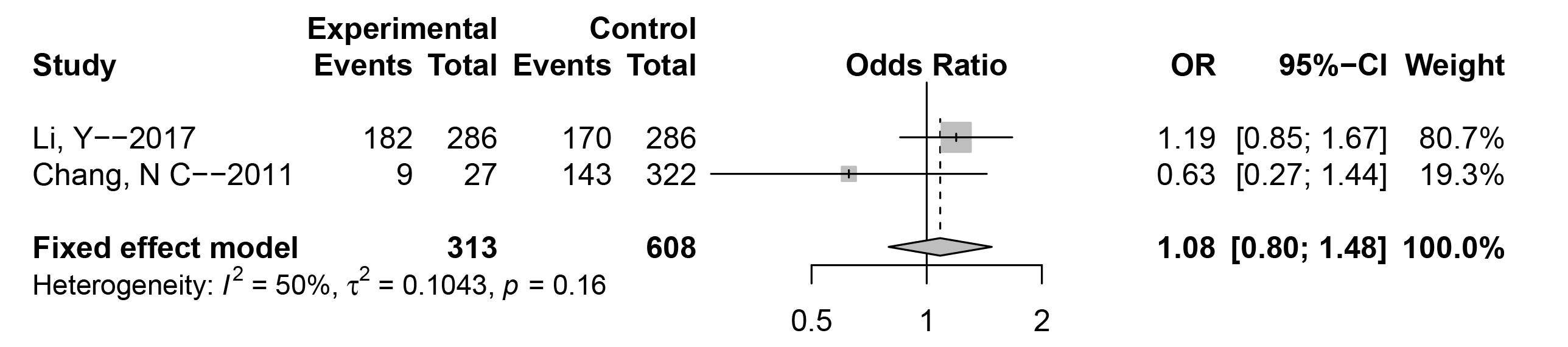

Supplement: S2 File — Forest plot (Figure A—Figure T). (ZIP). Figure A in S2 File: Forest plot of association between the rs1043618 polymorphism and NIHL (Heterozygote model: GC versus GG). Figure B in S2 File: Forest plot of association between the rs1043618 polymorphism and NIHL (Homozygote model: CC versus GG). Figure C in S2 File: Forest plot of association between the rs1043618 polymorphism and NIHL (Dominant model: CC+GC versus GG). Figure D in S2 File: Forest plot of association between the rs1043618 polymorphism and NIHL (Recessive model: CC versus CG+GG). Figure E in S2 File: Forest plot of association between the rs1061581 polymorphism and NIHL (Heterozygote model: AG versus AA). Figure F in S2 File: Forest plot of association between the rs1061581 polymorphism and NIHL (Homozygote model: GG versus AA). Figure G in S2 File: Forest plot of association between the rs1061581 polymorphism and NIHL (Dominant model: GG+AG versus AA). Figure H in S2 File: Forest plot of association between the rs1061581 polymorphism and NIHL (Recessive model: GG versus AG+AA). Figure I in S2 File: Forest plot of association between the rs2075800 polymorphism and NIHL (Heterozygote model: CT versus CC). Figure J in S2 File: Forest plot of association between the rs2075800 polymorphism and NIHL (Homozygote model: TT versus CC). Figure K in S2 File: Forest plot of association between the rs2075800 polymorphism and NIHL (Dominant model: TT+CT versus CC). Figure L in S2 File: Forest plot of association between the rs2075800 polymorphism and NIHL (Recessive model: TT versus CT+CC). Figure M in S2 File: Forest plot of association between the rs2227956 polymorphism and NIHL (Heterozygote model: TC versus TT). Figure N in S2 File: Forest plot of association between the rs2227956 polymorphism and NIHL (Homozygote model: CC versus TT). Figure O in S2 File: Forest plot of association between the rs2227956 polymorphism and NIHL (Dominant model: CC+TC versus TT). Figure P in S2 File: Forest plot of association betw [file pone.0188539.s002.zip › S2 File/Figure S.tif]

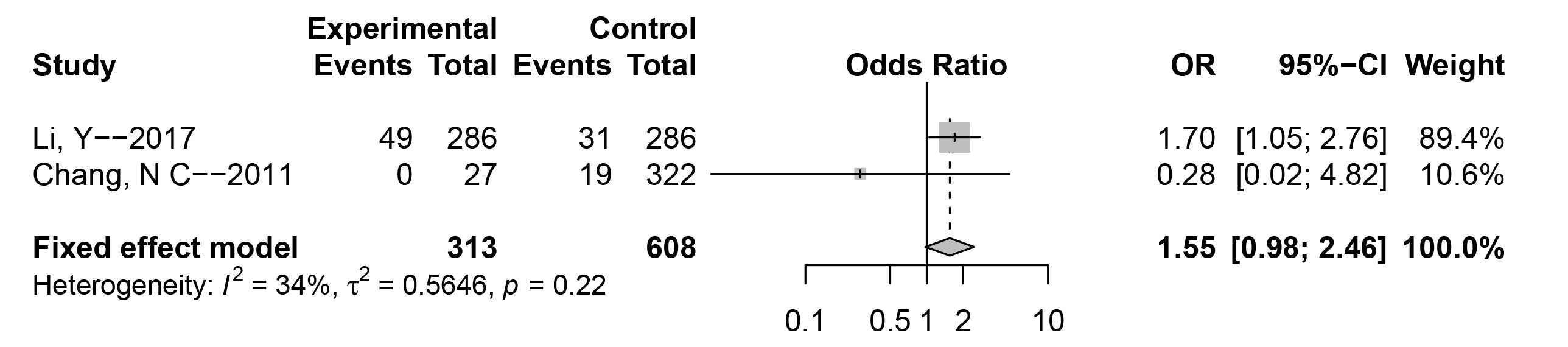

Supplement: S2 File — Forest plot (Figure A—Figure T). (ZIP). Figure A in S2 File: Forest plot of association between the rs1043618 polymorphism and NIHL (Heterozygote model: GC versus GG). Figure B in S2 File: Forest plot of association between the rs1043618 polymorphism and NIHL (Homozygote model: CC versus GG). Figure C in S2 File: Forest plot of association between the rs1043618 polymorphism and NIHL (Dominant model: CC+GC versus GG). Figure D in S2 File: Forest plot of association between the rs1043618 polymorphism and NIHL (Recessive model: CC versus CG+GG). Figure E in S2 File: Forest plot of association between the rs1061581 polymorphism and NIHL (Heterozygote model: AG versus AA). Figure F in S2 File: Forest plot of association between the rs1061581 polymorphism and NIHL (Homozygote model: GG versus AA). Figure G in S2 File: Forest plot of association between the rs1061581 polymorphism and NIHL (Dominant model: GG+AG versus AA). Figure H in S2 File: Forest plot of association between the rs1061581 polymorphism and NIHL (Recessive model: GG versus AG+AA). Figure I in S2 File: Forest plot of association between the rs2075800 polymorphism and NIHL (Heterozygote model: CT versus CC). Figure J in S2 File: Forest plot of association between the rs2075800 polymorphism and NIHL (Homozygote model: TT versus CC). Figure K in S2 File: Forest plot of association between the rs2075800 polymorphism and NIHL (Dominant model: TT+CT versus CC). Figure L in S2 File: Forest plot of association between the rs2075800 polymorphism and NIHL (Recessive model: TT versus CT+CC). Figure M in S2 File: Forest plot of association between the rs2227956 polymorphism and NIHL (Heterozygote model: TC versus TT). Figure N in S2 File: Forest plot of association between the rs2227956 polymorphism and NIHL (Homozygote model: CC versus TT). Figure O in S2 File: Forest plot of association between the rs2227956 polymorphism and NIHL (Dominant model: CC+TC versus TT). Figure P in S2 File: Forest plot of association betw [file pone.0188539.s002.zip › S2 File/Figure T.tif]

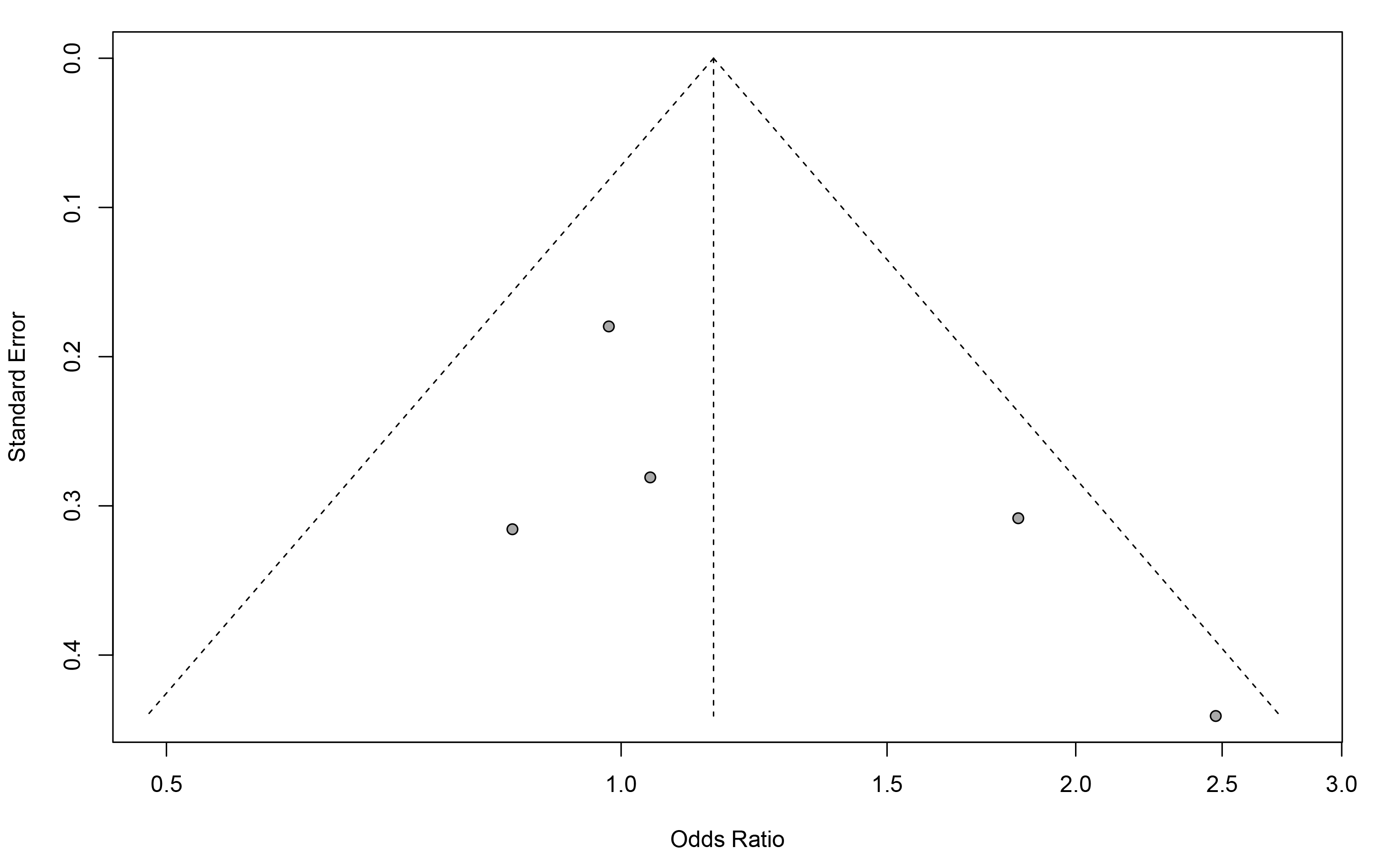

Supplement: S3 File — Begg’s funnel plot (Figure A—Figure T). (ZIP). Figure A in S3 File: Begg’s funnel plot of association between the rs1043618 polymorphism and NIHL (Heterozygote model: GC versus GG). Figure B in S3 File: Begg’s funnel plot of association between the rs1043618 polymorphism and NIHL (Homozygote model: CC versus GG). Figure C in S3 File: Begg’s funnel plot of association between the rs1043618 polymorphism and NIHL (Dominant model: CC+GC versus GG). Figure D in S3 File: Begg’s funnel plot of association between the rs1043618 polymorphism and NIHL (Recessive model: CC versus CG+GG). Figure E in S3 File: Begg’s funnel plot of association between the rs1061581 polymorphism and NIHL (Heterozygote model: AG versus AA). Figure F in S3 File: Begg’s funnel plot of association between the rs1061581 polymorphism and NIHL (Homozygote model: GG versus AA). Figure G in S3 File: Begg’s funnel plot of association between the rs1061581 polymorphism and NIHL (Dominant model: GG+AG versus AA). Figure H in S3 File: Begg’s funnel plot of association between the rs1061581 polymorphism and NIHL (Recessive model: GG versus AG+AA). Figure I in S3 File: Begg’s funnel plot of association between the rs2075800 polymorphism and NIHL (Heterozygote model: CT versus CC). Figure J in S3 File: Begg’s funnel plot of association between the rs2075800 polymorphism and NIHL (Homozygote model: TT versus CC). Figure K in S3 File: Begg’s funnel plot of association between the rs2075800 polymorphism and NIHL (Dominant model: TT+CT versus CC). Figure L in S3 File: Begg’s funnel plot of association between the rs2075800 polymorphism and NIHL (Recessive model: TT versus CT+CC). Figure M in S3 File: Begg’s funnel plot of association between the rs2227956 polymorphism and NIHL (Heterozygote model: TC versus TT). Figure N in S3 File: Begg’s funnel plot of association between the rs2227956 polymorphism and NIHL (Homozygote model: CC versus TT). Figure O in S3 File: Begg’s funnel plot of association between the rs22279 [file pone.0188539.s003.zip › S3 File/Figure A.tif]

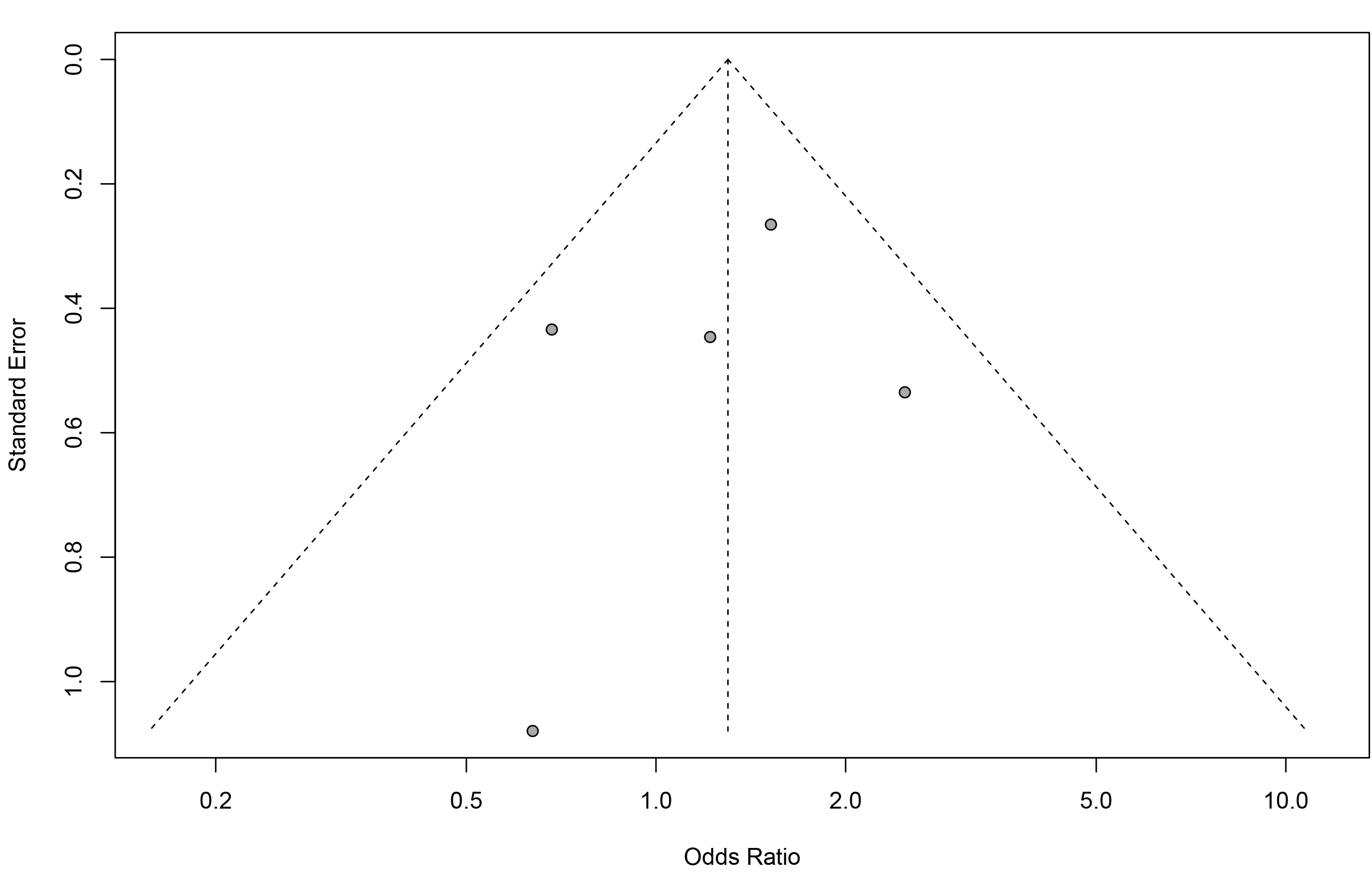

Supplement: S3 File — Begg’s funnel plot (Figure A—Figure T). (ZIP). Figure A in S3 File: Begg’s funnel plot of association between the rs1043618 polymorphism and NIHL (Heterozygote model: GC versus GG). Figure B in S3 File: Begg’s funnel plot of association between the rs1043618 polymorphism and NIHL (Homozygote model: CC versus GG). Figure C in S3 File: Begg’s funnel plot of association between the rs1043618 polymorphism and NIHL (Dominant model: CC+GC versus GG). Figure D in S3 File: Begg’s funnel plot of association between the rs1043618 polymorphism and NIHL (Recessive model: CC versus CG+GG). Figure E in S3 File: Begg’s funnel plot of association between the rs1061581 polymorphism and NIHL (Heterozygote model: AG versus AA). Figure F in S3 File: Begg’s funnel plot of association between the rs1061581 polymorphism and NIHL (Homozygote model: GG versus AA). Figure G in S3 File: Begg’s funnel plot of association between the rs1061581 polymorphism and NIHL (Dominant model: GG+AG versus AA). Figure H in S3 File: Begg’s funnel plot of association between the rs1061581 polymorphism and NIHL (Recessive model: GG versus AG+AA). Figure I in S3 File: Begg’s funnel plot of association between the rs2075800 polymorphism and NIHL (Heterozygote model: CT versus CC). Figure J in S3 File: Begg’s funnel plot of association between the rs2075800 polymorphism and NIHL (Homozygote model: TT versus CC). Figure K in S3 File: Begg’s funnel plot of association between the rs2075800 polymorphism and NIHL (Dominant model: TT+CT versus CC). Figure L in S3 File: Begg’s funnel plot of association between the rs2075800 polymorphism and NIHL (Recessive model: TT versus CT+CC). Figure M in S3 File: Begg’s funnel plot of association between the rs2227956 polymorphism and NIHL (Heterozygote model: TC versus TT). Figure N in S3 File: Begg’s funnel plot of association between the rs2227956 polymorphism and NIHL (Homozygote model: CC versus TT). Figure O in S3 File: Begg’s funnel plot of association between the rs22279 [file pone.0188539.s003.zip › S3 File/Figure B.tif]

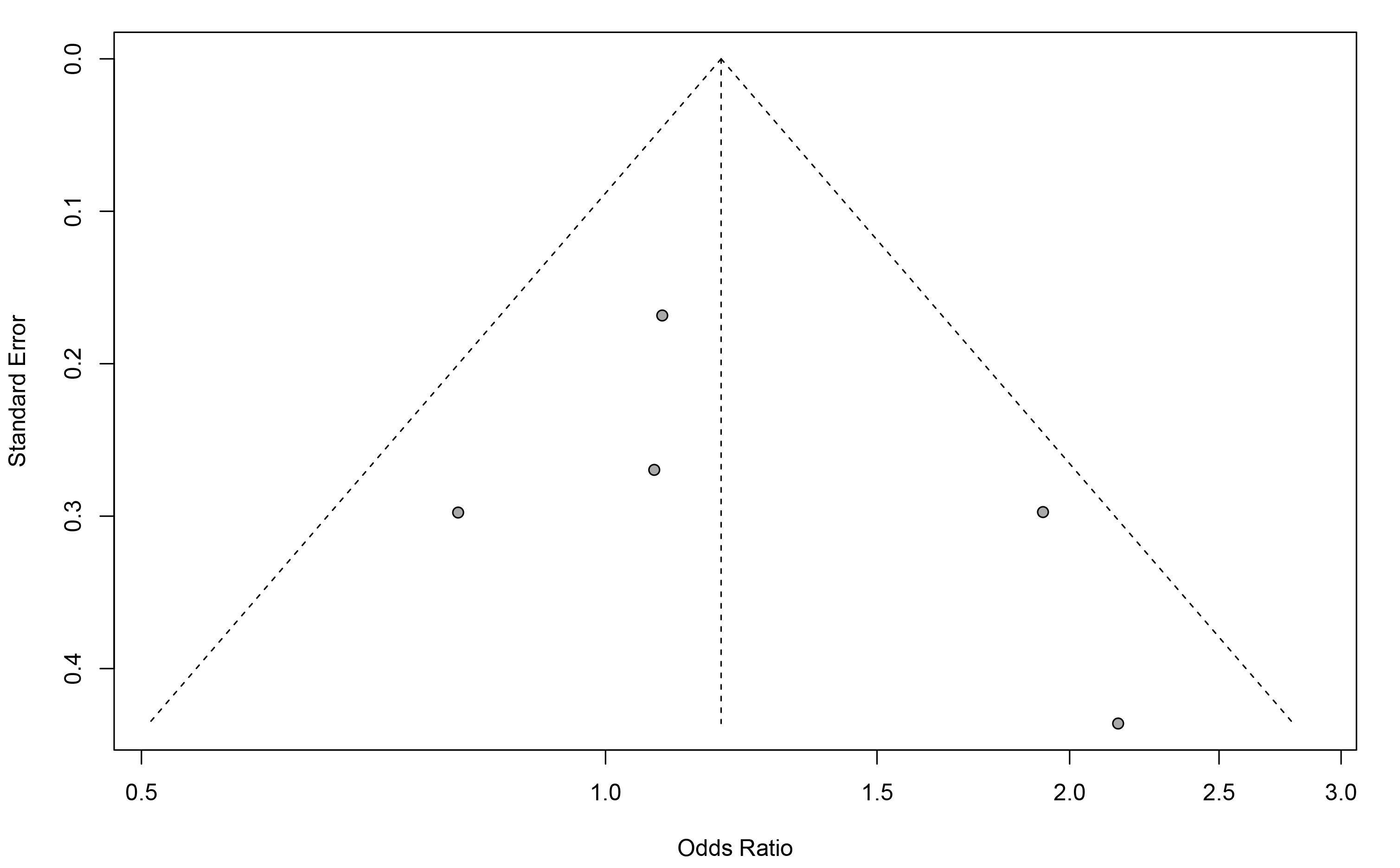

Supplement: S3 File — Begg’s funnel plot (Figure A—Figure T). (ZIP). Figure A in S3 File: Begg’s funnel plot of association between the rs1043618 polymorphism and NIHL (Heterozygote model: GC versus GG). Figure B in S3 File: Begg’s funnel plot of association between the rs1043618 polymorphism and NIHL (Homozygote model: CC versus GG). Figure C in S3 File: Begg’s funnel plot of association between the rs1043618 polymorphism and NIHL (Dominant model: CC+GC versus GG). Figure D in S3 File: Begg’s funnel plot of association between the rs1043618 polymorphism and NIHL (Recessive model: CC versus CG+GG). Figure E in S3 File: Begg’s funnel plot of association between the rs1061581 polymorphism and NIHL (Heterozygote model: AG versus AA). Figure F in S3 File: Begg’s funnel plot of association between the rs1061581 polymorphism and NIHL (Homozygote model: GG versus AA). Figure G in S3 File: Begg’s funnel plot of association between the rs1061581 polymorphism and NIHL (Dominant model: GG+AG versus AA). Figure H in S3 File: Begg’s funnel plot of association between the rs1061581 polymorphism and NIHL (Recessive model: GG versus AG+AA). Figure I in S3 File: Begg’s funnel plot of association between the rs2075800 polymorphism and NIHL (Heterozygote model: CT versus CC). Figure J in S3 File: Begg’s funnel plot of association between the rs2075800 polymorphism and NIHL (Homozygote model: TT versus CC). Figure K in S3 File: Begg’s funnel plot of association between the rs2075800 polymorphism and NIHL (Dominant model: TT+CT versus CC). Figure L in S3 File: Begg’s funnel plot of association between the rs2075800 polymorphism and NIHL (Recessive model: TT versus CT+CC). Figure M in S3 File: Begg’s funnel plot of association between the rs2227956 polymorphism and NIHL (Heterozygote model: TC versus TT). Figure N in S3 File: Begg’s funnel plot of association between the rs2227956 polymorphism and NIHL (Homozygote model: CC versus TT). Figure O in S3 File: Begg’s funnel plot of association between the rs22279 [file pone.0188539.s003.zip › S3 File/Figure C.tif]

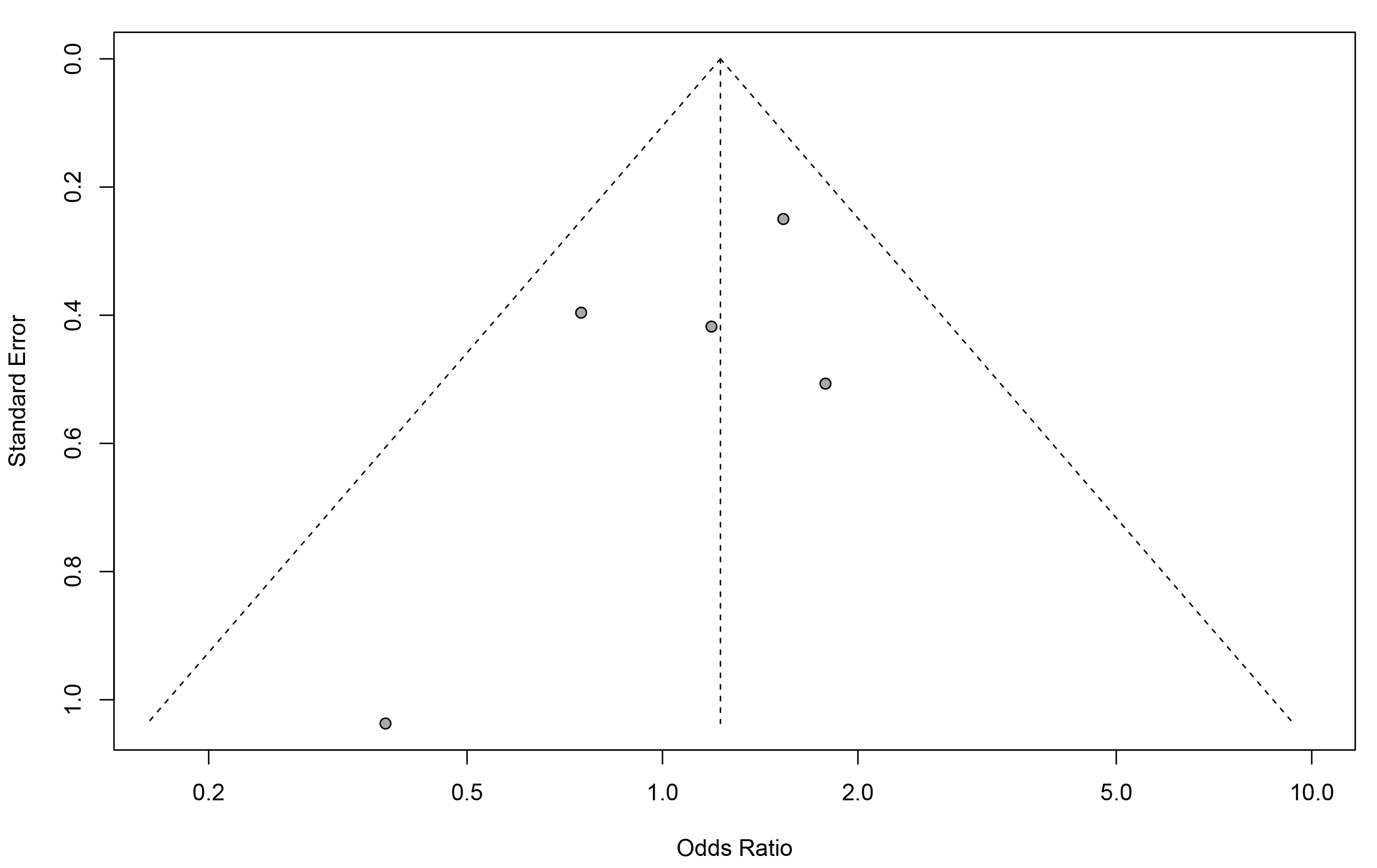

Supplement: S3 File — Begg’s funnel plot (Figure A—Figure T). (ZIP). Figure A in S3 File: Begg’s funnel plot of association between the rs1043618 polymorphism and NIHL (Heterozygote model: GC versus GG). Figure B in S3 File: Begg’s funnel plot of association between the rs1043618 polymorphism and NIHL (Homozygote model: CC versus GG). Figure C in S3 File: Begg’s funnel plot of association between the rs1043618 polymorphism and NIHL (Dominant model: CC+GC versus GG). Figure D in S3 File: Begg’s funnel plot of association between the rs1043618 polymorphism and NIHL (Recessive model: CC versus CG+GG). Figure E in S3 File: Begg’s funnel plot of association between the rs1061581 polymorphism and NIHL (Heterozygote model: AG versus AA). Figure F in S3 File: Begg’s funnel plot of association between the rs1061581 polymorphism and NIHL (Homozygote model: GG versus AA). Figure G in S3 File: Begg’s funnel plot of association between the rs1061581 polymorphism and NIHL (Dominant model: GG+AG versus AA). Figure H in S3 File: Begg’s funnel plot of association between the rs1061581 polymorphism and NIHL (Recessive model: GG versus AG+AA). Figure I in S3 File: Begg’s funnel plot of association between the rs2075800 polymorphism and NIHL (Heterozygote model: CT versus CC). Figure J in S3 File: Begg’s funnel plot of association between the rs2075800 polymorphism and NIHL (Homozygote model: TT versus CC). Figure K in S3 File: Begg’s funnel plot of association between the rs2075800 polymorphism and NIHL (Dominant model: TT+CT versus CC). Figure L in S3 File: Begg’s funnel plot of association between the rs2075800 polymorphism and NIHL (Recessive model: TT versus CT+CC). Figure M in S3 File: Begg’s funnel plot of association between the rs2227956 polymorphism and NIHL (Heterozygote model: TC versus TT). Figure N in S3 File: Begg’s funnel plot of association between the rs2227956 polymorphism and NIHL (Homozygote model: CC versus TT). Figure O in S3 File: Begg’s funnel plot of association between the rs22279 [file pone.0188539.s003.zip › S3 File/Figure D.tif]

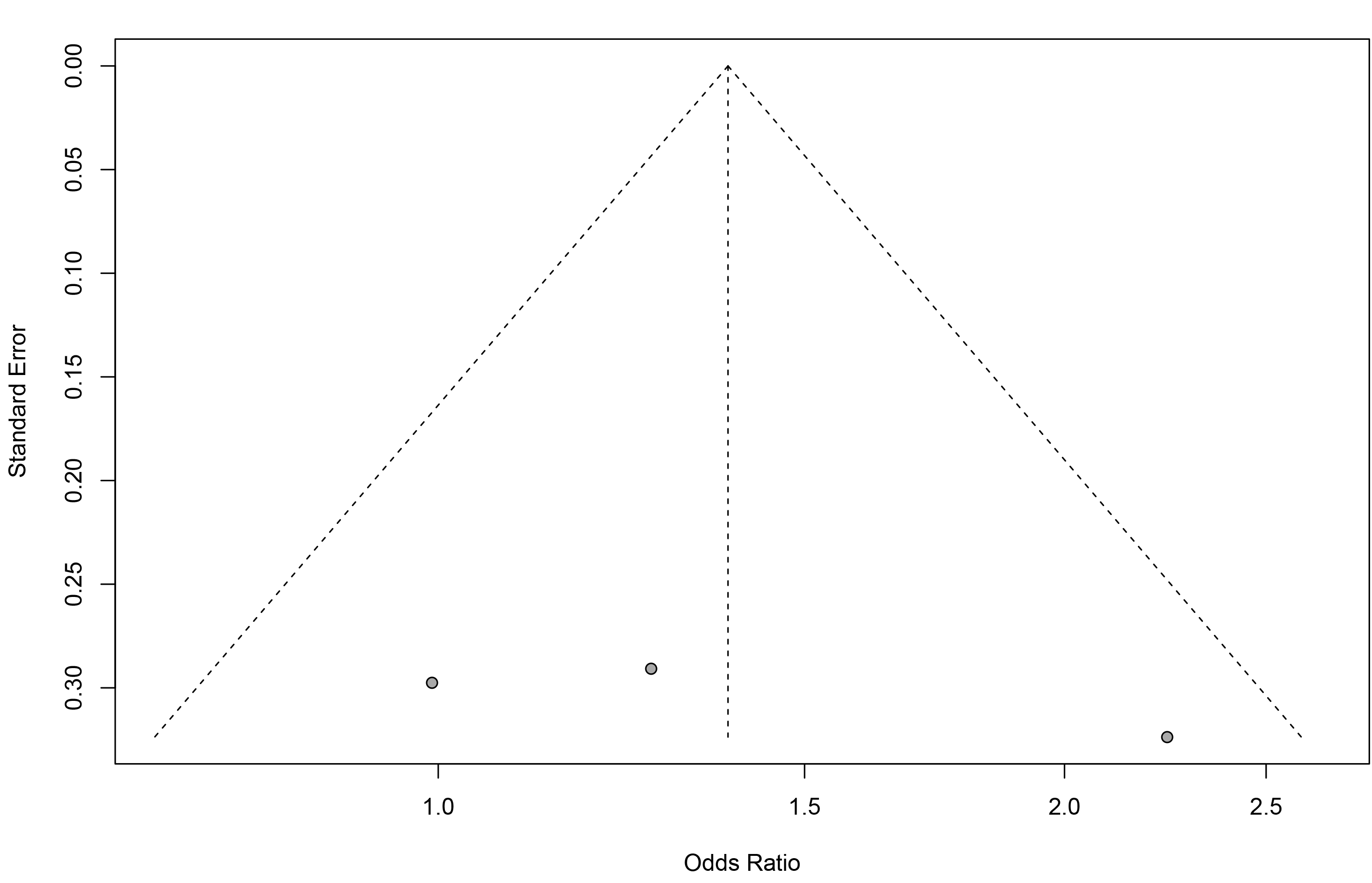

Supplement: S3 File — Begg’s funnel plot (Figure A—Figure T). (ZIP). Figure A in S3 File: Begg’s funnel plot of association between the rs1043618 polymorphism and NIHL (Heterozygote model: GC versus GG). Figure B in S3 File: Begg’s funnel plot of association between the rs1043618 polymorphism and NIHL (Homozygote model: CC versus GG). Figure C in S3 File: Begg’s funnel plot of association between the rs1043618 polymorphism and NIHL (Dominant model: CC+GC versus GG). Figure D in S3 File: Begg’s funnel plot of association between the rs1043618 polymorphism and NIHL (Recessive model: CC versus CG+GG). Figure E in S3 File: Begg’s funnel plot of association between the rs1061581 polymorphism and NIHL (Heterozygote model: AG versus AA). Figure F in S3 File: Begg’s funnel plot of association between the rs1061581 polymorphism and NIHL (Homozygote model: GG versus AA). Figure G in S3 File: Begg’s funnel plot of association between the rs1061581 polymorphism and NIHL (Dominant model: GG+AG versus AA). Figure H in S3 File: Begg’s funnel plot of association between the rs1061581 polymorphism and NIHL (Recessive model: GG versus AG+AA). Figure I in S3 File: Begg’s funnel plot of association between the rs2075800 polymorphism and NIHL (Heterozygote model: CT versus CC). Figure J in S3 File: Begg’s funnel plot of association between the rs2075800 polymorphism and NIHL (Homozygote model: TT versus CC). Figure K in S3 File: Begg’s funnel plot of association between the rs2075800 polymorphism and NIHL (Dominant model: TT+CT versus CC). Figure L in S3 File: Begg’s funnel plot of association between the rs2075800 polymorphism and NIHL (Recessive model: TT versus CT+CC). Figure M in S3 File: Begg’s funnel plot of association between the rs2227956 polymorphism and NIHL (Heterozygote model: TC versus TT). Figure N in S3 File: Begg’s funnel plot of association between the rs2227956 polymorphism and NIHL (Homozygote model: CC versus TT). Figure O in S3 File: Begg’s funnel plot of association between the rs22279 [file pone.0188539.s003.zip › S3 File/Figure E.tif]

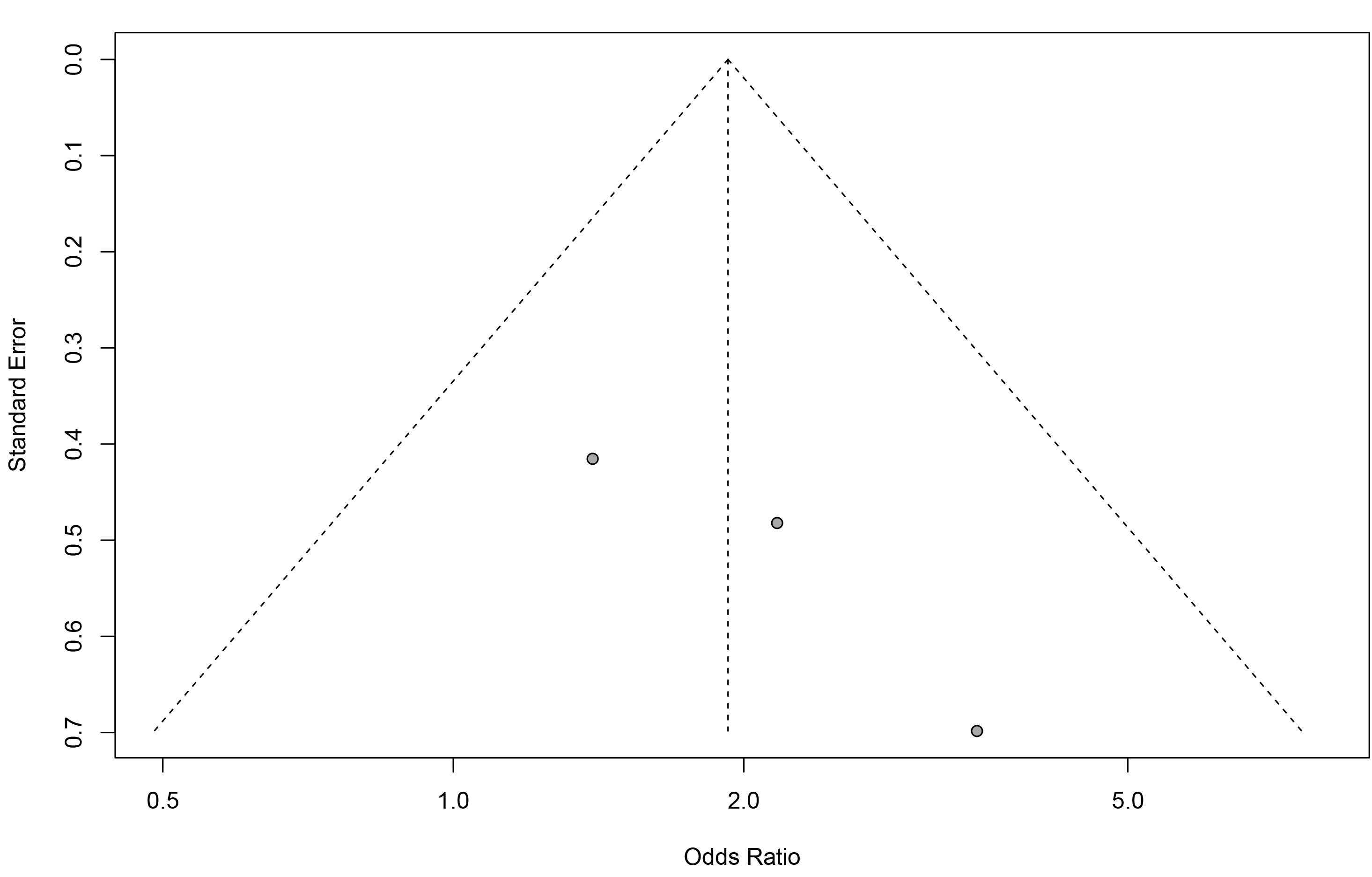

Supplement: S3 File — Begg’s funnel plot (Figure A—Figure T). (ZIP). Figure A in S3 File: Begg’s funnel plot of association between the rs1043618 polymorphism and NIHL (Heterozygote model: GC versus GG). Figure B in S3 File: Begg’s funnel plot of association between the rs1043618 polymorphism and NIHL (Homozygote model: CC versus GG). Figure C in S3 File: Begg’s funnel plot of association between the rs1043618 polymorphism and NIHL (Dominant model: CC+GC versus GG). Figure D in S3 File: Begg’s funnel plot of association between the rs1043618 polymorphism and NIHL (Recessive model: CC versus CG+GG). Figure E in S3 File: Begg’s funnel plot of association between the rs1061581 polymorphism and NIHL (Heterozygote model: AG versus AA). Figure F in S3 File: Begg’s funnel plot of association between the rs1061581 polymorphism and NIHL (Homozygote model: GG versus AA). Figure G in S3 File: Begg’s funnel plot of association between the rs1061581 polymorphism and NIHL (Dominant model: GG+AG versus AA). Figure H in S3 File: Begg’s funnel plot of association between the rs1061581 polymorphism and NIHL (Recessive model: GG versus AG+AA). Figure I in S3 File: Begg’s funnel plot of association between the rs2075800 polymorphism and NIHL (Heterozygote model: CT versus CC). Figure J in S3 File: Begg’s funnel plot of association between the rs2075800 polymorphism and NIHL (Homozygote model: TT versus CC). Figure K in S3 File: Begg’s funnel plot of association between the rs2075800 polymorphism and NIHL (Dominant model: TT+CT versus CC). Figure L in S3 File: Begg’s funnel plot of association between the rs2075800 polymorphism and NIHL (Recessive model: TT versus CT+CC). Figure M in S3 File: Begg’s funnel plot of association between the rs2227956 polymorphism and NIHL (Heterozygote model: TC versus TT). Figure N in S3 File: Begg’s funnel plot of association between the rs2227956 polymorphism and NIHL (Homozygote model: CC versus TT). Figure O in S3 File: Begg’s funnel plot of association between the rs22279 [file pone.0188539.s003.zip › S3 File/Figure F.tif]

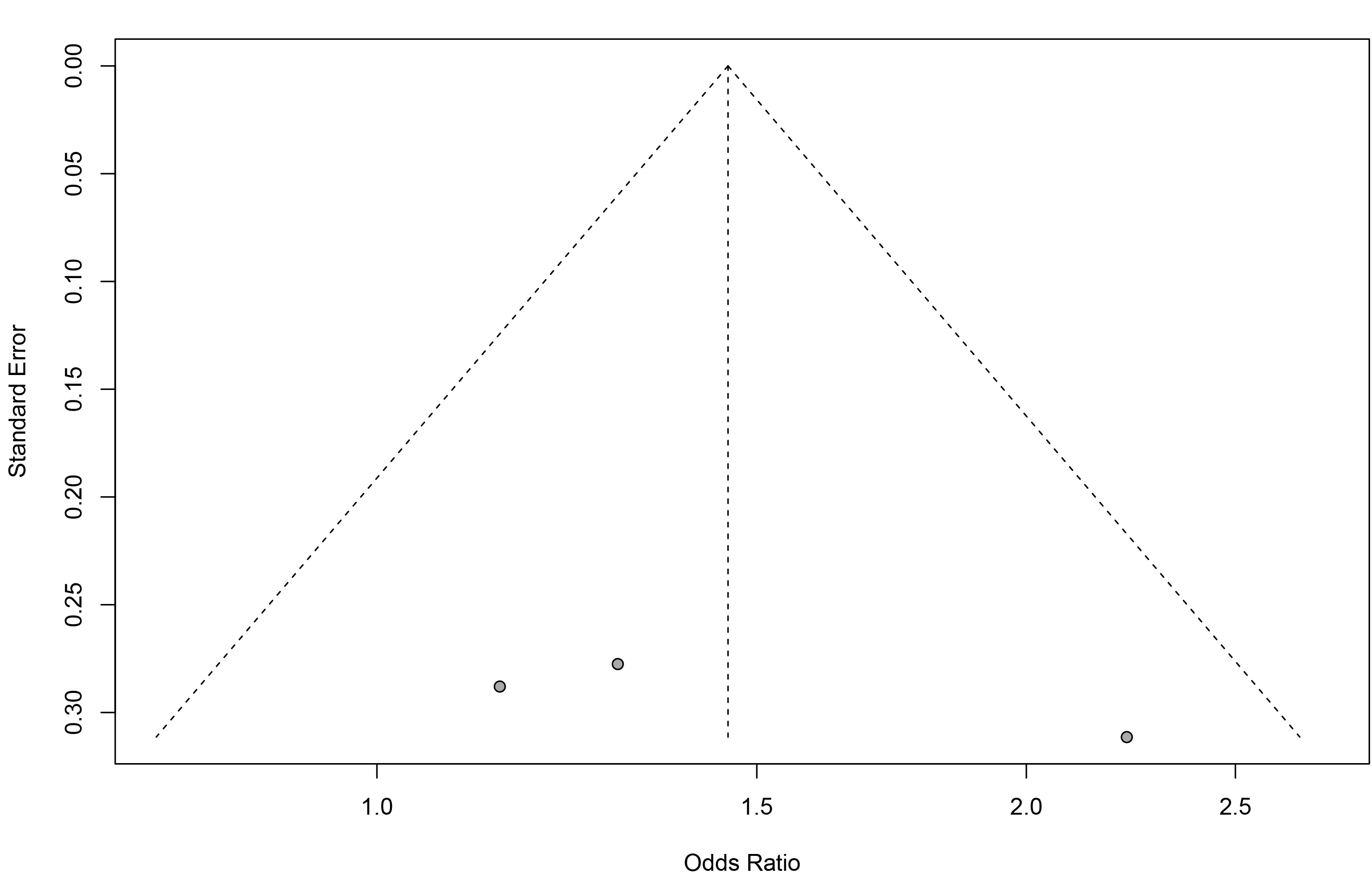

Supplement: S3 File — Begg’s funnel plot (Figure A—Figure T). (ZIP). Figure A in S3 File: Begg’s funnel plot of association between the rs1043618 polymorphism and NIHL (Heterozygote model: GC versus GG). Figure B in S3 File: Begg’s funnel plot of association between the rs1043618 polymorphism and NIHL (Homozygote model: CC versus GG). Figure C in S3 File: Begg’s funnel plot of association between the rs1043618 polymorphism and NIHL (Dominant model: CC+GC versus GG). Figure D in S3 File: Begg’s funnel plot of association between the rs1043618 polymorphism and NIHL (Recessive model: CC versus CG+GG). Figure E in S3 File: Begg’s funnel plot of association between the rs1061581 polymorphism and NIHL (Heterozygote model: AG versus AA). Figure F in S3 File: Begg’s funnel plot of association between the rs1061581 polymorphism and NIHL (Homozygote model: GG versus AA). Figure G in S3 File: Begg’s funnel plot of association between the rs1061581 polymorphism and NIHL (Dominant model: GG+AG versus AA). Figure H in S3 File: Begg’s funnel plot of association between the rs1061581 polymorphism and NIHL (Recessive model: GG versus AG+AA). Figure I in S3 File: Begg’s funnel plot of association between the rs2075800 polymorphism and NIHL (Heterozygote model: CT versus CC). Figure J in S3 File: Begg’s funnel plot of association between the rs2075800 polymorphism and NIHL (Homozygote model: TT versus CC). Figure K in S3 File: Begg’s funnel plot of association between the rs2075800 polymorphism and NIHL (Dominant model: TT+CT versus CC). Figure L in S3 File: Begg’s funnel plot of association between the rs2075800 polymorphism and NIHL (Recessive model: TT versus CT+CC). Figure M in S3 File: Begg’s funnel plot of association between the rs2227956 polymorphism and NIHL (Heterozygote model: TC versus TT). Figure N in S3 File: Begg’s funnel plot of association between the rs2227956 polymorphism and NIHL (Homozygote model: CC versus TT). Figure O in S3 File: Begg’s funnel plot of association between the rs22279 [file pone.0188539.s003.zip › S3 File/Figure G.tif]

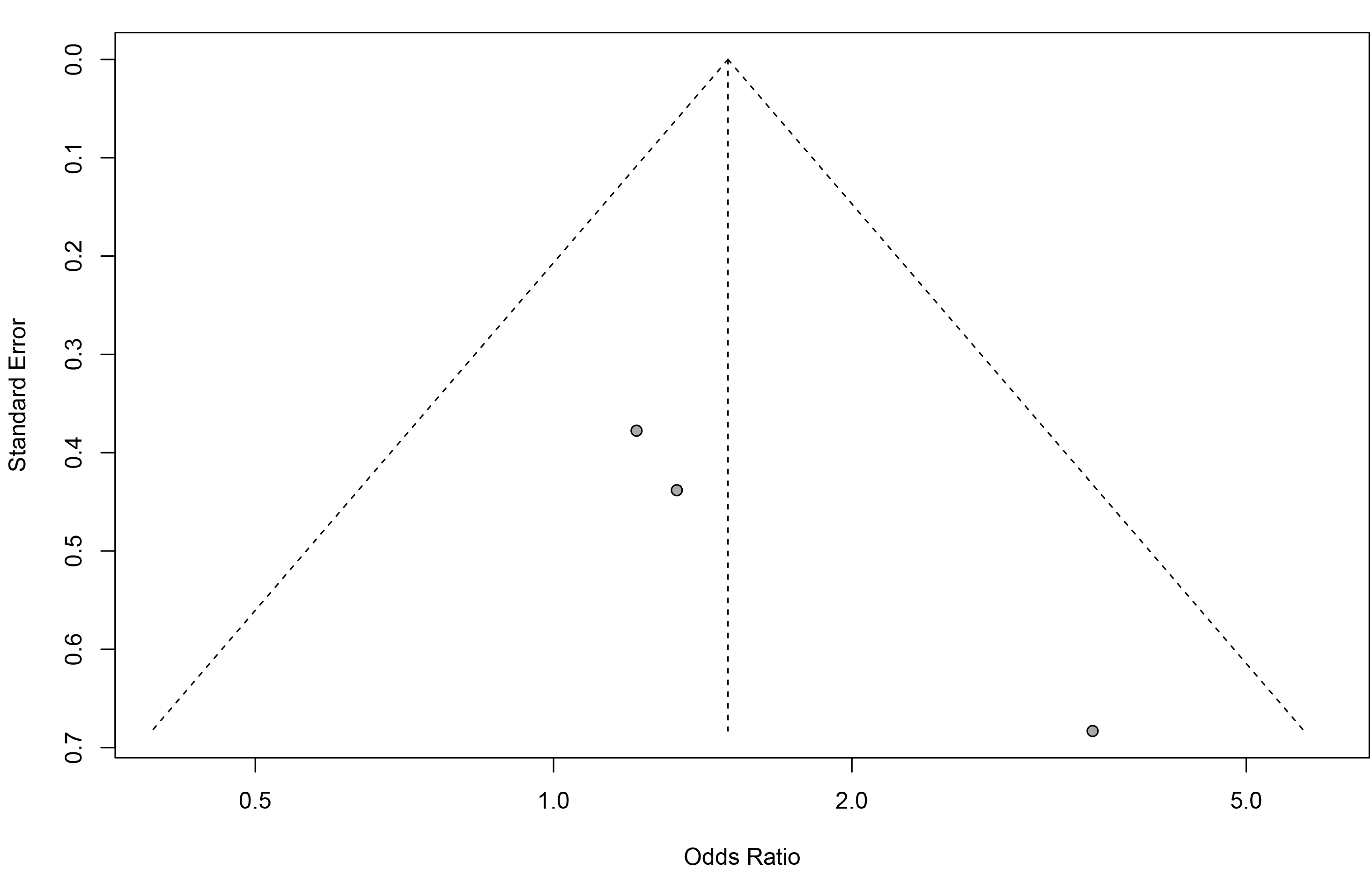

Supplement: S3 File — Begg’s funnel plot (Figure A—Figure T). (ZIP). Figure A in S3 File: Begg’s funnel plot of association between the rs1043618 polymorphism and NIHL (Heterozygote model: GC versus GG). Figure B in S3 File: Begg’s funnel plot of association between the rs1043618 polymorphism and NIHL (Homozygote model: CC versus GG). Figure C in S3 File: Begg’s funnel plot of association between the rs1043618 polymorphism and NIHL (Dominant model: CC+GC versus GG). Figure D in S3 File: Begg’s funnel plot of association between the rs1043618 polymorphism and NIHL (Recessive model: CC versus CG+GG). Figure E in S3 File: Begg’s funnel plot of association between the rs1061581 polymorphism and NIHL (Heterozygote model: AG versus AA). Figure F in S3 File: Begg’s funnel plot of association between the rs1061581 polymorphism and NIHL (Homozygote model: GG versus AA). Figure G in S3 File: Begg’s funnel plot of association between the rs1061581 polymorphism and NIHL (Dominant model: GG+AG versus AA). Figure H in S3 File: Begg’s funnel plot of association between the rs1061581 polymorphism and NIHL (Recessive model: GG versus AG+AA). Figure I in S3 File: Begg’s funnel plot of association between the rs2075800 polymorphism and NIHL (Heterozygote model: CT versus CC). Figure J in S3 File: Begg’s funnel plot of association between the rs2075800 polymorphism and NIHL (Homozygote model: TT versus CC). Figure K in S3 File: Begg’s funnel plot of association between the rs2075800 polymorphism and NIHL (Dominant model: TT+CT versus CC). Figure L in S3 File: Begg’s funnel plot of association between the rs2075800 polymorphism and NIHL (Recessive model: TT versus CT+CC). Figure M in S3 File: Begg’s funnel plot of association between the rs2227956 polymorphism and NIHL (Heterozygote model: TC versus TT). Figure N in S3 File: Begg’s funnel plot of association between the rs2227956 polymorphism and NIHL (Homozygote model: CC versus TT). Figure O in S3 File: Begg’s funnel plot of association between the rs22279 [file pone.0188539.s003.zip › S3 File/Figure H.tif]

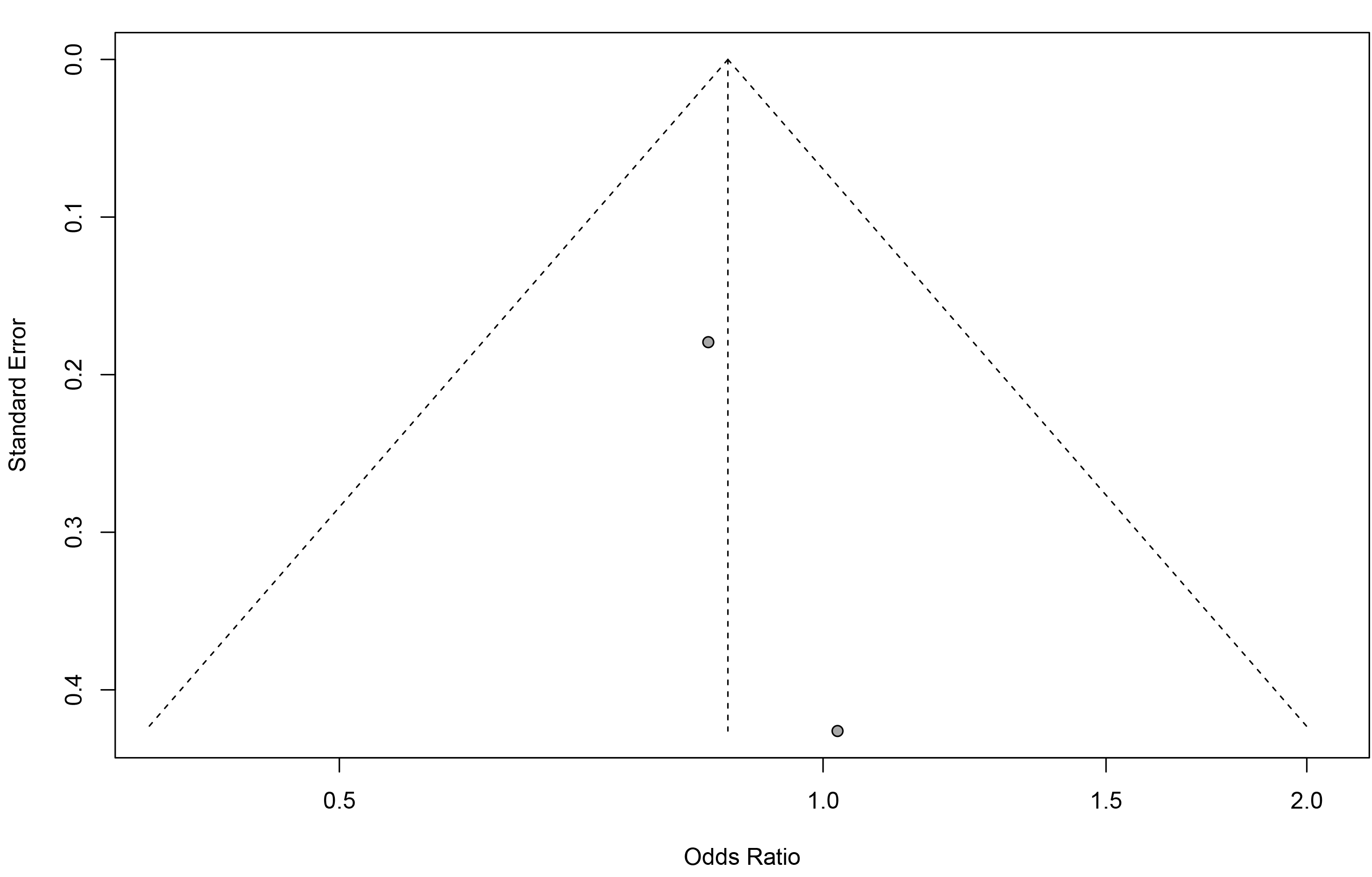

Supplement: S3 File — Begg’s funnel plot (Figure A—Figure T). (ZIP). Figure A in S3 File: Begg’s funnel plot of association between the rs1043618 polymorphism and NIHL (Heterozygote model: GC versus GG). Figure B in S3 File: Begg’s funnel plot of association between the rs1043618 polymorphism and NIHL (Homozygote model: CC versus GG). Figure C in S3 File: Begg’s funnel plot of association between the rs1043618 polymorphism and NIHL (Dominant model: CC+GC versus GG). Figure D in S3 File: Begg’s funnel plot of association between the rs1043618 polymorphism and NIHL (Recessive model: CC versus CG+GG). Figure E in S3 File: Begg’s funnel plot of association between the rs1061581 polymorphism and NIHL (Heterozygote model: AG versus AA). Figure F in S3 File: Begg’s funnel plot of association between the rs1061581 polymorphism and NIHL (Homozygote model: GG versus AA). Figure G in S3 File: Begg’s funnel plot of association between the rs1061581 polymorphism and NIHL (Dominant model: GG+AG versus AA). Figure H in S3 File: Begg’s funnel plot of association between the rs1061581 polymorphism and NIHL (Recessive model: GG versus AG+AA). Figure I in S3 File: Begg’s funnel plot of association between the rs2075800 polymorphism and NIHL (Heterozygote model: CT versus CC). Figure J in S3 File: Begg’s funnel plot of association between the rs2075800 polymorphism and NIHL (Homozygote model: TT versus CC). Figure K in S3 File: Begg’s funnel plot of association between the rs2075800 polymorphism and NIHL (Dominant model: TT+CT versus CC). Figure L in S3 File: Begg’s funnel plot of association between the rs2075800 polymorphism and NIHL (Recessive model: TT versus CT+CC). Figure M in S3 File: Begg’s funnel plot of association between the rs2227956 polymorphism and NIHL (Heterozygote model: TC versus TT). Figure N in S3 File: Begg’s funnel plot of association between the rs2227956 polymorphism and NIHL (Homozygote model: CC versus TT). Figure O in S3 File: Begg’s funnel plot of association between the rs22279 [file pone.0188539.s003.zip › S3 File/Figure I.tif]

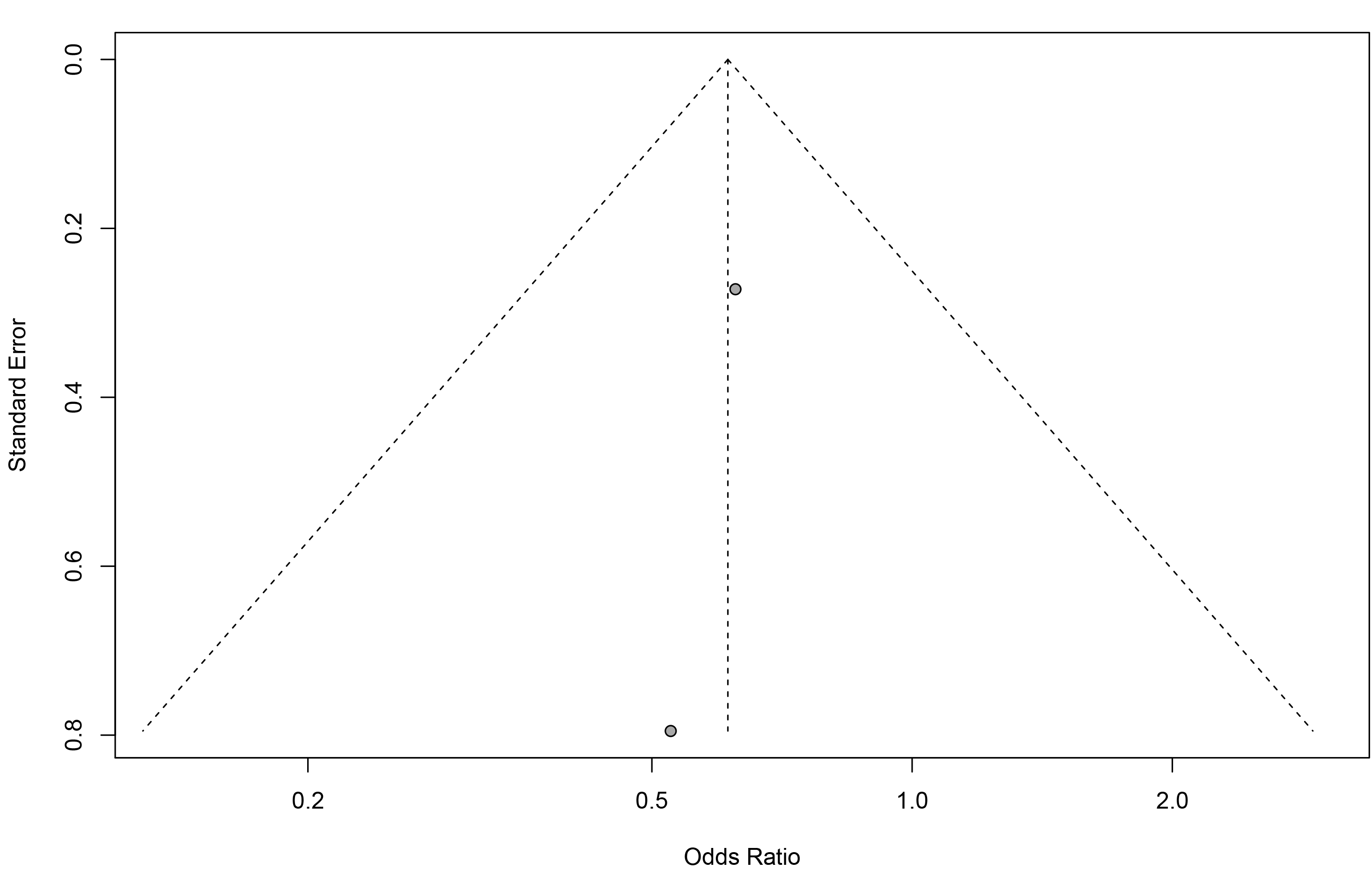

Supplement: S3 File — Begg’s funnel plot (Figure A—Figure T). (ZIP). Figure A in S3 File: Begg’s funnel plot of association between the rs1043618 polymorphism and NIHL (Heterozygote model: GC versus GG). Figure B in S3 File: Begg’s funnel plot of association between the rs1043618 polymorphism and NIHL (Homozygote model: CC versus GG). Figure C in S3 File: Begg’s funnel plot of association between the rs1043618 polymorphism and NIHL (Dominant model: CC+GC versus GG). Figure D in S3 File: Begg’s funnel plot of association between the rs1043618 polymorphism and NIHL (Recessive model: CC versus CG+GG). Figure E in S3 File: Begg’s funnel plot of association between the rs1061581 polymorphism and NIHL (Heterozygote model: AG versus AA). Figure F in S3 File: Begg’s funnel plot of association between the rs1061581 polymorphism and NIHL (Homozygote model: GG versus AA). Figure G in S3 File: Begg’s funnel plot of association between the rs1061581 polymorphism and NIHL (Dominant model: GG+AG versus AA). Figure H in S3 File: Begg’s funnel plot of association between the rs1061581 polymorphism and NIHL (Recessive model: GG versus AG+AA). Figure I in S3 File: Begg’s funnel plot of association between the rs2075800 polymorphism and NIHL (Heterozygote model: CT versus CC). Figure J in S3 File: Begg’s funnel plot of association between the rs2075800 polymorphism and NIHL (Homozygote model: TT versus CC). Figure K in S3 File: Begg’s funnel plot of association between the rs2075800 polymorphism and NIHL (Dominant model: TT+CT versus CC). Figure L in S3 File: Begg’s funnel plot of association between the rs2075800 polymorphism and NIHL (Recessive model: TT versus CT+CC). Figure M in S3 File: Begg’s funnel plot of association between the rs2227956 polymorphism and NIHL (Heterozygote model: TC versus TT). Figure N in S3 File: Begg’s funnel plot of association between the rs2227956 polymorphism and NIHL (Homozygote model: CC versus TT). Figure O in S3 File: Begg’s funnel plot of association between the rs22279 [file pone.0188539.s003.zip › S3 File/Figure J.tif]

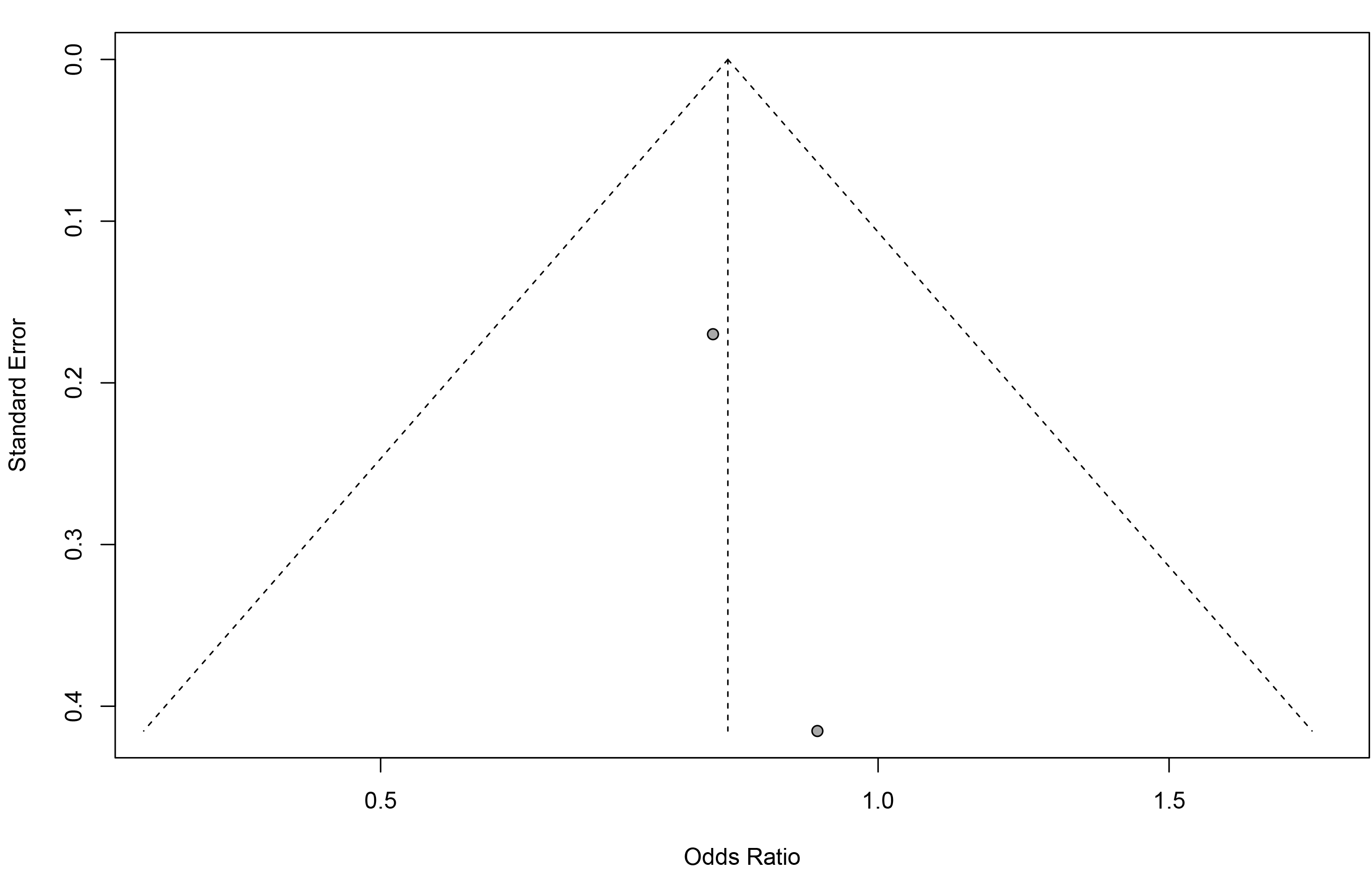

Supplement: S3 File — Begg’s funnel plot (Figure A—Figure T). (ZIP). Figure A in S3 File: Begg’s funnel plot of association between the rs1043618 polymorphism and NIHL (Heterozygote model: GC versus GG). Figure B in S3 File: Begg’s funnel plot of association between the rs1043618 polymorphism and NIHL (Homozygote model: CC versus GG). Figure C in S3 File: Begg’s funnel plot of association between the rs1043618 polymorphism and NIHL (Dominant model: CC+GC versus GG). Figure D in S3 File: Begg’s funnel plot of association between the rs1043618 polymorphism and NIHL (Recessive model: CC versus CG+GG). Figure E in S3 File: Begg’s funnel plot of association between the rs1061581 polymorphism and NIHL (Heterozygote model: AG versus AA). Figure F in S3 File: Begg’s funnel plot of association between the rs1061581 polymorphism and NIHL (Homozygote model: GG versus AA). Figure G in S3 File: Begg’s funnel plot of association between the rs1061581 polymorphism and NIHL (Dominant model: GG+AG versus AA). Figure H in S3 File: Begg’s funnel plot of association between the rs1061581 polymorphism and NIHL (Recessive model: GG versus AG+AA). Figure I in S3 File: Begg’s funnel plot of association between the rs2075800 polymorphism and NIHL (Heterozygote model: CT versus CC). Figure J in S3 File: Begg’s funnel plot of association between the rs2075800 polymorphism and NIHL (Homozygote model: TT versus CC). Figure K in S3 File: Begg’s funnel plot of association between the rs2075800 polymorphism and NIHL (Dominant model: TT+CT versus CC). Figure L in S3 File: Begg’s funnel plot of association between the rs2075800 polymorphism and NIHL (Recessive model: TT versus CT+CC). Figure M in S3 File: Begg’s funnel plot of association between the rs2227956 polymorphism and NIHL (Heterozygote model: TC versus TT). Figure N in S3 File: Begg’s funnel plot of association between the rs2227956 polymorphism and NIHL (Homozygote model: CC versus TT). Figure O in S3 File: Begg’s funnel plot of association between the rs22279 [file pone.0188539.s003.zip › S3 File/Figure K.tif]

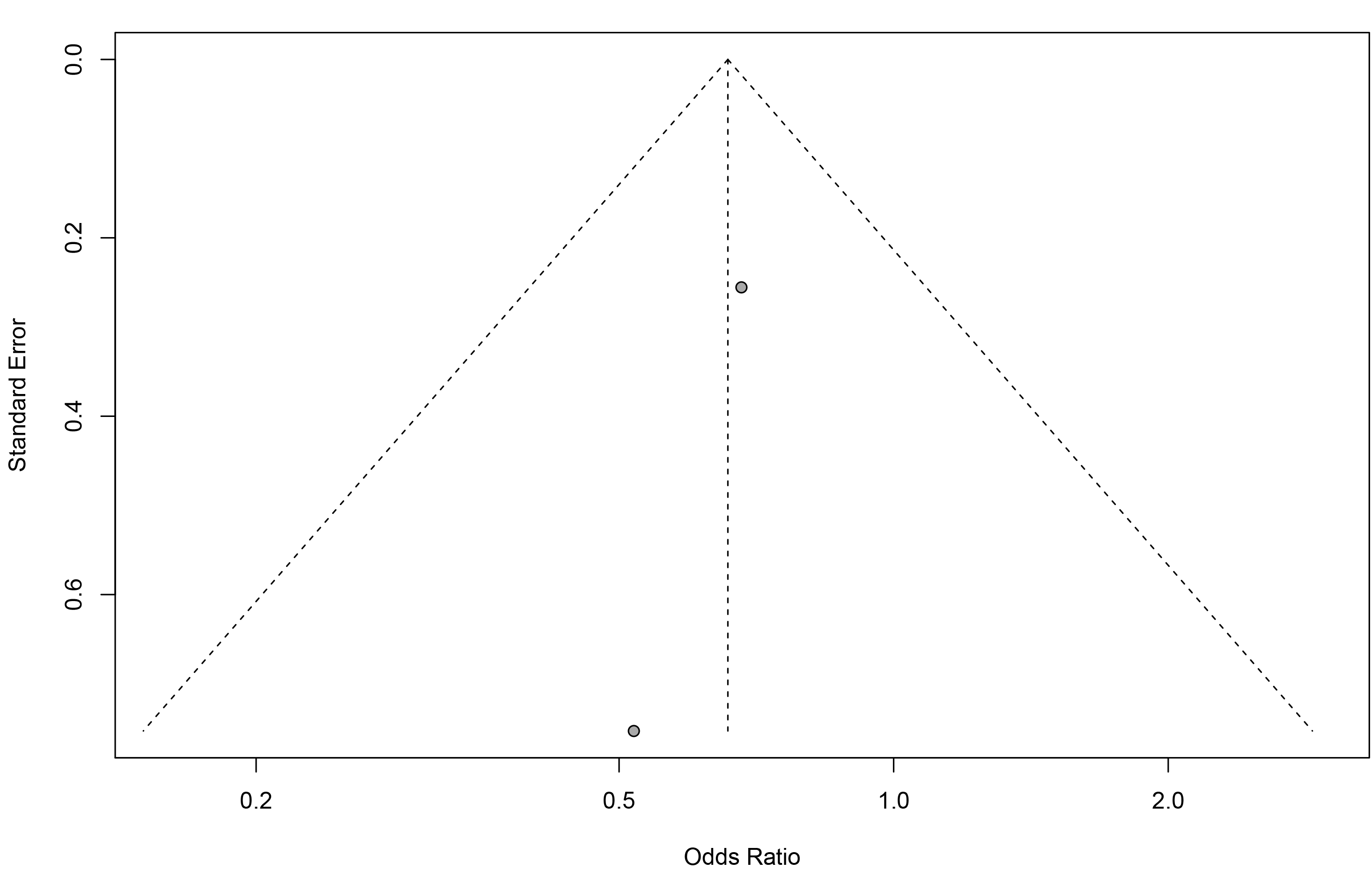

Supplement: S3 File — Begg’s funnel plot (Figure A—Figure T). (ZIP). Figure A in S3 File: Begg’s funnel plot of association between the rs1043618 polymorphism and NIHL (Heterozygote model: GC versus GG). Figure B in S3 File: Begg’s funnel plot of association between the rs1043618 polymorphism and NIHL (Homozygote model: CC versus GG). Figure C in S3 File: Begg’s funnel plot of association between the rs1043618 polymorphism and NIHL (Dominant model: CC+GC versus GG). Figure D in S3 File: Begg’s funnel plot of association between the rs1043618 polymorphism and NIHL (Recessive model: CC versus CG+GG). Figure E in S3 File: Begg’s funnel plot of association between the rs1061581 polymorphism and NIHL (Heterozygote model: AG versus AA). Figure F in S3 File: Begg’s funnel plot of association between the rs1061581 polymorphism and NIHL (Homozygote model: GG versus AA). Figure G in S3 File: Begg’s funnel plot of association between the rs1061581 polymorphism and NIHL (Dominant model: GG+AG versus AA). Figure H in S3 File: Begg’s funnel plot of association between the rs1061581 polymorphism and NIHL (Recessive model: GG versus AG+AA). Figure I in S3 File: Begg’s funnel plot of association between the rs2075800 polymorphism and NIHL (Heterozygote model: CT versus CC). Figure J in S3 File: Begg’s funnel plot of association between the rs2075800 polymorphism and NIHL (Homozygote model: TT versus CC). Figure K in S3 File: Begg’s funnel plot of association between the rs2075800 polymorphism and NIHL (Dominant model: TT+CT versus CC). Figure L in S3 File: Begg’s funnel plot of association between the rs2075800 polymorphism and NIHL (Recessive model: TT versus CT+CC). Figure M in S3 File: Begg’s funnel plot of association between the rs2227956 polymorphism and NIHL (Heterozygote model: TC versus TT). Figure N in S3 File: Begg’s funnel plot of association between the rs2227956 polymorphism and NIHL (Homozygote model: CC versus TT). Figure O in S3 File: Begg’s funnel plot of association between the rs22279 [file pone.0188539.s003.zip › S3 File/Figure L.tif]

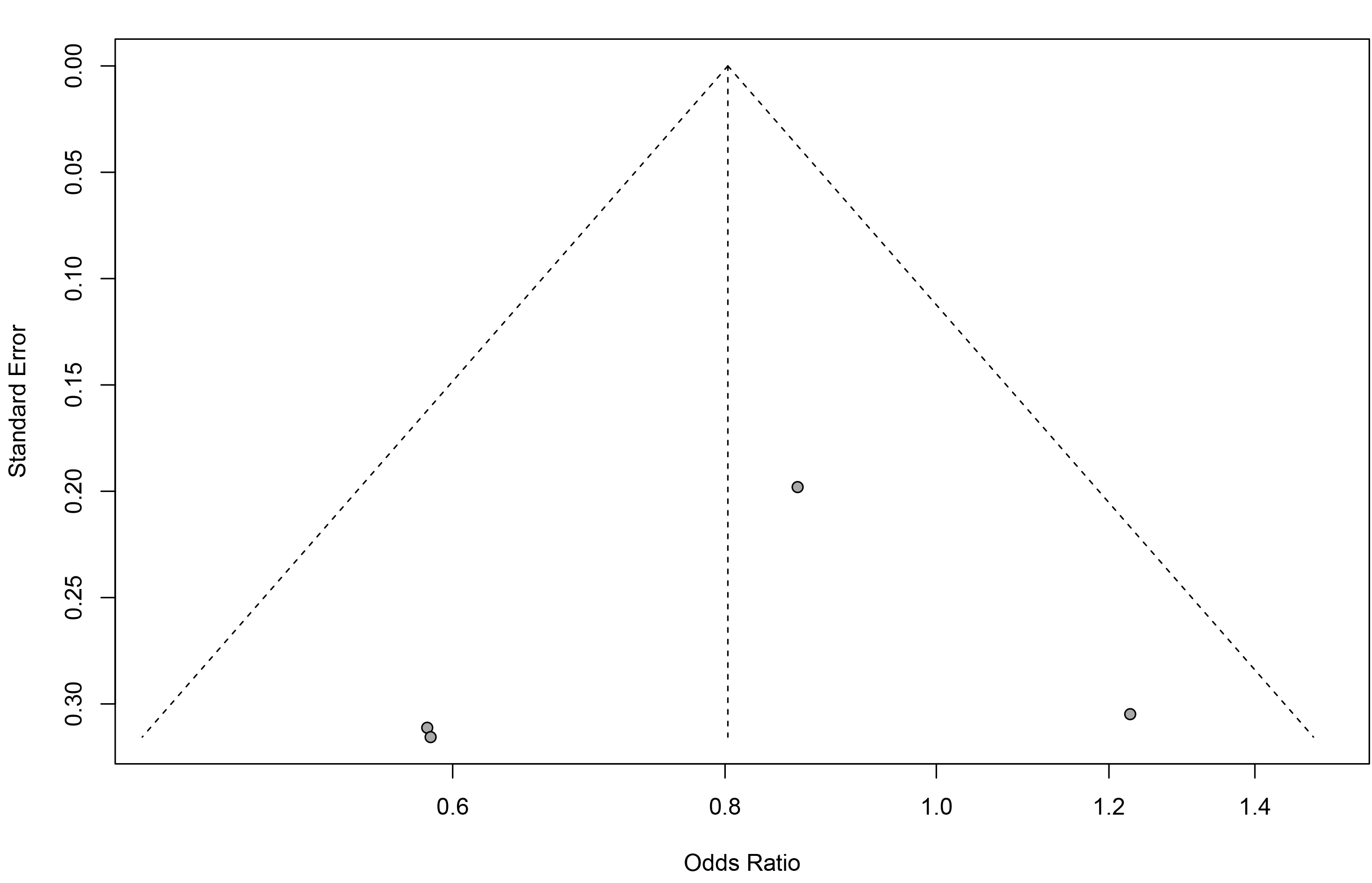

Supplement: S3 File — Begg’s funnel plot (Figure A—Figure T). (ZIP). Figure A in S3 File: Begg’s funnel plot of association between the rs1043618 polymorphism and NIHL (Heterozygote model: GC versus GG). Figure B in S3 File: Begg’s funnel plot of association between the rs1043618 polymorphism and NIHL (Homozygote model: CC versus GG). Figure C in S3 File: Begg’s funnel plot of association between the rs1043618 polymorphism and NIHL (Dominant model: CC+GC versus GG). Figure D in S3 File: Begg’s funnel plot of association between the rs1043618 polymorphism and NIHL (Recessive model: CC versus CG+GG). Figure E in S3 File: Begg’s funnel plot of association between the rs1061581 polymorphism and NIHL (Heterozygote model: AG versus AA). Figure F in S3 File: Begg’s funnel plot of association between the rs1061581 polymorphism and NIHL (Homozygote model: GG versus AA). Figure G in S3 File: Begg’s funnel plot of association between the rs1061581 polymorphism and NIHL (Dominant model: GG+AG versus AA). Figure H in S3 File: Begg’s funnel plot of association between the rs1061581 polymorphism and NIHL (Recessive model: GG versus AG+AA). Figure I in S3 File: Begg’s funnel plot of association between the rs2075800 polymorphism and NIHL (Heterozygote model: CT versus CC). Figure J in S3 File: Begg’s funnel plot of association between the rs2075800 polymorphism and NIHL (Homozygote model: TT versus CC). Figure K in S3 File: Begg’s funnel plot of association between the rs2075800 polymorphism and NIHL (Dominant model: TT+CT versus CC). Figure L in S3 File: Begg’s funnel plot of association between the rs2075800 polymorphism and NIHL (Recessive model: TT versus CT+CC). Figure M in S3 File: Begg’s funnel plot of association between the rs2227956 polymorphism and NIHL (Heterozygote model: TC versus TT). Figure N in S3 File: Begg’s funnel plot of association between the rs2227956 polymorphism and NIHL (Homozygote model: CC versus TT). Figure O in S3 File: Begg’s funnel plot of association between the rs22279 [file pone.0188539.s003.zip › S3 File/Figure M.tif]

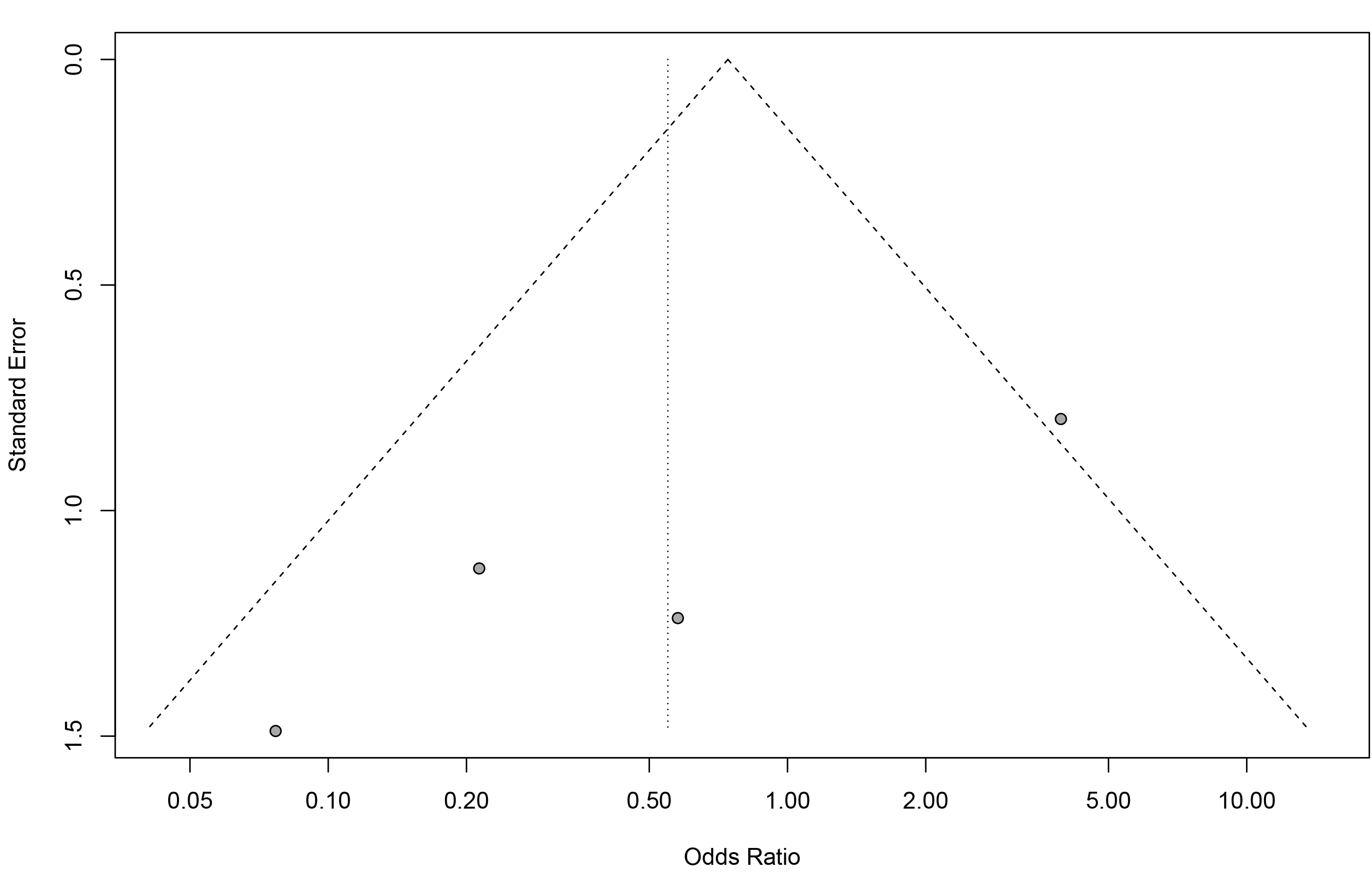

Supplement: S3 File — Begg’s funnel plot (Figure A—Figure T). (ZIP). Figure A in S3 File: Begg’s funnel plot of association between the rs1043618 polymorphism and NIHL (Heterozygote model: GC versus GG). Figure B in S3 File: Begg’s funnel plot of association between the rs1043618 polymorphism and NIHL (Homozygote model: CC versus GG). Figure C in S3 File: Begg’s funnel plot of association between the rs1043618 polymorphism and NIHL (Dominant model: CC+GC versus GG). Figure D in S3 File: Begg’s funnel plot of association between the rs1043618 polymorphism and NIHL (Recessive model: CC versus CG+GG). Figure E in S3 File: Begg’s funnel plot of association between the rs1061581 polymorphism and NIHL (Heterozygote model: AG versus AA). Figure F in S3 File: Begg’s funnel plot of association between the rs1061581 polymorphism and NIHL (Homozygote model: GG versus AA). Figure G in S3 File: Begg’s funnel plot of association between the rs1061581 polymorphism and NIHL (Dominant model: GG+AG versus AA). Figure H in S3 File: Begg’s funnel plot of association between the rs1061581 polymorphism and NIHL (Recessive model: GG versus AG+AA). Figure I in S3 File: Begg’s funnel plot of association between the rs2075800 polymorphism and NIHL (Heterozygote model: CT versus CC). Figure J in S3 File: Begg’s funnel plot of association between the rs2075800 polymorphism and NIHL (Homozygote model: TT versus CC). Figure K in S3 File: Begg’s funnel plot of association between the rs2075800 polymorphism and NIHL (Dominant model: TT+CT versus CC). Figure L in S3 File: Begg’s funnel plot of association between the rs2075800 polymorphism and NIHL (Recessive model: TT versus CT+CC). Figure M in S3 File: Begg’s funnel plot of association between the rs2227956 polymorphism and NIHL (Heterozygote model: TC versus TT). Figure N in S3 File: Begg’s funnel plot of association between the rs2227956 polymorphism and NIHL (Homozygote model: CC versus TT). Figure O in S3 File: Begg’s funnel plot of association between the rs22279 [file pone.0188539.s003.zip › S3 File/Figure N.tif]

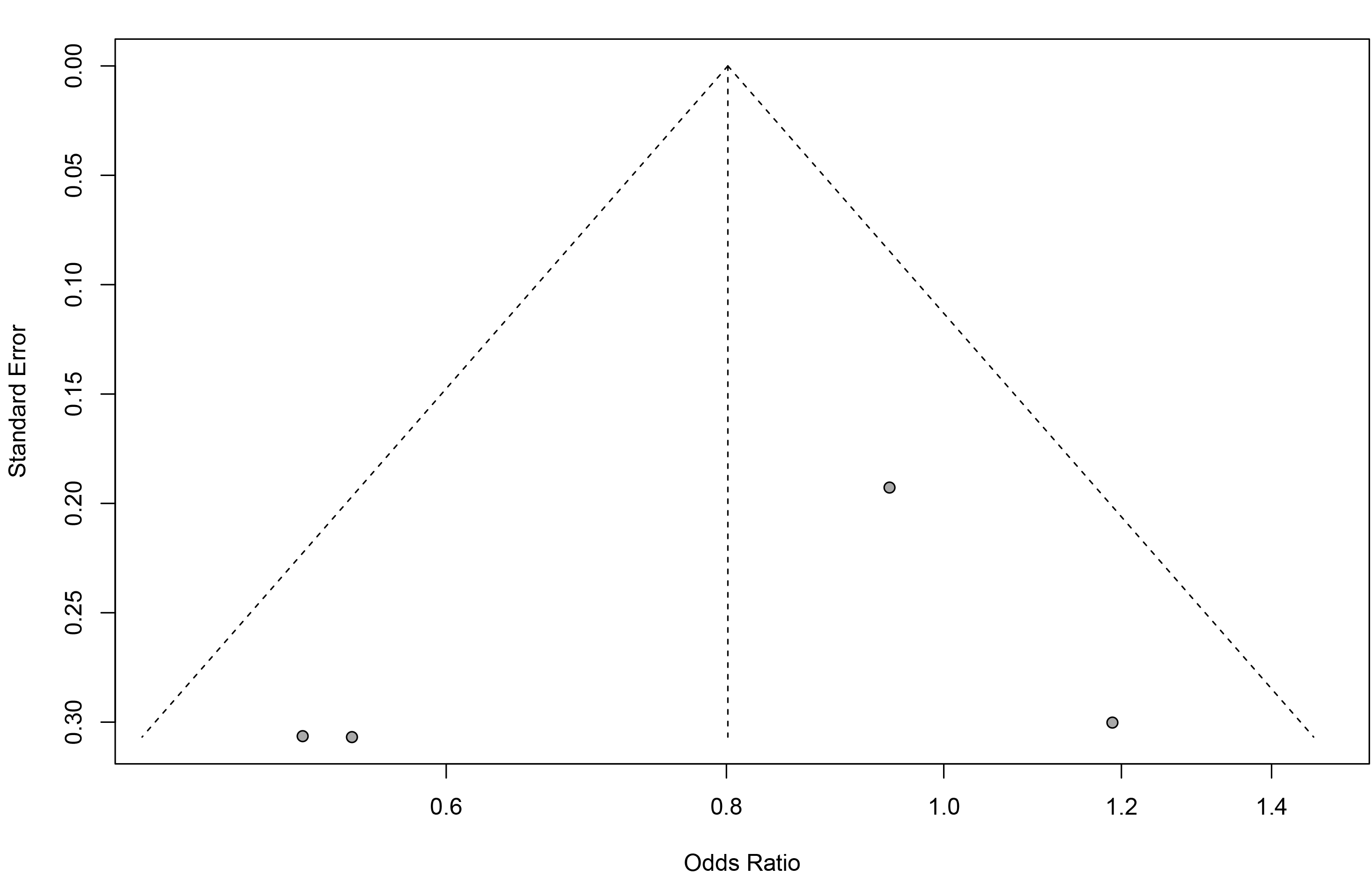

Supplement: S3 File — Begg’s funnel plot (Figure A—Figure T). (ZIP). Figure A in S3 File: Begg’s funnel plot of association between the rs1043618 polymorphism and NIHL (Heterozygote model: GC versus GG). Figure B in S3 File: Begg’s funnel plot of association between the rs1043618 polymorphism and NIHL (Homozygote model: CC versus GG). Figure C in S3 File: Begg’s funnel plot of association between the rs1043618 polymorphism and NIHL (Dominant model: CC+GC versus GG). Figure D in S3 File: Begg’s funnel plot of association between the rs1043618 polymorphism and NIHL (Recessive model: CC versus CG+GG). Figure E in S3 File: Begg’s funnel plot of association between the rs1061581 polymorphism and NIHL (Heterozygote model: AG versus AA). Figure F in S3 File: Begg’s funnel plot of association between the rs1061581 polymorphism and NIHL (Homozygote model: GG versus AA). Figure G in S3 File: Begg’s funnel plot of association between the rs1061581 polymorphism and NIHL (Dominant model: GG+AG versus AA). Figure H in S3 File: Begg’s funnel plot of association between the rs1061581 polymorphism and NIHL (Recessive model: GG versus AG+AA). Figure I in S3 File: Begg’s funnel plot of association between the rs2075800 polymorphism and NIHL (Heterozygote model: CT versus CC). Figure J in S3 File: Begg’s funnel plot of association between the rs2075800 polymorphism and NIHL (Homozygote model: TT versus CC). Figure K in S3 File: Begg’s funnel plot of association between the rs2075800 polymorphism and NIHL (Dominant model: TT+CT versus CC). Figure L in S3 File: Begg’s funnel plot of association between the rs2075800 polymorphism and NIHL (Recessive model: TT versus CT+CC). Figure M in S3 File: Begg’s funnel plot of association between the rs2227956 polymorphism and NIHL (Heterozygote model: TC versus TT). Figure N in S3 File: Begg’s funnel plot of association between the rs2227956 polymorphism and NIHL (Homozygote model: CC versus TT). Figure O in S3 File: Begg’s funnel plot of association between the rs22279 [file pone.0188539.s003.zip › S3 File/Figure O.tif]

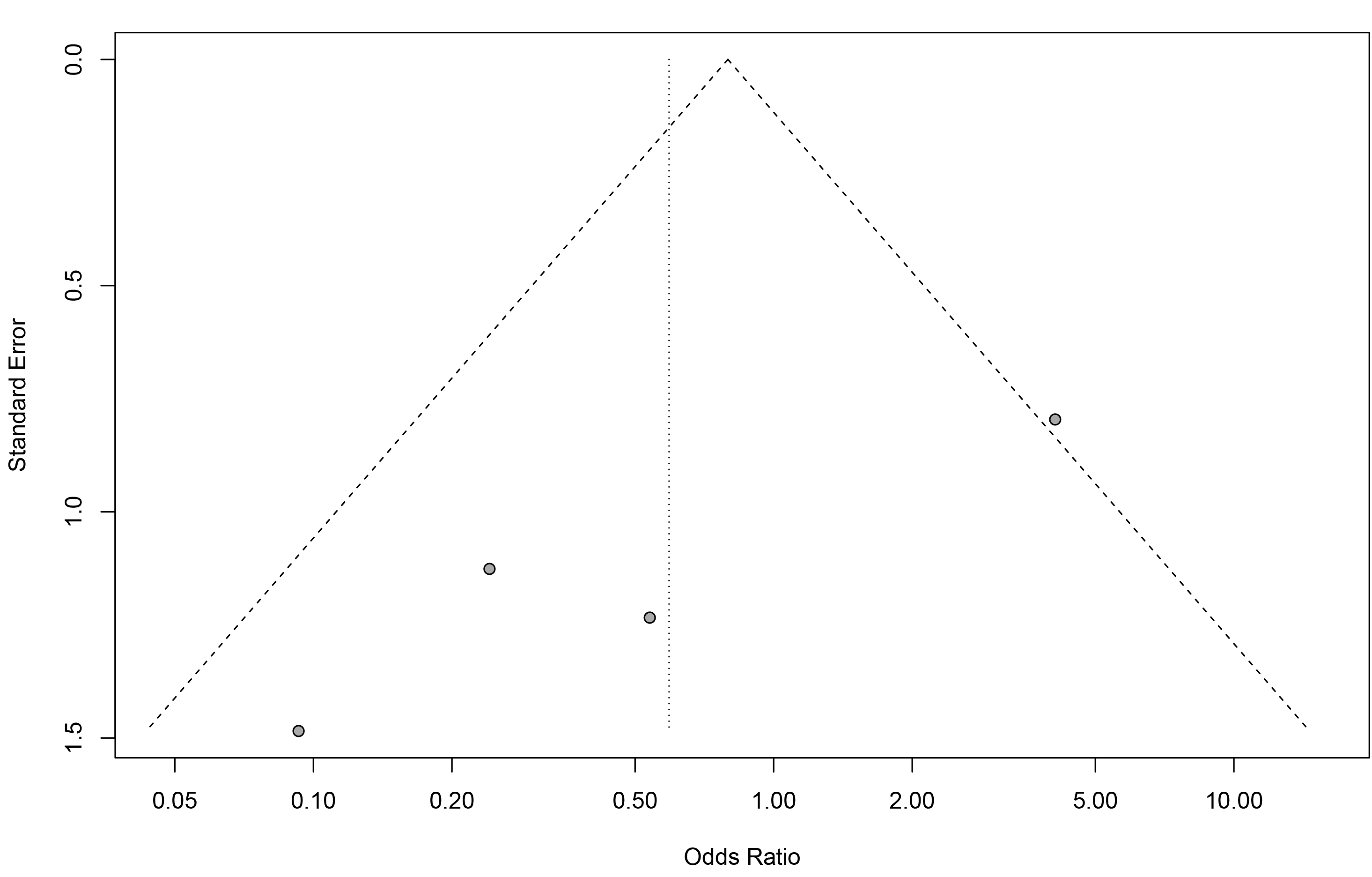

Supplement: S3 File — Begg’s funnel plot (Figure A—Figure T). (ZIP). Figure A in S3 File: Begg’s funnel plot of association between the rs1043618 polymorphism and NIHL (Heterozygote model: GC versus GG). Figure B in S3 File: Begg’s funnel plot of association between the rs1043618 polymorphism and NIHL (Homozygote model: CC versus GG). Figure C in S3 File: Begg’s funnel plot of association between the rs1043618 polymorphism and NIHL (Dominant model: CC+GC versus GG). Figure D in S3 File: Begg’s funnel plot of association between the rs1043618 polymorphism and NIHL (Recessive model: CC versus CG+GG). Figure E in S3 File: Begg’s funnel plot of association between the rs1061581 polymorphism and NIHL (Heterozygote model: AG versus AA). Figure F in S3 File: Begg’s funnel plot of association between the rs1061581 polymorphism and NIHL (Homozygote model: GG versus AA). Figure G in S3 File: Begg’s funnel plot of association between the rs1061581 polymorphism and NIHL (Dominant model: GG+AG versus AA). Figure H in S3 File: Begg’s funnel plot of association between the rs1061581 polymorphism and NIHL (Recessive model: GG versus AG+AA). Figure I in S3 File: Begg’s funnel plot of association between the rs2075800 polymorphism and NIHL (Heterozygote model: CT versus CC). Figure J in S3 File: Begg’s funnel plot of association between the rs2075800 polymorphism and NIHL (Homozygote model: TT versus CC). Figure K in S3 File: Begg’s funnel plot of association between the rs2075800 polymorphism and NIHL (Dominant model: TT+CT versus CC). Figure L in S3 File: Begg’s funnel plot of association between the rs2075800 polymorphism and NIHL (Recessive model: TT versus CT+CC). Figure M in S3 File: Begg’s funnel plot of association between the rs2227956 polymorphism and NIHL (Heterozygote model: TC versus TT). Figure N in S3 File: Begg’s funnel plot of association between the rs2227956 polymorphism and NIHL (Homozygote model: CC versus TT). Figure O in S3 File: Begg’s funnel plot of association between the rs22279 [file pone.0188539.s003.zip › S3 File/Figure P.tif]

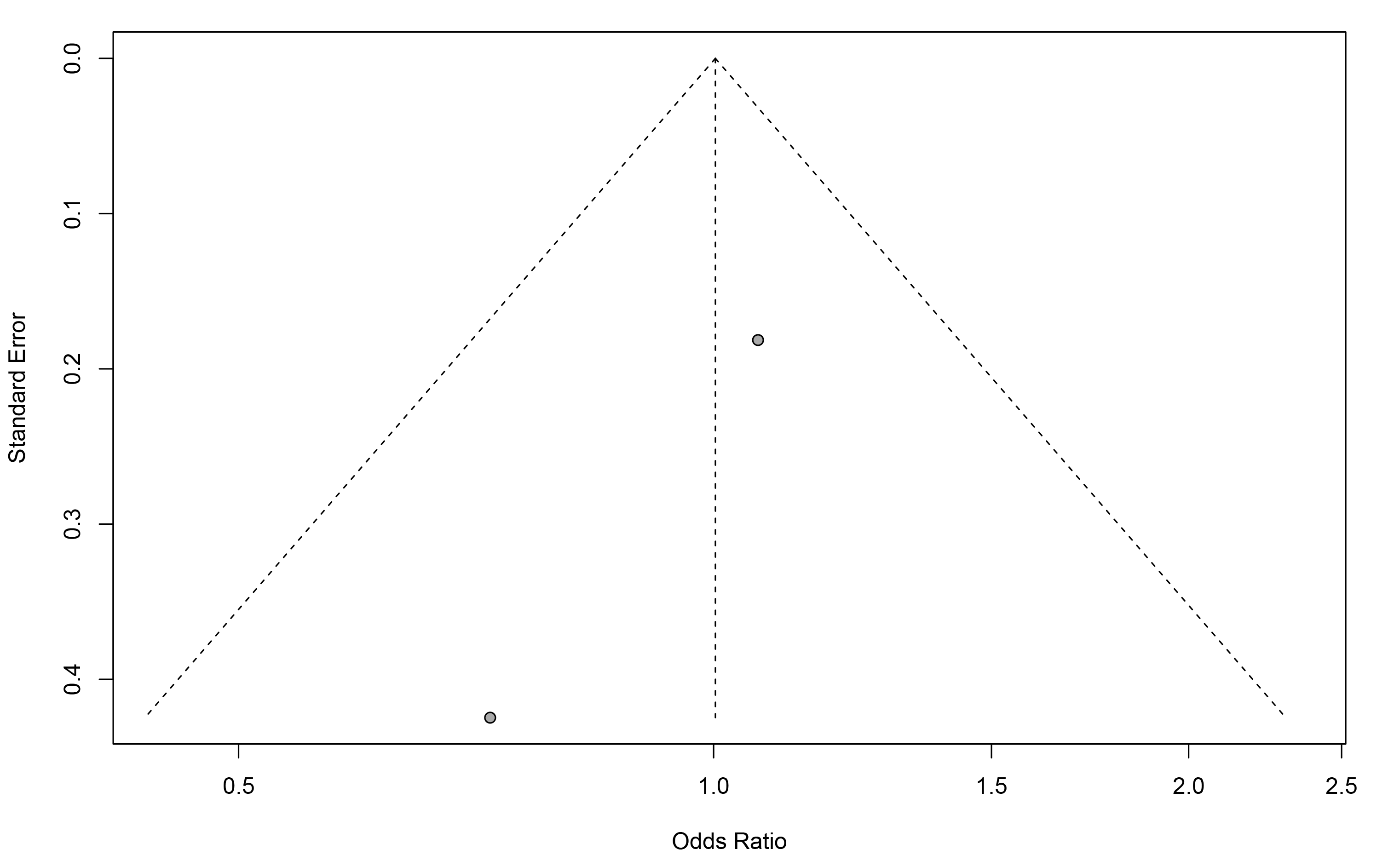

Supplement: S3 File — Begg’s funnel plot (Figure A—Figure T). (ZIP). Figure A in S3 File: Begg’s funnel plot of association between the rs1043618 polymorphism and NIHL (Heterozygote model: GC versus GG). Figure B in S3 File: Begg’s funnel plot of association between the rs1043618 polymorphism and NIHL (Homozygote model: CC versus GG). Figure C in S3 File: Begg’s funnel plot of association between the rs1043618 polymorphism and NIHL (Dominant model: CC+GC versus GG). Figure D in S3 File: Begg’s funnel plot of association between the rs1043618 polymorphism and NIHL (Recessive model: CC versus CG+GG). Figure E in S3 File: Begg’s funnel plot of association between the rs1061581 polymorphism and NIHL (Heterozygote model: AG versus AA). Figure F in S3 File: Begg’s funnel plot of association between the rs1061581 polymorphism and NIHL (Homozygote model: GG versus AA). Figure G in S3 File: Begg’s funnel plot of association between the rs1061581 polymorphism and NIHL (Dominant model: GG+AG versus AA). Figure H in S3 File: Begg’s funnel plot of association between the rs1061581 polymorphism and NIHL (Recessive model: GG versus AG+AA). Figure I in S3 File: Begg’s funnel plot of association between the rs2075800 polymorphism and NIHL (Heterozygote model: CT versus CC). Figure J in S3 File: Begg’s funnel plot of association between the rs2075800 polymorphism and NIHL (Homozygote model: TT versus CC). Figure K in S3 File: Begg’s funnel plot of association between the rs2075800 polymorphism and NIHL (Dominant model: TT+CT versus CC). Figure L in S3 File: Begg’s funnel plot of association between the rs2075800 polymorphism and NIHL (Recessive model: TT versus CT+CC). Figure M in S3 File: Begg’s funnel plot of association between the rs2227956 polymorphism and NIHL (Heterozygote model: TC versus TT). Figure N in S3 File: Begg’s funnel plot of association between the rs2227956 polymorphism and NIHL (Homozygote model: CC versus TT). Figure O in S3 File: Begg’s funnel plot of association between the rs22279 [file pone.0188539.s003.zip › S3 File/Figure Q.tif]

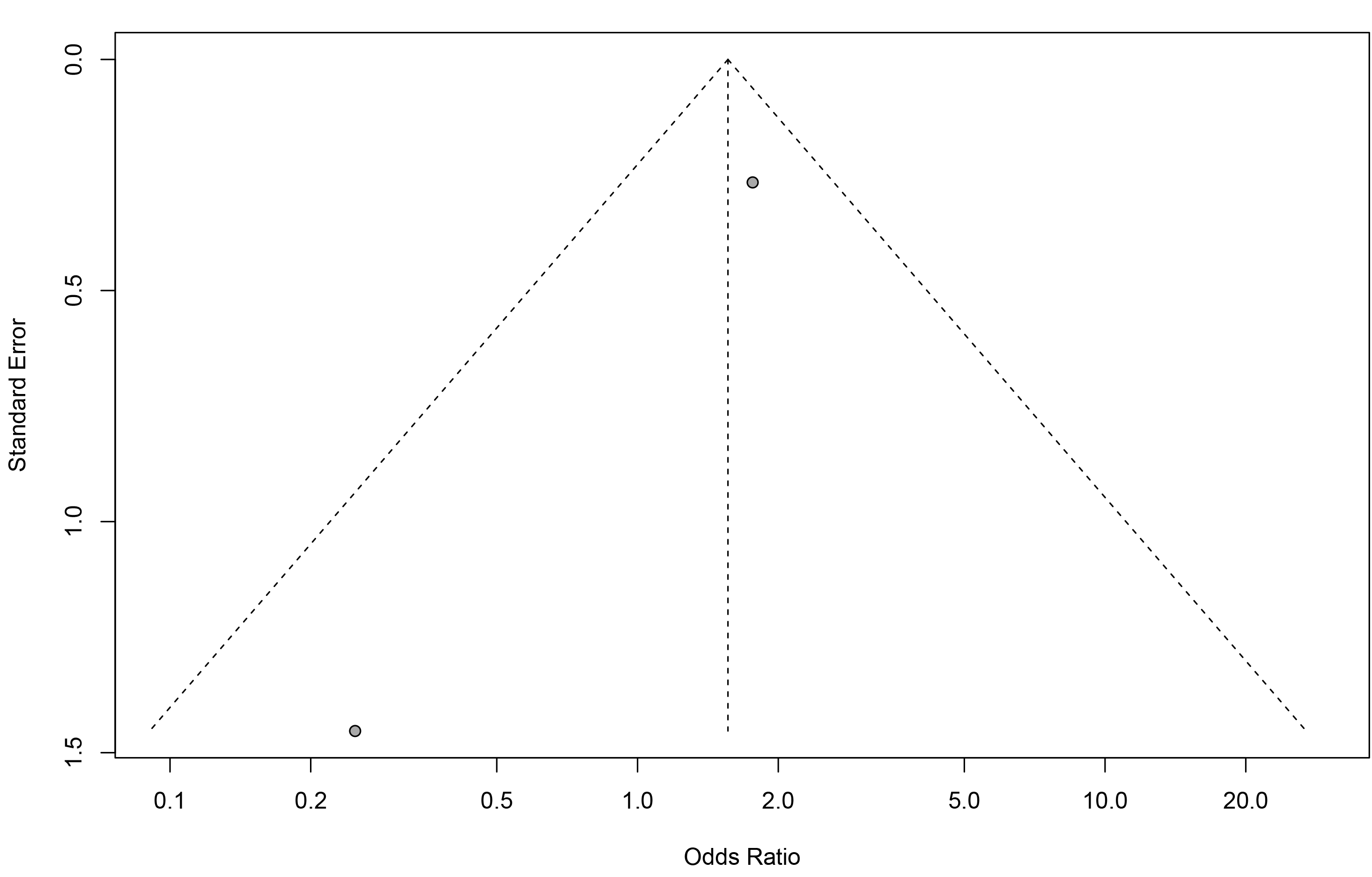

Supplement: S3 File — Begg’s funnel plot (Figure A—Figure T). (ZIP). Figure A in S3 File: Begg’s funnel plot of association between the rs1043618 polymorphism and NIHL (Heterozygote model: GC versus GG). Figure B in S3 File: Begg’s funnel plot of association between the rs1043618 polymorphism and NIHL (Homozygote model: CC versus GG). Figure C in S3 File: Begg’s funnel plot of association between the rs1043618 polymorphism and NIHL (Dominant model: CC+GC versus GG). Figure D in S3 File: Begg’s funnel plot of association between the rs1043618 polymorphism and NIHL (Recessive model: CC versus CG+GG). Figure E in S3 File: Begg’s funnel plot of association between the rs1061581 polymorphism and NIHL (Heterozygote model: AG versus AA). Figure F in S3 File: Begg’s funnel plot of association between the rs1061581 polymorphism and NIHL (Homozygote model: GG versus AA). Figure G in S3 File: Begg’s funnel plot of association between the rs1061581 polymorphism and NIHL (Dominant model: GG+AG versus AA). Figure H in S3 File: Begg’s funnel plot of association between the rs1061581 polymorphism and NIHL (Recessive model: GG versus AG+AA). Figure I in S3 File: Begg’s funnel plot of association between the rs2075800 polymorphism and NIHL (Heterozygote model: CT versus CC). Figure J in S3 File: Begg’s funnel plot of association between the rs2075800 polymorphism and NIHL (Homozygote model: TT versus CC). Figure K in S3 File: Begg’s funnel plot of association between the rs2075800 polymorphism and NIHL (Dominant model: TT+CT versus CC). Figure L in S3 File: Begg’s funnel plot of association between the rs2075800 polymorphism and NIHL (Recessive model: TT versus CT+CC). Figure M in S3 File: Begg’s funnel plot of association between the rs2227956 polymorphism and NIHL (Heterozygote model: TC versus TT). Figure N in S3 File: Begg’s funnel plot of association between the rs2227956 polymorphism and NIHL (Homozygote model: CC versus TT). Figure O in S3 File: Begg’s funnel plot of association between the rs22279 [file pone.0188539.s003.zip › S3 File/Figure R.tif]

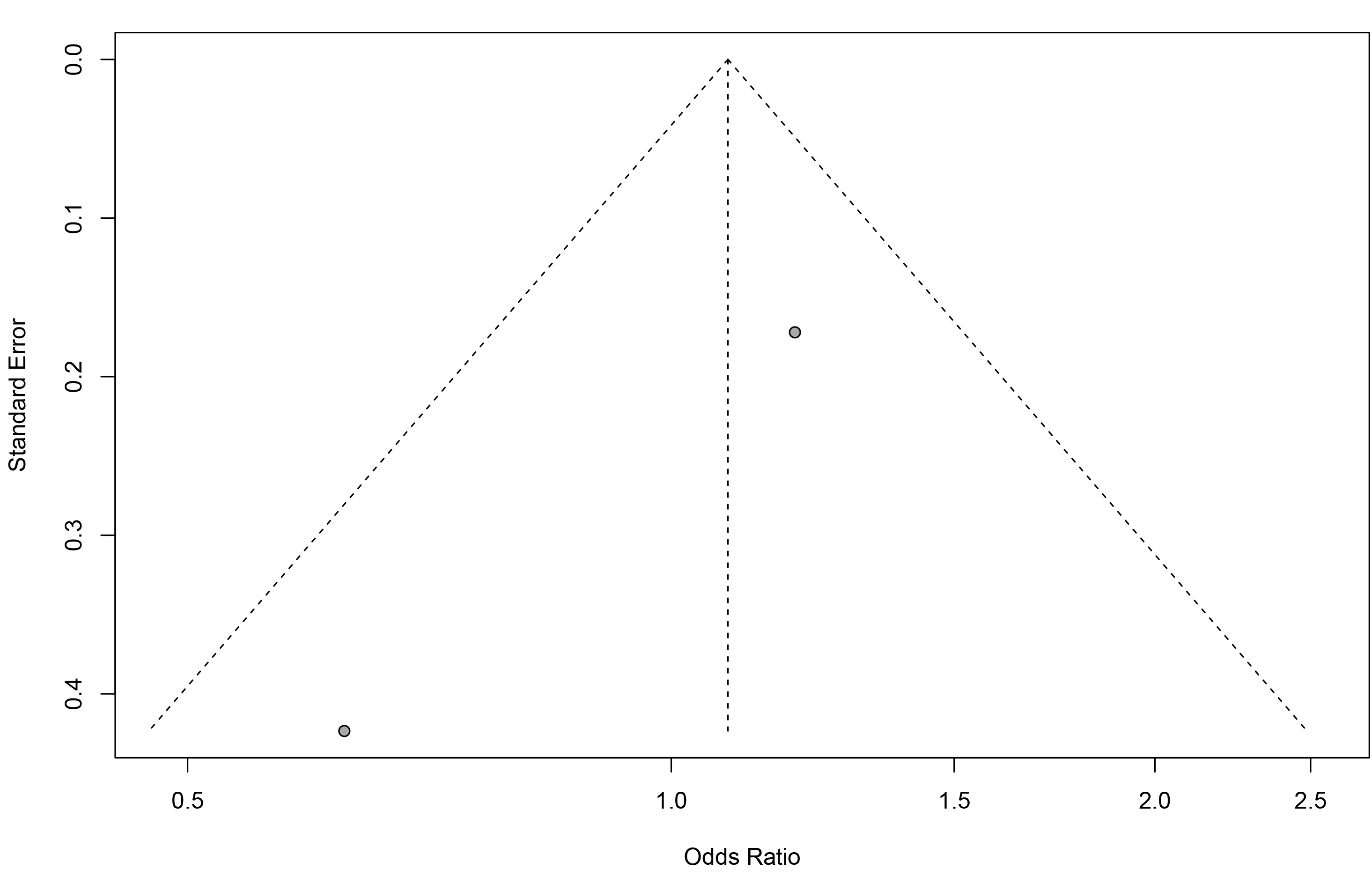

Supplement: S3 File — Begg’s funnel plot (Figure A—Figure T). (ZIP). Figure A in S3 File: Begg’s funnel plot of association between the rs1043618 polymorphism and NIHL (Heterozygote model: GC versus GG). Figure B in S3 File: Begg’s funnel plot of association between the rs1043618 polymorphism and NIHL (Homozygote model: CC versus GG). Figure C in S3 File: Begg’s funnel plot of association between the rs1043618 polymorphism and NIHL (Dominant model: CC+GC versus GG). Figure D in S3 File: Begg’s funnel plot of association between the rs1043618 polymorphism and NIHL (Recessive model: CC versus CG+GG). Figure E in S3 File: Begg’s funnel plot of association between the rs1061581 polymorphism and NIHL (Heterozygote model: AG versus AA). Figure F in S3 File: Begg’s funnel plot of association between the rs1061581 polymorphism and NIHL (Homozygote model: GG versus AA). Figure G in S3 File: Begg’s funnel plot of association between the rs1061581 polymorphism and NIHL (Dominant model: GG+AG versus AA). Figure H in S3 File: Begg’s funnel plot of association between the rs1061581 polymorphism and NIHL (Recessive model: GG versus AG+AA). Figure I in S3 File: Begg’s funnel plot of association between the rs2075800 polymorphism and NIHL (Heterozygote model: CT versus CC). Figure J in S3 File: Begg’s funnel plot of association between the rs2075800 polymorphism and NIHL (Homozygote model: TT versus CC). Figure K in S3 File: Begg’s funnel plot of association between the rs2075800 polymorphism and NIHL (Dominant model: TT+CT versus CC). Figure L in S3 File: Begg’s funnel plot of association between the rs2075800 polymorphism and NIHL (Recessive model: TT versus CT+CC). Figure M in S3 File: Begg’s funnel plot of association between the rs2227956 polymorphism and NIHL (Heterozygote model: TC versus TT). Figure N in S3 File: Begg’s funnel plot of association between the rs2227956 polymorphism and NIHL (Homozygote model: CC versus TT). Figure O in S3 File: Begg’s funnel plot of association between the rs22279 [file pone.0188539.s003.zip › S3 File/Figure S.tif]

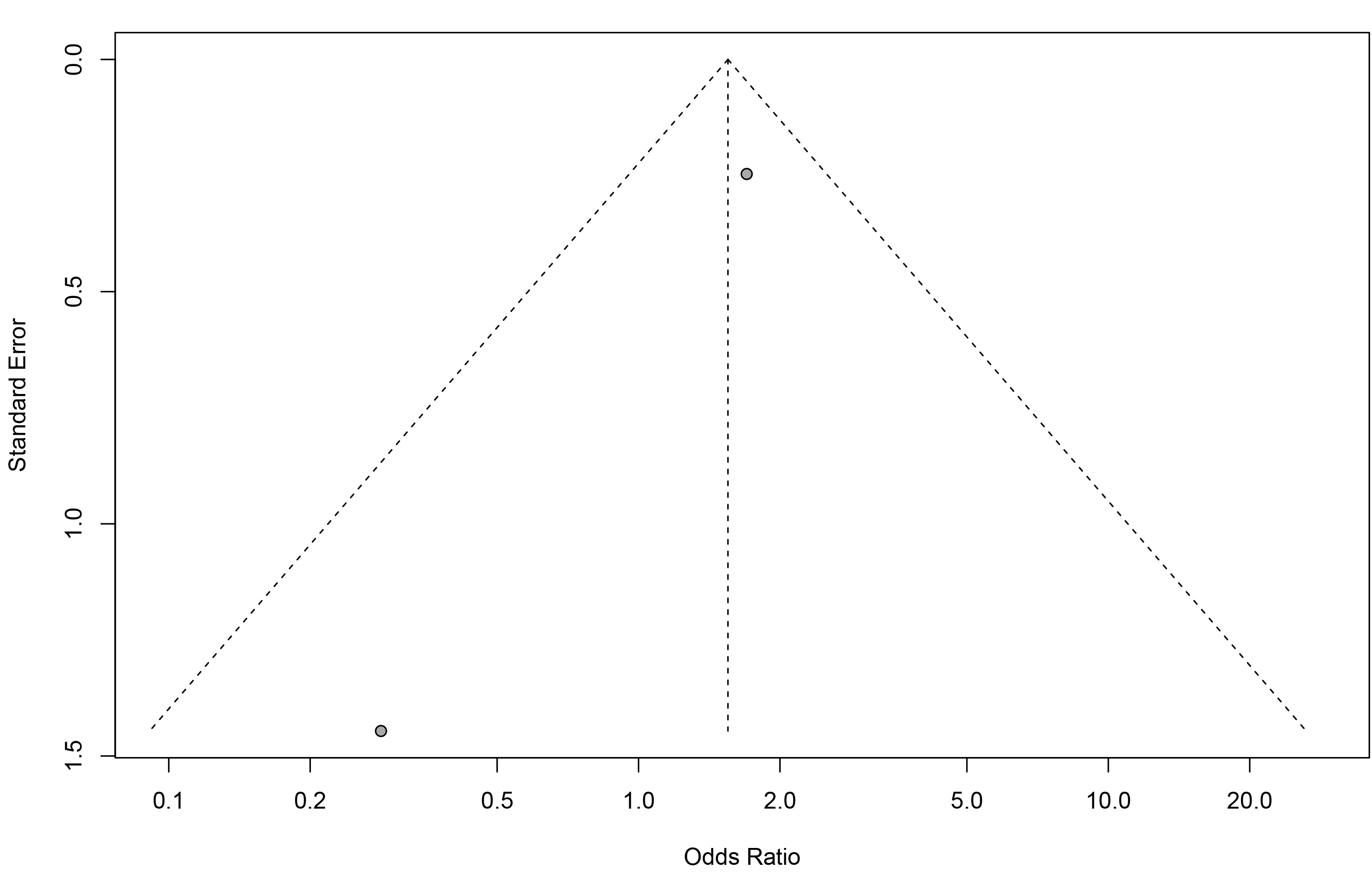

Supplement: S3 File — Begg’s funnel plot (Figure A—Figure T). (ZIP). Figure A in S3 File: Begg’s funnel plot of association between the rs1043618 polymorphism and NIHL (Heterozygote model: GC versus GG). Figure B in S3 File: Begg’s funnel plot of association between the rs1043618 polymorphism and NIHL (Homozygote model: CC versus GG). Figure C in S3 File: Begg’s funnel plot of association between the rs1043618 polymorphism and NIHL (Dominant model: CC+GC versus GG). Figure D in S3 File: Begg’s funnel plot of association between the rs1043618 polymorphism and NIHL (Recessive model: CC versus CG+GG). Figure E in S3 File: Begg’s funnel plot of association between the rs1061581 polymorphism and NIHL (Heterozygote model: AG versus AA). Figure F in S3 File: Begg’s funnel plot of association between the rs1061581 polymorphism and NIHL (Homozygote model: GG versus AA). Figure G in S3 File: Begg’s funnel plot of association between the rs1061581 polymorphism and NIHL (Dominant model: GG+AG versus AA). Figure H in S3 File: Begg’s funnel plot of association between the rs1061581 polymorphism and NIHL (Recessive model: GG versus AG+AA). Figure I in S3 File: Begg’s funnel plot of association between the rs2075800 polymorphism and NIHL (Heterozygote model: CT versus CC). Figure J in S3 File: Begg’s funnel plot of association between the rs2075800 polymorphism and NIHL (Homozygote model: TT versus CC). Figure K in S3 File: Begg’s funnel plot of association between the rs2075800 polymorphism and NIHL (Dominant model: TT+CT versus CC). Figure L in S3 File: Begg’s funnel plot of association between the rs2075800 polymorphism and NIHL (Recessive model: TT versus CT+CC). Figure M in S3 File: Begg’s funnel plot of association between the rs2227956 polymorphism and NIHL (Heterozygote model: TC versus TT). Figure N in S3 File: Begg’s funnel plot of association between the rs2227956 polymorphism and NIHL (Homozygote model: CC versus TT). Figure O in S3 File: Begg’s funnel plot of association between the rs22279 [file pone.0188539.s003.zip › S3 File/Figure T.tif]
