# Supplementary material for: Association between polymorphisms of heat-shock protein 70 genes and noise-induced hearing loss: A meta-analysis
Source: PLoS One. 2017 Nov 27;12(11):e0188539. doi: 10.1371/journal.pone.0188539 (PMC5703472; doi:10.1371/journal.pone.0188539)
Supplement: S1 File — (ZIP) [file pone.0188539.s001.zip › PRISMA 2009 Checklist.doc]

| **Section/topic** | | **#** | | **Checklist item** | **Reported on page #** |
| --- | --- | --- | --- | --- | --- |
| **TITLE** | | | | |  |
| Title | | 1 | | Association between polymorphisms of heat-shock protein 70 genes and noise-induced hearing loss: a meta-analysis | 1 |
| **ABSTRACT** | | | | |  |
| Structured summary | | 2 | | Background  Recent studies have evaluated the associations between polymorphisms of the heat-shock protein 70 (HSP70) encoding genes and NIHL. However, the conclusions of these studies are conflicting. The objective of this meta-analysis was to clarify the association between all known polymorphisms of HSP70 genetic loci and susceptibility to NIHL, based on existing reports.  Methods  We conducted a meta-analysis of the association between Hsp70 polymorphisms (rs1043618, rs1061581, rs2075800, rs2227956, and rs2763979) and NIHL risk in both Chinese and Caucasian males. All statistical analysis was done with was conducted using the “meta” package (version 4.6-0) of R version 3.3.2 and RStudio version 1.0.44. Online databases were searched for eligible case-control studies on February 13, 2017. The odds ratio (OR), 95% confidence interval (CI), and P value were calculated using Mantel-Haenszel statistics under a random- or fixed-effect model.  Results  A total of five studies, reported via four articles from online databases, were included in our meta-analysis. For rs1061581 (from three studies), a significant association was detected in the allele model, homozygote model, and dominant model (G versus A: OR (95% CI) = 1.32(1.05-1.67), GG versus AA: OR (95% CI) = 1.93(1.1-3.36), GG + AG versus AA: OR (95% CI) = 1.45(1.05-2.02)), but not in the heterozygote model or the recessive model. For rs1043618 (from five studies), rs2075800 (from two studies), rs2227956 (from four studies), rs2763979 (from two studies), no significant association was found for any genetic model. After subgroup analyses by ethnicity, significant associations were observed for the allele model, heterozygote model, and dominant model for rs1061581 and any genetic model for rs2227956 in Caucasian.  Conclusions  The rs1043618, rs2075800, and rs2763979 polymorphisms were not found to be associated with susceptibility to NIHL; however, the rs1061581 and rs2227956 polymorphisms were significantly associated with NIHL in Caucasian males.  Keywords: Noise-induced hearing loss, Heat-shock protein 70, Single Nucleotide Polymorphism, Meta-analysis | 2 |
| **INTRODUCTION** | | | | |  |
| Rationale | | 3 | | Noise-induced hearing loss (NIHL) is a preventable acquired hearing loss caused by industrial, military, and recreational noise exposure (Sha et al., 2017). NIHL is considered a serious public health problem; WHO estimates show that 10% of individuals worldwide that are exposed to noise may develop NIHL (Basner et al., 2014). Further, the prevalence of NIHL was 24.4% among US adults aged 20-69 years, according to the 2011–2012 National Health and Nutrition Examination Survey (Carroll et al., 2017). In addition, before 2010, 20% or more of the population in Asian countries was reported to suffer from NIHL; this rate was as high as 89% among individuals in specific occupations (Fuente et al., 2011). NIHL susceptibility is influenced by numerous genetic and environmental factors; in particular, heat-shock protein 70 genes (HSP70), which encode a group of proteins that protect cells from oxidative stress, are implicated. Other genes considered to influence NIHL susceptibility include inner ear potassium recycling pathway genes and monogenic deafness genes (Sliwinska-Kowalska et al., 2013).  Heat shock proteins (HSPs) are a class of functionally related proteins whose expression is stimulated by physiological stress, ototoxic drugs, high temperature, and noise (Li, 2015). HSP70, which is highly conserved and the most abundant chaperone, is present in various cellular compartments (Mashaghi et al., 2014; Saibil, 2013). The HSP70 gene family comprises HSP70-1, HSP70-2, and HSP70-hom (Sliwinska-Kowalska et al., 2013). Several studies have shown that the production of HSP70 in the inner ear protects hair cells by countering the ototoxic effects of cisplatin and aminoglycosides (Garcia-Berrocal et al., 2010; May et al., 2013; Taleb et al., 2009). The influence of HSP70 genetic polymorphisms on NIHL susceptibility has been the focus of investigations for over ten years (Konings et al., 2009; Li et al., 2017; Yang et al., 2006). However, these studies have been unable to reach a consistent conclusion. | 2-3 |
| Objectives | | 4 | | To our knowledge, there is no systematic review or meta-analysis of the association between HSP70 genetic variants and NIHL risk to date. The present report is the first meta-analysis, based on existing reports in the literature, of the relationship between all known HSP70 gene polymorphisms and susceptibility to NIHL. | 3 |
| **METHODS** | | | | |  |
| Protocol and registration | 5 | | A review protocol was developed a priori and registered in the PROSPERO International prospective register of systematic reviews (http://www.crd.york.ac.uk/PROSPERO; registration number CRD42017062595) | | 3 |
| Eligibility criteria | 6 | | The following eligibility criteria were applied in order to select studies for inclusion in the meta-analysis: (1) articles evaluating the association of the Hsp70 gene polymorphism with NIHL; (2) clear definition of NIHL; (3) case - control study; (4) published in either English or Chinese; (5) sufficient published data for calculating odds ratios (ORs) with their 95% confidence intervals (CIs)  The exclusion criteria were as follows: (1) duplicate publications (only the latest publication with the most complete or updated data was selected); (2) incomplete information; (3) insufficient data; (4) review articles or conference literatures.. | | 3-4 |
| Information sources | 7 | | The relevant studies were identified by searching PubMed, EMBASE, Web of Science, China National Knowledge Infrastructure (CNKI), Wanfang Data, and SinoMed (Chinese biomedical literature service system). | | 3 |
| Search | 8 | | The search terms were: (“Hearing Loss, Noise-Induced” or “Acoustic Trauma”) and (“HSP70 Heat-Shock Proteins” or “HSP70”) in combination with (“Polymorphism, Single Nucleotide” or “gene” or “polymorphism” or “variants or alleles”). The full search strategy is available on PROSPERO. The last search was conducted on February 13, 2017. | | 3 |
| Study selection | 9 | | A total of 34 potential articles were retrieved through electronic databases, including EMBASE (n = 5), PubMed (n = 7), Web of Science (n = 8), SinoMed (n = 1), Wanfang (n = 7), and CNKI (n = 6), during initial searching. The study selection process is detailed in Figure 1. After 12 duplicated articles were removed and 16 articles were excluded by screening the title and abstract, 13 articles were found to be unrelated and 3 articles were reviews (Clifford et al., 2016; Hu et al., 2014; Sliwinska-Kowalska et al., 2013). The remaining 6 articles were full-text-reviewed, and 2 were excluded: one (Li, 2015) of these was a repeat publication and the other (Yang et al.) did not contain sufficient data. Finally, 5 studies reported in 4 articles (Chang et al., 2011; Konings et al., 2009; Li et al., 2017; Yang et al., 2006) fulfilled the inclusion criteria and were included in the present meta-analysis (the article of Konings et al. reports 2 studies for Sweden and Poland). | | 4-5 |
| Data collection process | 10 | | Data were extracted by two reviewers (Song Lei and Liu Huang) independently according to the pre-specified data extraction form. The following information was extracted from each study: first author, population (country, ethnicity), source of controls, case/control sample size, genotype counts for cases and controls, and evidence of Hardy-Weinberg equilibrium (HWE). | | 4 |
| Data items | 11 | | Data, including name of the first author, year of publication, study population (country, ethnicity), source of controls, case/control sample size, minor allele frequency (MAF), genotype counts in the cases/controls, and evidence of Hardy–Weinberg equilibrium (HWE), the population number, the mean of HDL-C level and its SD by genotypes, were extracted from each study. | | 4 |
| Risk of bias in individual studies | 12 | | The Newcastle-Ottawa Scale (NOS) was used to assess the methodological quality of the individual studies by the two reviewers (Song Lei and Yaqian Liu)(Wells et al.). Each study was evaluated and scored based on three criteria: selection (4 stars), comparability (2 stars), and exposure (3 stars). The NOS point ranged from 0 to 9 stars. Disagreement, if any, was resolved by discussion with a third reviewer (Lei Yang). | | 4 |
| Summary measures | 13 | | The P value, odds ratio (OR) and corresponding 95% confidence interval (CI) were calculated using Mantel-Haenszel statistics under the allele, homozygote, heterozygote, dominant or recessive models. | | 4 |
| Synthesis of results | 14 | | We first assessed the HWE in the control group using the chi-square test(Schaid et al., 1999). The P value, odds ratio (OR) and corresponding 95% confidence interval (CI) were calculated using Mantel-Haenszel statistics under the allele, homozygote, heterozygote, dominant or recessive models. This differs from the protocol as there is no explicit additive model; therefore, this was replaced with homozygote and heterozygote models. P values of less than 0.05 were considered to represent statistically significant associations between HSP70 gene polymorphisms and NIHL. Heterogeneity across individual studies was analyzed by the Cochran’s-Q statistic and the I2 statistic (P ＜ 0.10 and I2 ＞50% indicated the significance of heterogeneity)(Higgins et al., 2003; Zintzaras et al., 2005). A fixed-effect model was selected with no significant heterogeneity among studies. Otherwise, a random-effect model was used (Higgins et al., 2002; Higgins et al., 2003; Thakkinstian et al., 2005; Zintzaras et al., 2005). | | 4 |

Page 1 of 2

| **Section/topic** | **#** | **Checklist item** | **Reported on page #** |
| --- | --- | --- | --- |
| Risk of bias across studies | 15 | The potential publication bias was evaluated by Begg’s funnel plot and Egger’s test (Egger et al., 1997). | 4 |
| Additional analyses | 16 | Subsequently, subgroup analyses were performed based on ethnicity to explore the sources of heterogeneity. | 4 |
| **RESULTS** | | |  |
| Study selection | 17 | A total of 34 potential articles were retrieved through electronic databases, including EMBASE (n = 5), PubMed (n = 7), Web of Science (n = 8), SinoMed (n = 1), Wanfang (n = 7), and CNKI (n = 6), during initial searching. The study selection process is detailed in Figure 1. After 12 duplicated articles were removed and 16 articles were excluded by screening the title and abstract, 13 articles were found to be unrelated and 3 articles were reviews (Clifford et al., 2016; Hu et al., 2014; Sliwinska-Kowalska et al., 2013). The remaining 6 articles were full-text-reviewed, and 2 were excluded: one (Li, 2015) of these was a repeat publication and the other (Yang et al.) did not contain sufficient data. Finally, 5 studies reported in 4 articles (Chang et al., 2011; Konings et al., 2009; Li et al., 2017; Yang et al., 2006) fulfilled the inclusion criteria and were included in the present meta-analysis (the article of Konings et al. reports 2 studies for Sweden and Poland). | 4-5 |
| Study characteristics | 18 | The selected study characteristics and data are listed in Table 1. Various genotyping methods were utilized, including PCR-RFLP, TaqMan, and SNPscan. | 5-7 |
| Risk of bias within studies | 19 | The NOS results were shown in Table 1. The NOS scores of all studies ranged from 8 to 9 stars. | 5-7 |
| Results of individual studies | 20 | The main results of individual studies were shown in Table 2, Figure. 2 - Figure. 6 and Figure S1 – Figure S20. | 8-12 |
| Synthesis of results | 21 | The genetic association between the rs1043618 polymorphism and susceptibility to NIHL was measured. The P value of Cochran’s-Q statistic for all genetic models was greater than 0.1, and I2 was less than 50%, suggesting that there was no obvious heterogeneity between the studies; therefore, the fixed-effect model was selected for pooling data. Overall, no significant association was found for any genetic model (C versus G: OR (95% CI) = 1.15(0.98-1.35), GC versus GG: OR (95%CI) = 1.15(0.91-1.45), CC versus GG: OR (95% CI) = 1.30(0.91-1.86), CC + GC versus GG: OR (95% CI) = 1.19(0.95-1.48), CC versus CG + GG: OR (95% CI) = 1.23(0.88-1.72)).  We additionally pooled analyses of the rs1061581 polymorphism and NIHL; these are shown in Figure 3 and Table 2. The P value of Cochran’s-Q statistic of all genetic models were greater than 0.1 and I2 was less than 50%, indicating that there was no significant heterogeneity between the studies; therefore, the fixed-effect model was implemented. Overall, significant associations were detected in the allele model, homozygote model, and dominant model (G versus A: OR (95% CI) = 1.32(1.05-1.67), GG versus AA: OR (95% CI) = 1.93(1.1-3.36), GG + AG versus AA: OR (95% CI) = 1.45(1.05-2.02)), but not in the heterozygote model or recessive model (AG versus AA: OR(95% CI) = 1.38(0.98-1.94) , GG versus AG + AA: OR (95% CI) = 1.50(0.90-2.50)).  The association between the rs2075800 polymorphism and the risk of NIHL was analyzed in two Chinese studies. The P value of Cochran’s-Q statistic for all genetic models was greater than 0.1, and that of I2 less than 50%, indicating that there was no significant heterogeneity between the studies; therefore, the fixed-effect model was applied. There were no notable associations for any of the genetic models (T versus C: OR (95% CI) = 0.81(0.65-1.02), CT versus CC: OR (95% CI) = 0.87(0.63-1.21), TT versus CC: OR (95% CI) = 0.61(0.37-1.01), TT + CT versus CC: OR (95% CI) = 0.81(0.60-1.10), TT versus CT + CC: OR (95%CI) = 0.66(0.41-1.06)) (Figure 4 and Table 2).  Data describing the association between the rs2227956 polymorphism and the risk of NIHL are shown in Figure 5 and Table 2. The P value of Cochran’s-Q statistic of heterozygote model and dominant model was greater than 0.1, and that of I2 was less than 50%; therefore, the fixed-effect model was used. However, for the allele model (P = 0.04, I2 = 63.7%), homozygote model (P = 0.05, I2 = 62.4%), and recessive model (P = 0.06, I2 = 60.3%), the random-effect model was used. Overall, no significant association was found for any genetic model (C versus T: OR (95% CI) = 0.78(0.53-1.15), TC versus TT: OR (95% CI) = 0.80(0.62-1.04), CC versus TT: OR (95% CI) = 0.55 (0.09-3.35), CC + TC versus TT: OR (95% CI) = 0.80(0.62-1.03), CC versus TC + GG: OR (95% CI) = 0.80(0.35-1.80)).  Besides above four genetic loci, Figure 6 and Table 2 show the pooled data of the association between the rs2763979 polymorphism and NIHL susceptibility; the 2 studies of this polymorphism only focused on the Chinese population. The P value of Cochran’s-Q statistic was lower than 0.1, and that of I2 was more than 50% for the allele model (P = 0.06, I2 = 71.7%); therefore, we applied the random-effect model to this genetic model and the fixed-effect model to other models. There were no notable associations for all genetic models (T versus C: OR (95% CI) = 0.94(0.46-1.91), CT versus CC: OR (95% CI) = 1.00(0.72-1.39), TT versus CC: OR (95% CI) = 1.56(0.95-2.56), TT + CT versus CC: OR (95% CI) = 1.08(0.80-1.48), TT versus CT + CC: OR (95% CI) = 1.55(0.98-2.46)). | 8-12 |
| Risk of bias across studies | 22 | There was no publication bias with regards to the association between rs1043618, rs1061581, and rs2227956 polymorphisms and NIHL susceptibility, as identified using the Begg’s funnel plot or Egger’s regression test (Table 3). Funnel plots of the above 3 genetic loci in all genetic models were symmetrical (Figure 7). Owing to the limited number of studies of rs2075800 and rs2763979 included, funnel plot analysis and the Egger’s test were not carried out. | 12-13 |
| Additional analysis | 23 | **Subgroup analyses:** For the association between the rs1043618 polymorphism and susceptibility to NIHL, subgroup analyses by ethnicity showed no significant association in the Chinese population; this outcome was consistent with that observed in Caucasians.  For the association between the rs1061581 polymorphism and susceptibility to NIHL, subgroup analyses by ethnicity showed no significant association in the Chinese population; however, significant associations were observed for the allele model, heterozygote model, and dominant model in Caucasians.  For the association between the rs2227956 polymorphism and susceptibility to NIHL, After subgroup analyses by ethnicity, the P value of Cochran’s-Q statistic of all models was greater than 0.1, and that of I2 was less than 50% in Chinese and Caucasian populations. In addition, the association for all genetic models was significant in the Caucasian population but not in the Chinese.  The main subgroup analysis results are shown in detail in Table 2. | 8-12 |
| **DISCUSSION** | | |  |
| Summary of evidence | 24 | In the present meta-analysis, we evaluated the association between SNPs in the following HSP70 genes: HSP70-1 (rs1043618, 615 cases and 925 controls), HSP70-2 (rs1061581, 301 cases and 315 controls; rs2763979, 313 cases and 608 controls), HSP70-hom (rs2075800, 303 cases and 608 controls; rs2227956, 578 cases and 592 controls) and NIHL. Overall analysis indicated that there was no association between rs1043618, rs2075800, rs2227956, and rs2763979 and NIHL susceptibility for any of the genetic models. However, we identified a positive relationship between rs1061581 and NIHL in the allele model, homozygote model, and dominant model. | 13 |
| Limitations | 25 | **Limitations:** The present study also has several limitations. For instance, the number of included studies were very few for rs2075800 and rs2763979, which limited further analysis. Secondly, Yang, M’s study of rs161581 was not in accordance with HWE; however, subgroup analysis was used separately. Thirdly, we were unable to extract sufficient adjustment data per age, noise exposure level, and so on. Finally, as with three other meta-analysis studies (Wang et al., 2017; Yu et al., 2016; Zhou et al., 2014) of the association between NIHL and other SNPs, our meta-analysis involved a small sample size, resulting in unstable pooled results. | 14 |
| Conclusions | 26 | **Conclusion：**In summary, our meta-analysis comprehensively and systematically evaluated the association between HSP70 gene polymorphisms and NIHL susceptibility. The results suggested that rs1043618, rs2075800, and rs2763979 polymorphisms are not associated with susceptibility to NIHL, while rs1061581 and rs2227956 polymorphism is significant associated with NIHL in Caucasians males. Given the limited sample size, the conclusions of this study should be treated with caution, and large sample studies are necessary in the future. | 15 |
| **FUNDING** | | |  |
| Funding | 27 | This work was supported by Zhejiang Key Research and Development Program(grant number 2015C03050), the Major Science and Technology Innovation Project of Hangzhou in China(grant number 20152013A01). | Online submission system |
