# Supplementary figures and images for: Correction: Association between polymorphisms of heat-shock protein 70 genes and noise-induced hearing loss: A meta-analysis
Source: PLoS One. 2020 Nov 17;15(11):e0242648. doi: 10.1371/journal.pone.0242648 (PMC7671518; doi:10.1371/journal.pone.0242648)

## Allele model

rs2227956

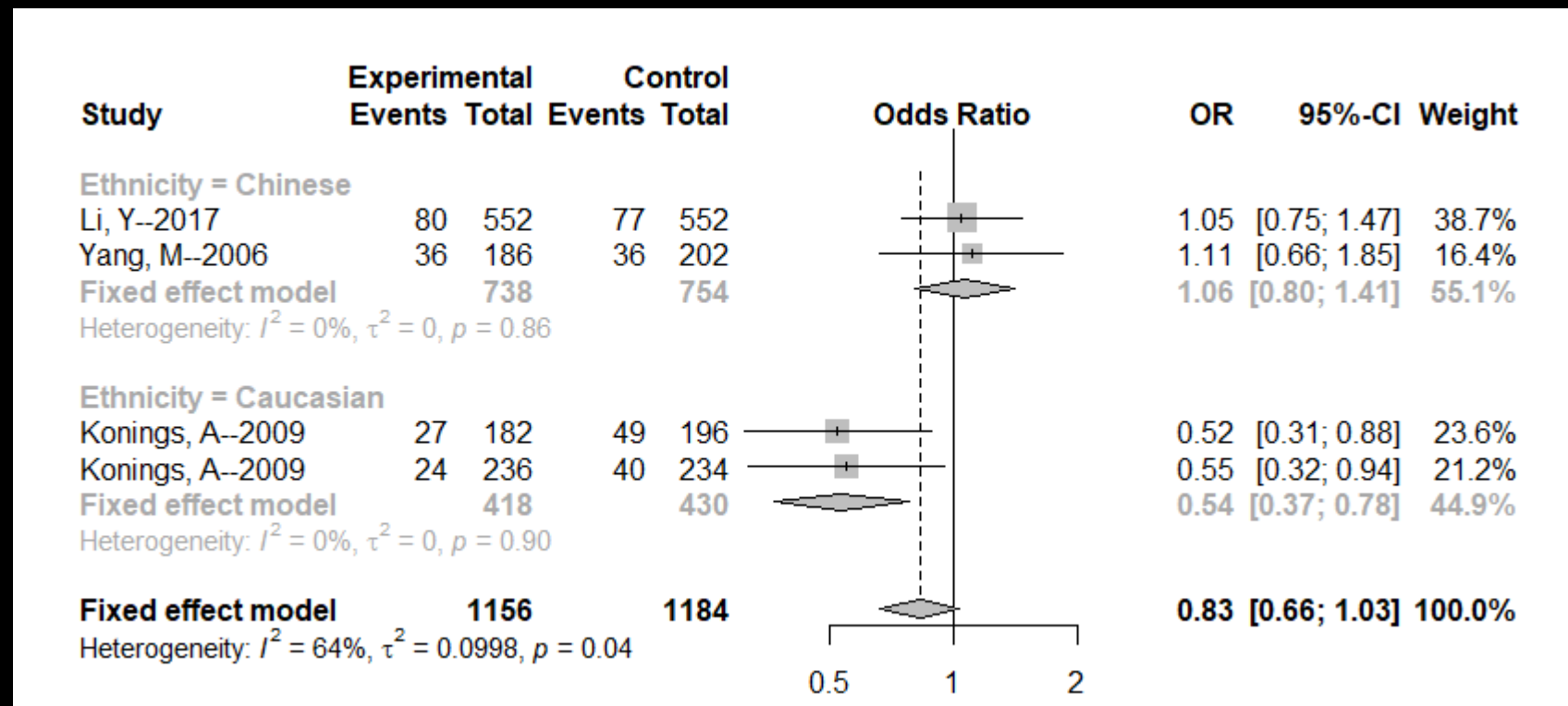

## Homozygote model

rs2763979

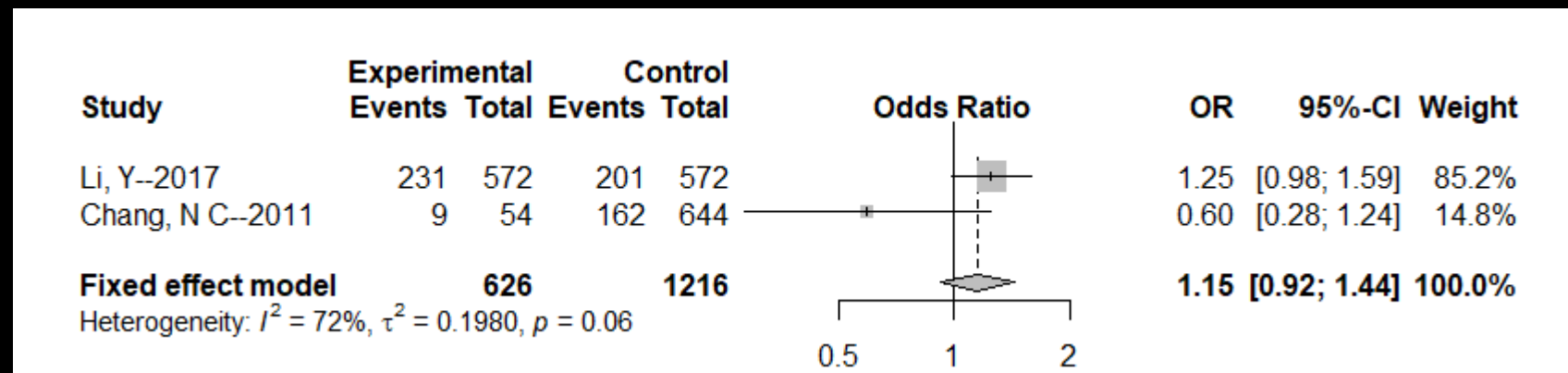

## Recessive model

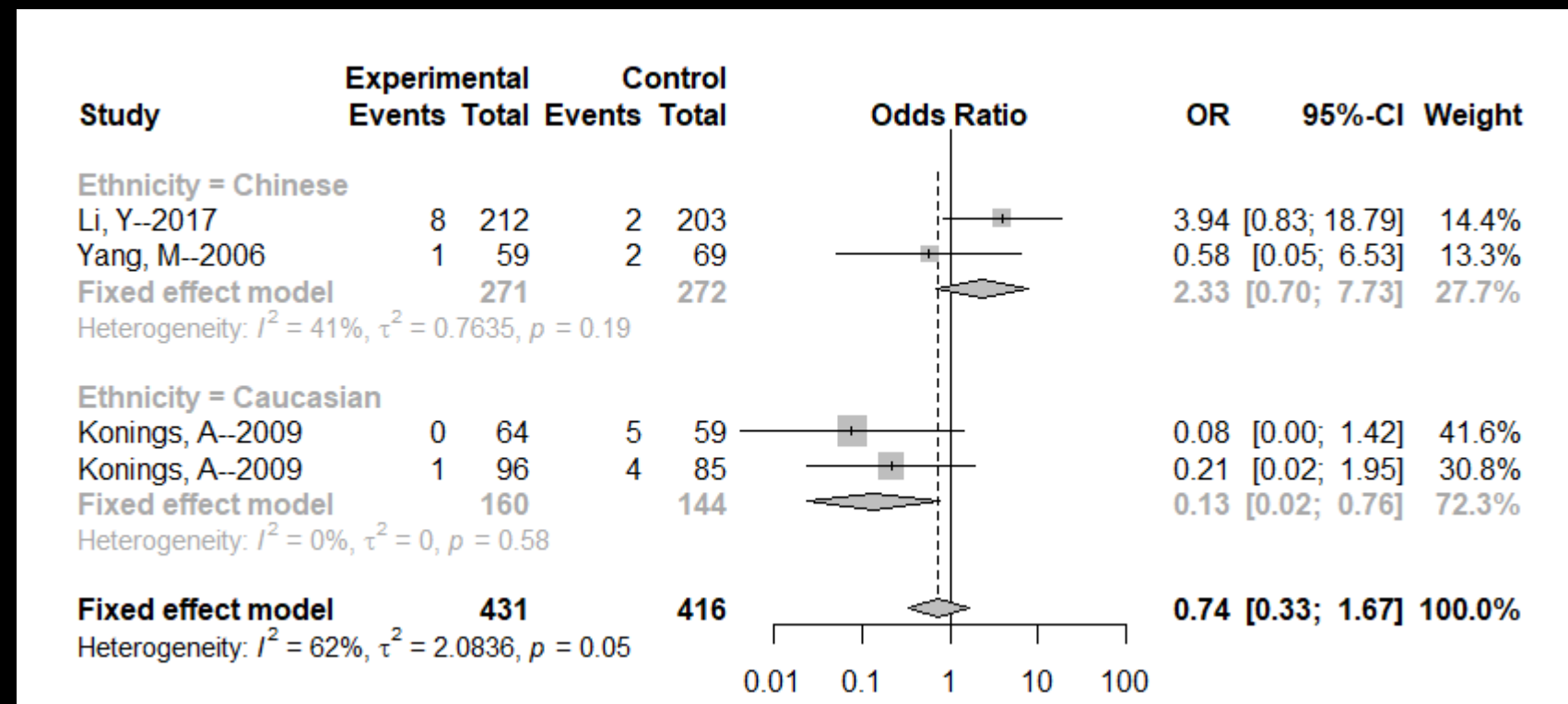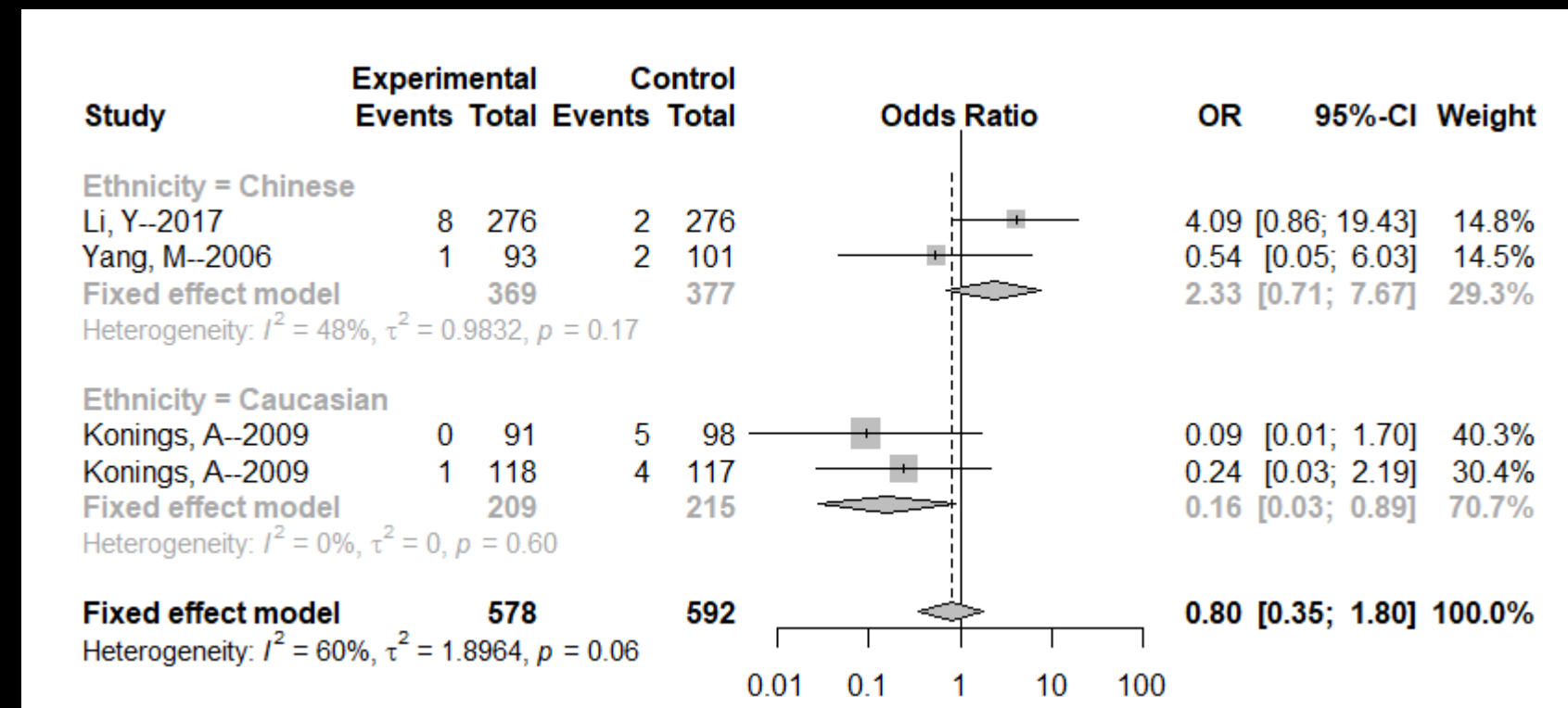

Supplement: S3 File — (PDF) [file pone.0242648.s003.pdf]

rs1043618

## Allele model

## Heterozygote model

## Homozygote model

## Dominant model

## Recessive model

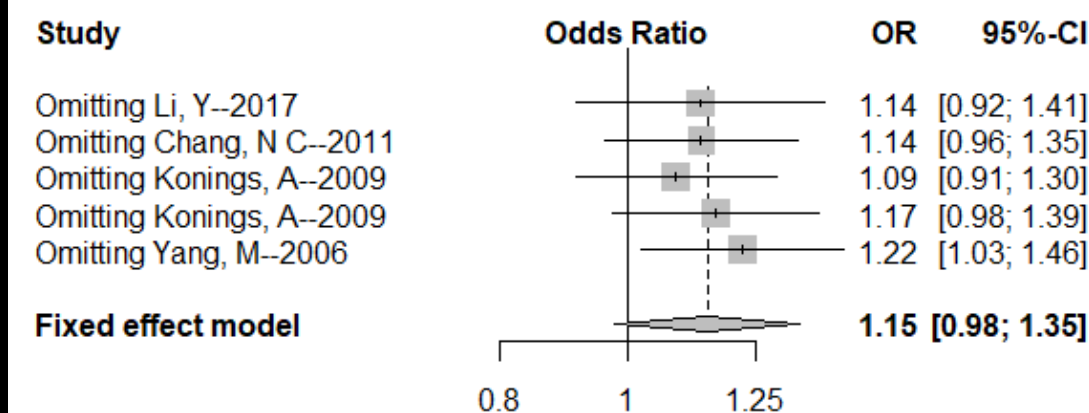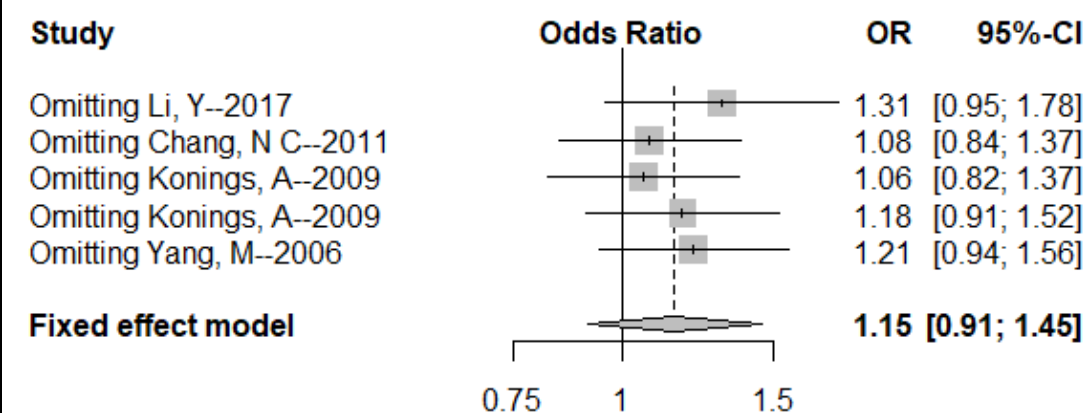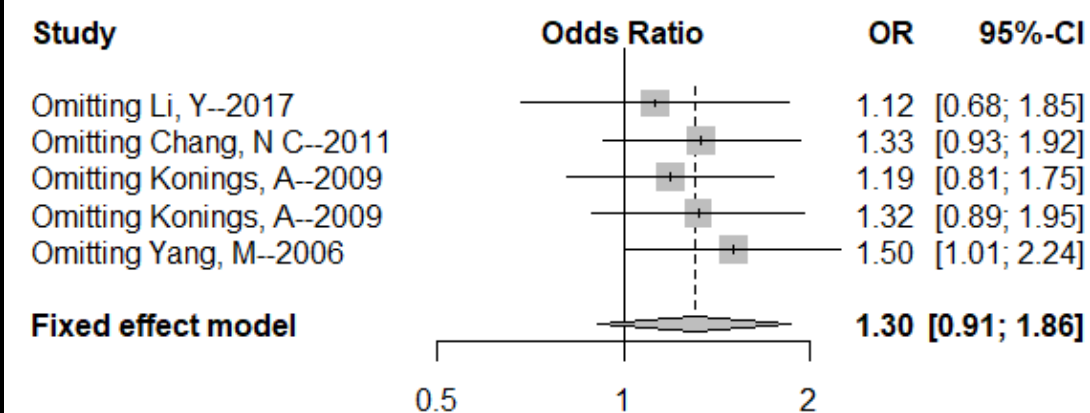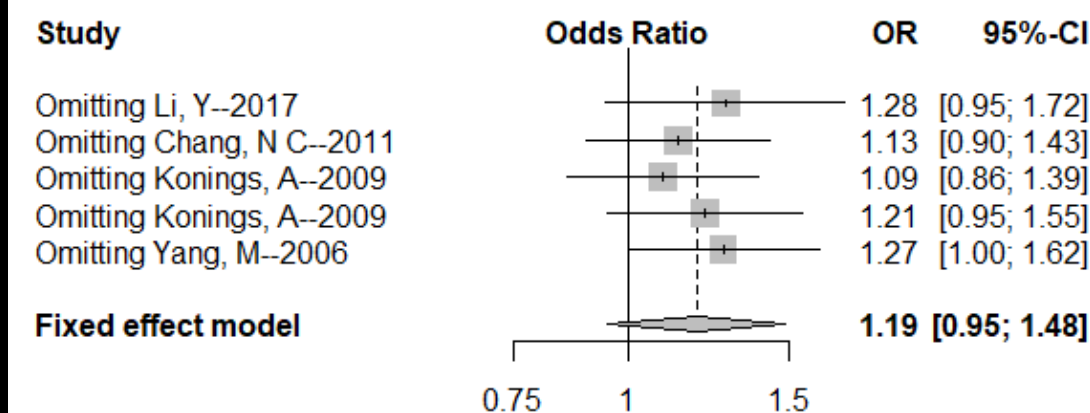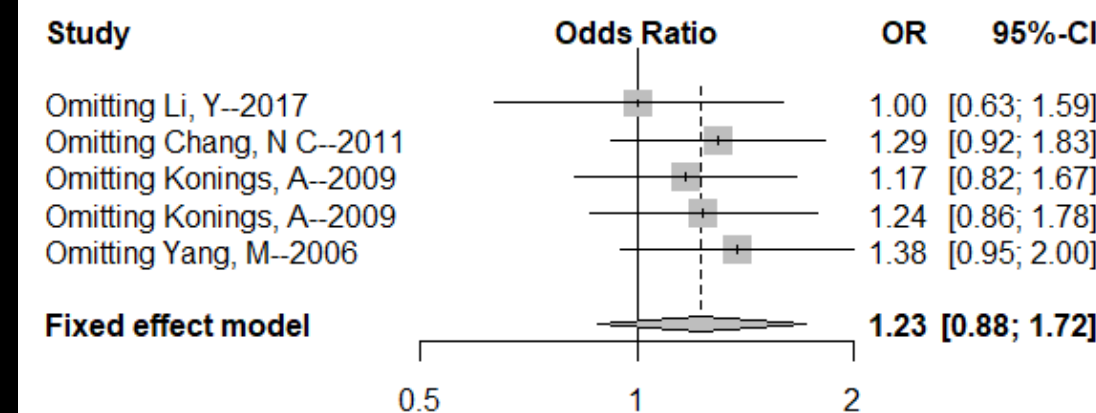

rs1061581

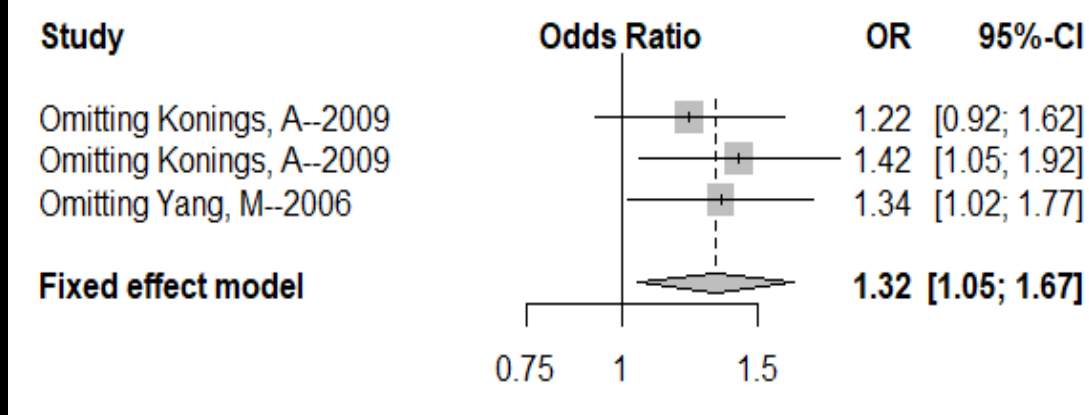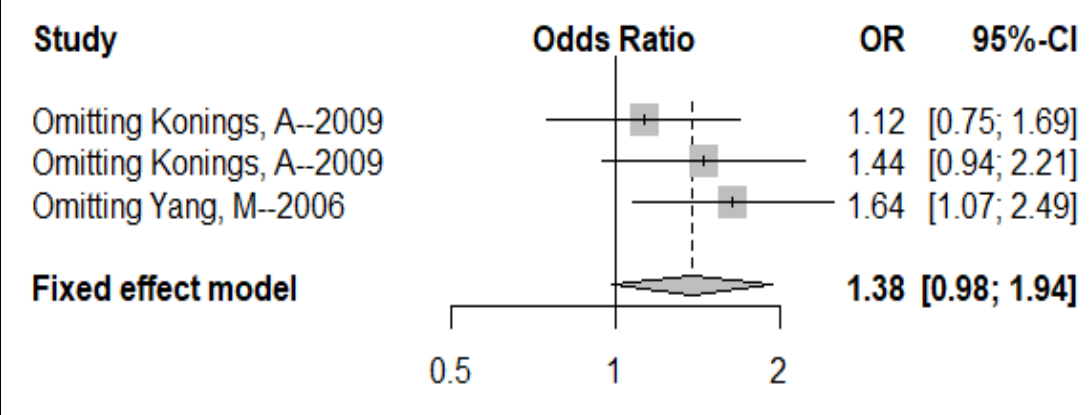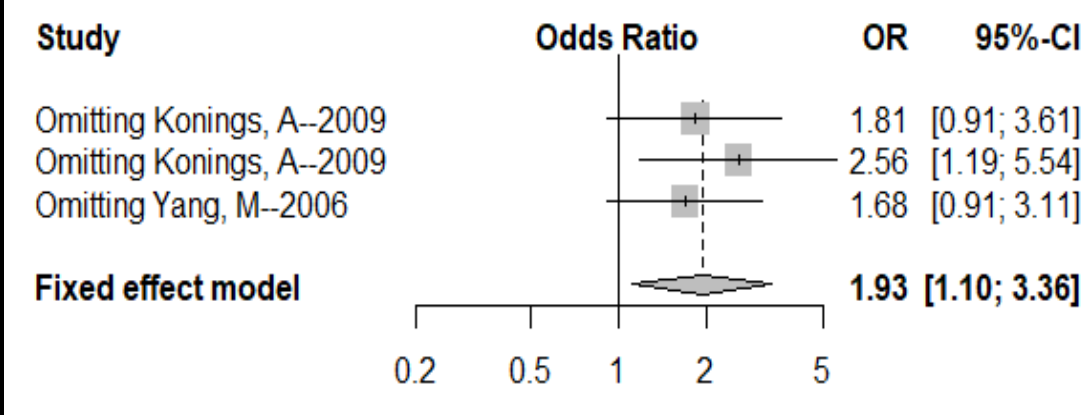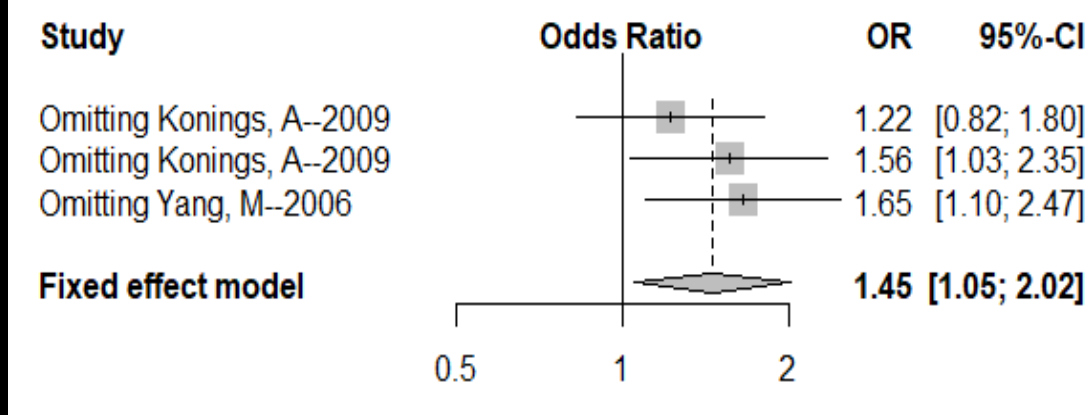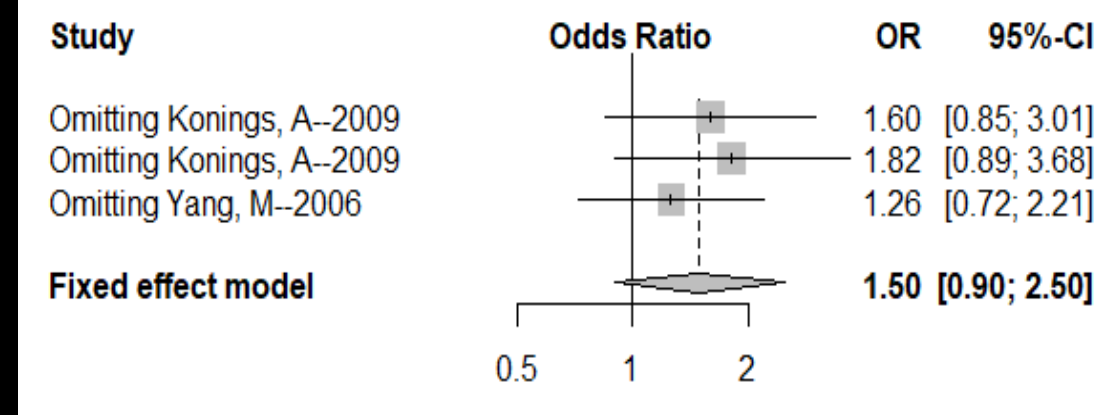

rs2227956

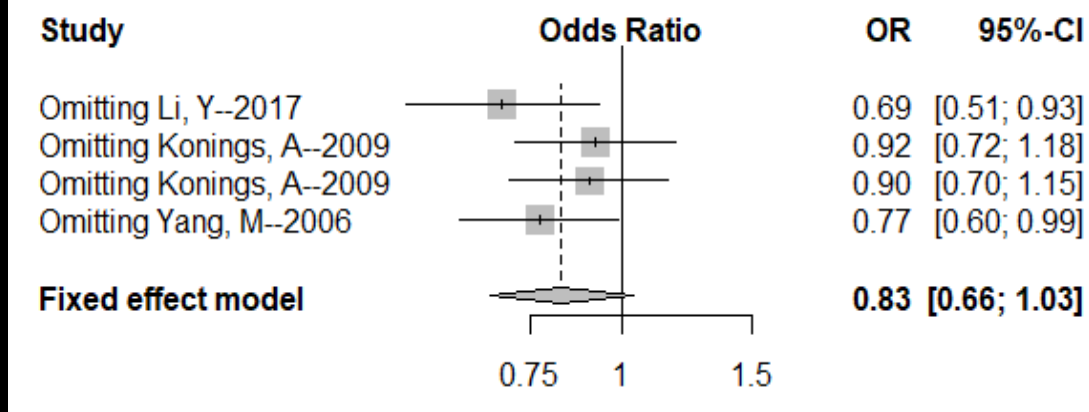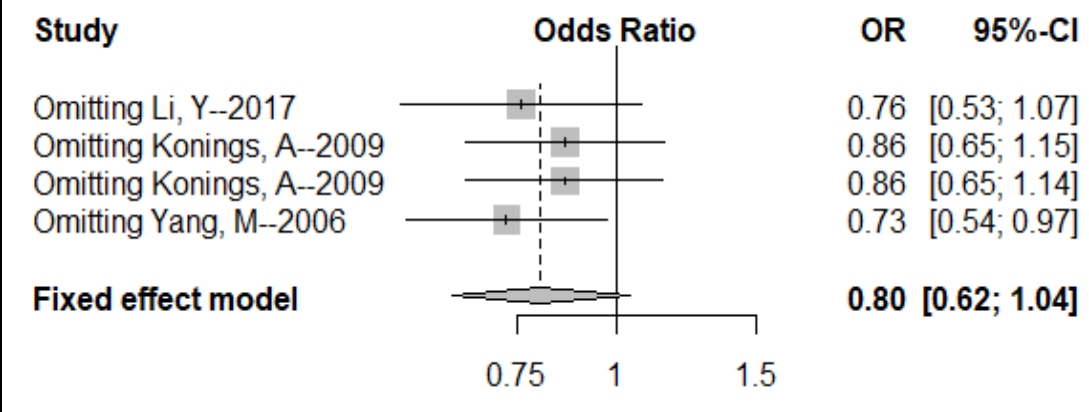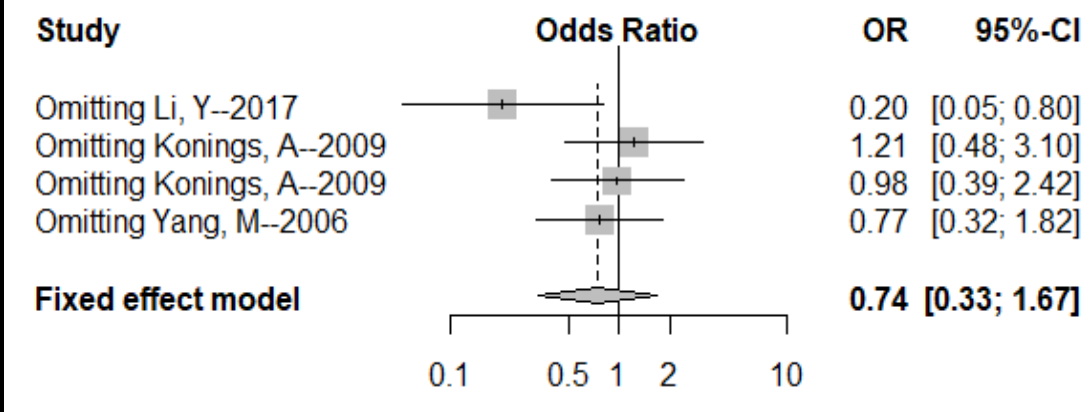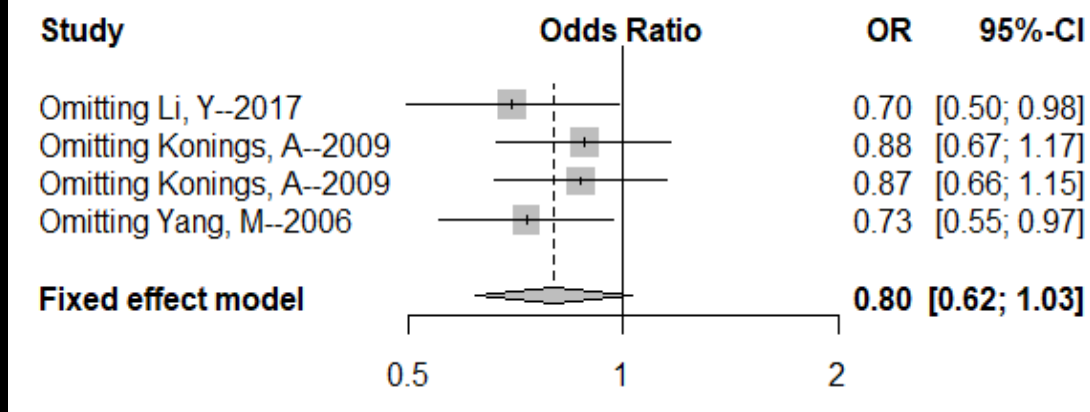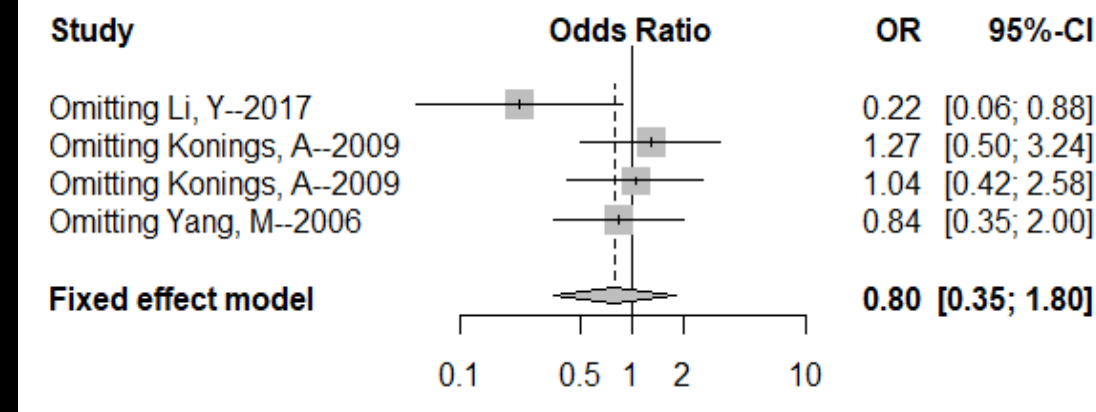

Supplement: S5 File — (PDF) [file pone.0242648.s005.pdf]
