# Supplementary material for: Correction: Association between polymorphisms of heat-shock protein 70 genes and noise-induced hearing loss: A meta-analysis
Source: PLoS One. 2020 Nov 17;15(11):e0242648. doi: 10.1371/journal.pone.0242648 (PMC7671518; doi:10.1371/journal.pone.0242648)
Supplement: S4 File — (DOCX) [file pone.0242648.s004.docx]

### Sensitivity analysis

We performed sensitivity analysis to examine the influence of each individual study on the pooled ORs by deleting each study one at a time from the pooled analysis of the SNPs (**rs1043618**, **rs1061581** and **rs2227956**), see **R8 figure**.  We found that after the study of exclusion of Li, Y 2007, the pooled ORs changed greatly from 0.74(0.33-1.82) to 0.20(0.05-0.80) in Homozygote model and from 0.80(0.35-1.80) to 0.22(0.06-0.88) in Recessive model of **rs2227956**. The reason for the instability of the above results may be due to the small sample size of the individual studies in the two models (Homozygote and Recessive model) and there are not enough cases (the case with CC genotype is only 8, 1, 0, 1 in respectively shown in **R6 figure**.). However, there is no obvious change in others genetic model (except Homozygote and Recessive model) of **rs2227956** and all genetic model of **rs1043618** and **rs1061581**. These findings show that our results were relatively robust for both of three polymorphisms expect Homozygote and Recessive model genetic models in **rs2227956**. For **rs2075800** and **2763979**, we did not perform the sensitivity analysis because of the limited number of included studies.
